# Toward Sustainable Radioactive Waste Management: Geopolymerization of Sewage Sludge Ash as a Viable Solution

**DOI:** 10.1021/acsomega.4c07195

**Published:** 2025-02-19

**Authors:** Alexandre Las Casas, Leandro Goulart de Araujo, Roberto Vicente, Júlio Takehiro Marumo

**Affiliations:** Nuclear and Energy Research Institute, IPEN-CNEN/SP, Av. Prof. Lineu Prestes, São Paulo 2242, Brazil

## Abstract

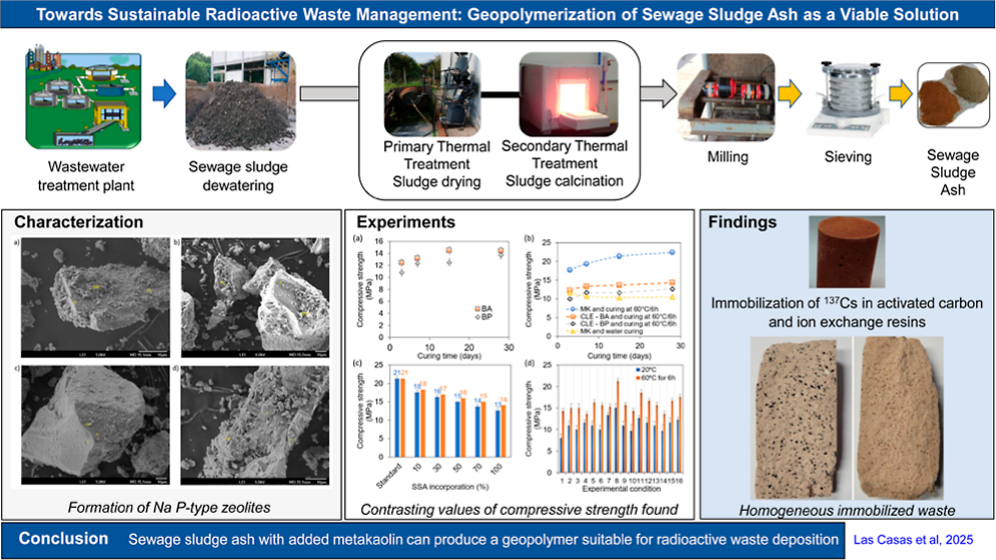

This study examines the geopolymerization potential of sewage sludge ash (SSA) for immobilizing radioactive waste through a series of experimental phases. The initial phase of the study involved processing sewage sludge from three different treatment plants, followed by calcination and subsequent characterization. The initial synthesis of geopolymers was conducted using 100% SSA, followed by compressive strength testing. In the second phase, a full factorial design was employed to optimize a metakaolin-based geopolymer formulation, with adjustments made to five variables: metakaolin (MK), activating solution (AS), sand, water, and lime. The optimal conditions were identified as 120 g MK, 125 g AS, 360 g sand, 55 g water, and 14.5 g lime. Under these conditions, the compressive strength increased from 15.0 ± 1.0 to 21.3 ± 0.6 MPa when the specimens were cured at 60 °C for 6 h. The optimized formulation was then augmented with SSA, and its characteristics were examined through a series of analytical techniques, including ICP–OES, XRF, XRD, SEM, and EDS. In the third phase of the study, immobilization of simulated radioactive activated carbon and ion-exchange resin wastes contaminated with ^137^Cs within the geopolymer matrix was investigated. Leaching and compressive strength tests were conducted to evaluate the performance of the material, and the results indicated that the release rates of ^137^Cs were between 2.55 × 10^–5^ and 3.23 × 10^–5^ cm d^–1^. These findings suggest that SSA-derived geopolymers can effectively immobilize radioactive waste, offering a sustainable alternative to traditional Portland cement.

## 1 Introduction

Efforts have been made in the field of radioactive waste treatment to find new materials that could eventually replace Portland cement. The aim is to find more resistant and suitable materials for immobilizing radioactive waste, which are sustainable and cost-competitive.^1^ Among the possible materials for immobilizing radioactive waste are geopolymers, inorganic polymers formed from the polycondensation of mineral raw materials containing aluminosilicates, forming a resistant chemical structure.^2^ It is defined as an activated material in a basic medium and is produced by reacting aluminosilicate materials with an alkaline activator, which is usually a concentrated alkaline hydroxide, silicate, carbonate or sulfate.^2^

Geopolymers can exhibit different and attractive properties, such as high compressive strength, acid resistance, and fire resistance.^3−5^ In addition to having mechanical strength similar to conventional cements, geopolymers are resistant to high temperatures, thermal shocks, chemical corrosion, and abrasion.^6,7^ These properties allow this binder to be used in a wide variety of applications.

In addition to these competitive advantages, geopolymers are considered by many researchers to be an environmentally sustainable material. However, some types of geopolymers have shown little impact on global warming when compared to Portland cement.^8^ This is because other parameters that are important in terms of environmental impacts, such as the impact of the production of sodium silicate solutions,^8^ have been shown to be more affected in geopolymer production.

In this context, the use of ash and other nonrecyclable materials may be advantageous as it requires fewer of these solutions. This highlights the need to explore new technologies that utilize waste that would otherwise be discarded as garbage. However, it is important to ensure that this waste has an adequate Si/Al oxide ratio to minimize the amount of sodium silicate required for the polymerization reaction. Geopolymer concretes can achieve a reduction of more than 40% in greenhouse gas emissions.^9^

Many aluminosilicate-based materials have been used in the literature to produce geopolymers, such as the synthetic metakaolin (MK),^10^ materials of natural origin (volcanic ash, diatomaceous earth)^11,12^ and waste (blast furnace slag, ceramics, glass, fly ash, mining waste, and sewage ash).^13−18^ Sewage sludge ash (SSA) is generated from the combustion of sewage sludge from sewage treatment plants. For instance, the production of sewage sludge in Brazil is estimated at 1.2 × 10^6^ t year^–1^, which represents a daily generation of 15 g per capita.^19^ The main components of the SSA are SiO_2_, Al_2_O_3_, Fe_2_O_3_, CaO, MgO, and P_2_O_5_.^15^ Its potential application as a precursor in geopolymeric systems is recent and has been the focus of study in recent years, such as in the solidification of heavy metals,^20,21^ radionuclide solidification,^16^ optimization of SSA mixing with MK,^14,22^ among others.

As a way of improving the properties of geopolymers, lowering costs or making them more environmentally attractive, various wastes have been used to make these materials with a focus on immobilizing radioactive waste. Some examples of this waste are slag and MK. Granulated blast furnace slag has already been used as one of the raw materials in the preparation of geopolymers for the solidification of simulated radioactive ion-exchange resin (IER).^23^ These authors used slag to prepare the activated alkaline materials and were able to immobilize significant quantities of IERs, with loadings of 45%. It is important to note that IERs are difficult to immobilize because they have expansion and contraction properties, making it difficult to load more than 10% into the solid matrix.^24^

In an additional example of resin application as radioactive waste, it was possible to immobilize borate-loaded resins with a loading efficiency of 35%.^25^ The substrate used by these researchers was cement slag, which was previously activated with alkaline solutions. The reactivity of activated cement slag proved to be suitable for immobilizing borate-loaded resins. However, Ahn et al.^26^ used a MK-based geopolymer to immobilize radioactive waste with a high concentration of sulfate ions, and were able to solidify 53.8% of this waste. In fact, the use of MK-based geopolymers has been widely used in the solidification of various wastes, in addition to IER wastes, such as sludge and liquid radioactive wastes, like those from nuclear power plants.^27,28^ In this context, if the geopolymer is manufactured for a specific application, such as the immobilization of radioactive waste, it can be used more efficiently than cement which, despite its low cost, may not meet the waste disposal standards because it does not have the right immobilization conditions.

The properties of geopolymers for the immobilization of radioactive waste are contingent upon a number of key parameters. The Si/Al ratio affects the geopolymer’s framework and chemical stability, thereby influencing its capacity to encapsulate radioactive particles.^3,4^ The concentration of alkaline activators (e.g., NaOH, KOH) affects the reaction rate, strength, and porosity, necessitating a balanced range for optimal reactivity.^2^ The curing temperature and time are instrumental in determining the crystallinity and mechanical properties of the material.^4,5,29^ While higher temperatures can facilitate the reaction, they may also result in cracking. The water-to-solid ratio exerts control over the workability, setting time, and pore structure of the material,^4,6,30^ which in turn affects its leachability. The incorporation of lime, sand, and varying quantities of precursors, including ash, sludge, and MK, can augment structural stability.^30^ The particle size of the ash influences the reaction rate and packing density, with smaller particles enhancing reactivity.^7^ The waste loading level is pivotal for leaching resistance and structural integrity, with optimal levels ensuring safe containment. By meticulously adjusting these parameters, researchers can enhance the durability and performance of the material for effective radioactive waste immobilization.^8,9^

Despite the growing interest in the use of geopolymers for the immobilization of radioactive waste, a number of significant challenges remain unresolved. Recent studies indicate that, although geopolymers have the potential for the pretreatment of aqueous radioactive effluents, their performance in such applications remains not yet fully understood, particularly with regard to their interactions with diverse radioactive waste types.^31^ Moreover, a review of the literature has identified a continued need to develop mineral matrices based on alternative cements for the solidification of low- and intermediate-level radioactive waste (LLW and ILW).^32^

A recent review addressed the solidification performance and mechanism of typical radioactive nuclear waste in the context of geopolymer and geopolymer ceramic utilization. The authors underscored the significance of investigating additive incorporation to enhance the mechanical strength and stability of geopolymers and delving more profoundly into the use of alkali-activated materials in immobilizing challenging radionuclides, such as ^137^Cs, renowned for its mobility and potential for leaching.^29^

In response to the aforementioned gaps, this study aimed to develop a geopolymeric material from the alkaline activation of ash produced from calcined sewage sludge, specifically for immobilizing LLW and ILW, including wet and solid forms contaminated with ^137^Cs. The sludge was collected from three wastewater treatment plants in São Paulo, Brazil, with the objective of assessing the variability of the raw material. The research was conducted in three stages: (i) preparation and characterization of sewage sludge ash, including sludge collection and calcination; (ii) optimization of geopolymer formulation using factorial design and empirical mathematical modeling; and (iii) testing the immobilization performance on activated carbon (AC) and IERs contaminated with ^137^Cs. To our knowledge, this study uniquely investigates the influence of key geopolymer synthesis variables through experimental design, response surface methodologies, and includes SSA from multiple sources for radioactive waste treatment—an area underrepresented in the literature.

## 2 Materials and Methods

Initially, a series of tests were carried out in order to verify the technical feasibility of using the ash produced by the sludge from the three wastewater treatment plants (WWTPs). The preparation of geopolymers from SSA was carried out through a series of experiments considering the origin of the sludge dewatered from the sewage treatment plants, chemicals added to the geopolymeric process, temperature: 80–380 °C and drying time: 2–24 h (refer to Supporting Information, Table S1, for the experimental conditions). In addition to the parameters of the geopolymer generation process, these same parameters were considered in the preparation of the ash via calcination, with temperature and calcination time of 350–950 °C and 2–6 h, respectively.

In addition to these variables, other factors were investigated such as (i) initial curing temperature: 40–60 °C; (ii) initial curing time: 2–6 h; (iii) apparent mechanical strength; (iv) resistance to immersion in water for 24 h; (v) pH of the resulting mixture (alkaline according to the literature); (vi) heat release during the mixing processes with NaOH solutions in different concentrations (8, 12 and 14 M) (refer to Supporting Information, Table S2). The technique used at this stage was the one-factor-at-a-time (OFAT), based on applications already successful in the literature, but using experimental conditions so far not yet fully explored. Figure S2 shows some photos taken during this first phase of the study.

After confirming the technical feasibility of the sludge through the monothetic analysis, the work was divided into three phases: (1) collection, analysis and preparation of sewage sludge; (2) feasibility of using geopolymers obtained from the combination of MK as the main precursor and the addition of SSA to improve their properties; (3) tests aimed at real application in radioactive waste, with cesium as the selected model element.

In this section, we first describe the materials and methods used in each of these stages. These descriptions include the first stage: collection of sewage sludge from three SABESP domestic wastewater treatment plants in the state of São Paulo, followed by sludge sampling, ash preparation, and tests with the geopolymer with 100% SSA. The details regarding the methodologies employed for the sampling of sewage sludge, the preparation of SSA, and the characterization of the latter, in addition to the experimental design, can be found in the Supporting Information, Text S1. Then, we describe the second step of the work, with studies on the feasibility of using the tailored geopolymers, exploring the combination of MK as the main precursor and the addition of SSA to improve its properties. Details of the third step are also given, including tests on the selected geopolymers, focusing on real applications in radioactive waste management, with the two materials tested, AC and IERs, both contaminated with ^137^Cs. Finally, we give the details of the comprehensive analysis made throughout this study, since the collected materials from SSA, geopolymers prepared with different precursors and experimental conditions, as well as the tests with the simulated radioactive wastes.

### 2.1 Preparation of Standard Specimens Only with MK without and with the Addition of SSA

For the standard geopolymer, without SSA addition, the following materials were used: MK, sand, 8 M activating solution (AS8M): AS composition: sodium hydroxide (NaOH) + neutral sodium silicate (Na_2_SiO_3_), water, and calcium hydroxide (CaOH).

The procedure used to produce this geopolymer was: (i) weighing the solid ingredients and mixing with a focus on homogenization; (ii) adding water; (iii) adding the activating solution and using a mortar mixer to prepare the geopolymer; (iv) molding the 5 × 10 cm specimens. The accelerated curing process was used in an oven for 6 h at 60 °C. The specimens then remained at room temperature for a further 15 days to complete curing and were then subjected to axial compressive strength tests. For the geopolymer incorporating SSA, the following raw materials were used: MK, SSA, sand, and AS8M. SSA ratios of 10%, 30%, 50% and 70% were used to replace MK.

### 2.2 Tests with Simulated Radioactive Waste

#### 2.2.1 Leaching Tests

Leaching tests were carried out in accordance with ASTM C1318-21^33^ to investigate the leaching behavior of cesium in solidified geopolymer samples. The leaching tests were carried out in polypropylene containers containing 100 mL of deionized water as a leaching agent, keeping the specimen immersed for up to 11 days. The experiments were carried out in duplicate. Cesium nitrate (CsNO_3_) with a purity of 99.8% (Merck, Germany) was used.

The geopolymer specimens, with a diameter of 2.5 cm and a height of 1 cm, were prepared with 1.11 g of MK, in accordance with standard NBR-15894-1:2010.^1,34^ 16 g of sodium silicate activator solution with 0.10 g of 8 M NaOH, 0.10 of CsNO_3_, 0.51 g of tap water, 3.33 g of standard sand, according to NBR 7214:2015.^35^ In addition, 0.13 g of Ca(OH)_2_ and SSA were also added. The preparation conditions are listed in Table S4. The chemicals used were of technical quality. The samples were produced in duplicate and named as Standard, SSA—Barueri 10%, SSA—Barueri 30%, SSA—Bragança Paulista 10%, and SSA—Bragança Paulista 30%.

Regarding the initial cesium activity, its theoretical initial activity value was 340 Bq (refer to Supporting Information, Text S1). The experimental measurement, made on a high-purity germanium detector, resulted in a value of 330 Bq (average value of the analysis replicates) after a counting time of 1 h, indicating that the experimental measurement was close to the theoretical one.

The geopolymer samples were prepared by homogenizing them with a 12.7 g L^–1^ solution of CsNO_3_ and adjusting the pH to 7. A solution of ^137^Cs (330 Bq) was added to this solution. The geopolymer specimens were placed in leaching containers with 100 mL of deionized water, so that all sides of the specimens were in contact with the leachant. The leachate was replaced with distilled water after contact times of 2 h, 7 h, 1 day and then daily until the 11th day (for the scheme of the leaching tests and mathematical equations, refer to Supporting Information, Figure S5 and Text S2, respectively).

### 2.3 Analysis

#### 2.3.1 ICP

The aim of these analyses was to determine the elemental composition of sewage sludge and determine whether it could be used as a raw material to produce a geopolymer, since it is essential that the elements Al, Si, Ca, Na, O and Fe are present. The samples were subjected to a chemical dissolution process with hydrogen peroxide and hydrochloric and nitric acids, until the organic compounds were completely removed. The analysis was performed using a PerkinElmer ICP–OES, model Optima 7000 DV. ICP–OES analyses were carried out on the raw sludge in order to identify and verify its elemental structure and content, and to identify possible difficulties related to the manufacturing of the geopolymers by using ash from different sewage sludges.

#### 2.3.2 XRF and X-ray Analysis

X-ray fluorescence (XRF) was used to characterize the ash and also the prepared geopolymers. The equipment was a WDXRF spectrometer (model RIX 3000, Rigaku Co, Tokyo, Japan). For the analysis, pellets were prepared from samples that had previously been crushed, dried in an oven at 105 °C and sieved to obtain particles with a diameter of less than 0.065 mm.

The sludge ash samples and geopolymers were also analyzed by X-ray diffraction analysis. The analyses were carried out using a XRD diffractometer (Multiflex model, Rigaku Co, Tokyo, Japan). All the samples were analyzed using Cu Kα radiation at 800 W, in the 2θ range from 3 to 70° with a step size of 0.02° and 8 s per step. The results were compared with the reference diffraction in standard database files using Bruker’s Diffrac EVA software version 3.1 (qualitative analysis), using the PDF2-2003 database. For more details on XRD analysis and libraries used, refer to Supporting Information, Text S4.

#### 2.3.3 Scanning Electron Microscopy and Energy Dispersive X-ray Spectrometer

A JSM-IT700HR ultrahigh vacuum field emission SEM (SEM-FEG) was used for high-resolution, high-quality image mapping of nano- and microstructures. It has the following specifications: secondary electron imaging (SEI) resolution: 1.0 nm (with an accelerating voltage of 15 kV), 2.2 nm (with an accelerating voltage of 1 kV), accelerating voltage: 0.5 to 30 kV, beam current: in the order of 10^–13^ to 2 × 10^–9^ A, with magnifications of around 650,000 times and a resolution of 1.0 nm. The EDS was a Thermo Scientific EDS Noran System Six, which allows the elemental composition of the samples to be obtained.

#### 2.3.4 Gamma Spectrometry

The samples were analyzed nondestructively by gamma-ray spectrometry using a Canberra detector with high-purity germanium crystal (HPGe). The HPGe detector has a high resolution for discriminating γ radiation energy, providing lower uncertainty. The detector is coupled to conventional electronics using the Genie-2000 program, which uses a multichannel analyzer installed on a computer, which identifies the unique characteristic peaks of each gamma-emitting radionuclide in the spectrum and, using the program’s extensive library, it is possible to identify and quantify each radionuclide, in this study ^137^Cs.

### 2.4 Preparation of Sewage Sludge-Only Specimens and Compressive Strength Tests

The procedure used to produce geopolymer with SSA only was: (i) weighing the solid ingredients and mixing the solid ingredients with emphasis on homogenization; (ii) adding water; (iii) adding AS (AS8M) and using a mortar mixer to prepare the geopolymer; (iv) molding the 5 × 10 cm specimens. The accelerated curing process was used in an oven for 6 h at 60 °C. The specimens then remained at room temperature for a further 15 days to complete curing and were then subjected to axial compressive strength tests.

The equipment used to carry out the compressive strength tests was a manual hydraulic press (Solotest brand) with a digital indicator for compression or bending tests. This equipment has a capacity of 20 tf and a resolution of 0.01 tf. It has an upper plate with a ball joint for uniform load distribution and comes with a pedestal with a height suitable for breaking cylindrical CPs (*Ø* 5 × 10 cm).

## 3 Results and Discussion

### 3.1 Analysis of the Sewage Sludge

#### 3.1.1 ICP and X-ray Fluorescence

The results of the elemental analysis by ICP–OES are shown in Table S5. It can be seen that the concentrations of the elements differ considerably in the three treatment plants. This is due to the type of sewage each treatment plant treats and the treatment process. These analyses were important for obtaining essential information for producing a geopolymer, i.e. having an approximate value for the concentration of aluminum (Al) and also checking for the presence of possible interferents.

It can also be seen that Al, Ca and Fe are the most abundant elements in the ash. The most important of these is aluminum, which together with Si and/or P can form a geopolymer in the presence of alkalis. Ca and Fe, on the other hand, can negatively interfere in this process. All these elements are found in the form of oxides and their contents are shown in Table S6.

It can be seen that the ash from the PNM has the lowest levels of Al and Si oxides and the highest levels of calcium oxide (CaO). These levels may be an indication of the unfeasibility of using this material in the production of geopolymer. The presence of Mg and Ca can also affect the hardening time in the production of this material.^36^ Thus, given the MgO and CaO contents observed in the ashes from the three WWTPs, we observed a very fast initial setting time of around 30 s, which was detrimental to its handling.

By correcting these contents, it was possible to improve workability by increasing the setting time by approximately 1 h. Sulfur (S), whose role in the geopolymer is not yet fully understood, is present in all three SSA. However, only BA ash had a content slightly higher than 5%, indicating that the variations observed in the value of this parameter may be of little relevance to the possible differences observed in the test results. It is important to note that sulfur oxide can be a compound that interferes with the long-term durability of geopolymers. There are no reports in the literature that directly relate SO_3_ to geopolymers,^37^ however, taking into account the pozzolanic characteristics, the similarity to the properties of Portland cement and the group of zeolite minerals, the formation of ettringite should be considered, as occurs in hydrated cement in the presence of sulfates. In this context, the presence of S could possibly form ettringite, as occurs in hydrated cement in the presence of sulfates.^24^

In the case of cement, the sulfate content must not exceed 5% so that there is no excessive formation of ettringite,^38^ which can damage the hydrated cement in the long term. Of the elements analyzed, Si and Al are the ones that form the geopolymer, along with oxygen (O), and there is a minimum ratio between them necessary for the reaction to occur properly. The Si/Al ratio should always be equal to or greater than one.^39^

These elements are found in the form of oxides (Table S6) and, in this case, the molar ratio that should be recommended between SiO_2_/Al_2_O_3_ is 3.5–4.5.^3^ However, just knowing the quantities of these elements or oxides is not enough, as alkaline activation will not take place if there is no energy available. The degree of crystallinity in which these elements are found will define this. The lower the degree, the greater the chance of success in obtaining geopolymers. Hence the need to characterize the material using the X-ray diffraction technique.

#### 3.1.2 X-ray Diffraction

Table 1 shows the crystalline phases and its contents obtained with the SSA prepared from the three WWTPs (for the diffractograms, refer to Supporting Information, Figure S5).

**Table 1:** Crystalline Phases Identified in SSA from BA, PNM and BP by the Rietveld Method

crystalline phases	content (%)
BA	
corundum (Al_2_O_3_)	4.5
anorthite (CaAl_2_Si_2_O_8_)	21.2
hematite (Fe_2_O_3_)	38.2
K_2_O	3.9
quartz-α (SiO_2_)	32.2
PNM	
quartz-α (SiO_2_)	19.4
Ca-olivine (Ca_2_SiO_4_)	14.7
Mg Fe O	6.0
sodium silicate (Na_2_SiO_3_)	4.9
berlinite (AlPO_4_)	8.7
arinite (Ca_21_Mg[(Si_0.75_Al_0.25_)O_4_]_8_O_4_Cl_2_)	30.2
Ca Na Mg PO_4_	16.2
BP	
quartz-α (SiO_2_)	64.3
hematite (Fe_2_O_3_)	20.8
adularia (KALSi_3_O_8_)	9.6
Mg Ti O	5.3

The principal distinctions between the BA, PNM, and BP samples are discernible in their crystalline phase compositions and degrees of crystallinity, which directly influence their suitability for geopolymer production. The Rietveld method was employed to analyze the diffractograms, which revealed that the degrees of crystallinity of BA, PNM, and BP were 15%, 95.5%, and 37%, respectively. The high crystallinity and significant amounts of arinite (30.2%) and Ca-olivine (14.7%) present in PNM render it unsuitable for geopolymerization. Its highly crystalline structure lacks the requisite amorphous characteristics to initiate the geopolymerization reaction. An amorphous phase is of paramount importance, as its structural disorder allows the requisite chemical reactions to occur, thereby facilitating the formation of geopolymers. Therefore, PNM was excluded from the geopolymer manufacturing tests.

In contrast, BA, with a low degree of crystallinity (15%), contains significant quantities of anorthite (21.2%), corundum (4.5%), and hematite (38.2%). This composition provides a substantial source of aluminum and iron, which can contribute to the thermal stability and durability of geopolymers. The BP sample, which has a high quartz-α content (64.3%) and moderate crystallinity (37%), may require additional activation to increase early reactivity. However, it offers promising mechanical strength and stability upon curing. Overall, these findings suggest that only BA and BP possess the necessary characteristics for geopolymer production, enabling tailored formulations based on the desired curing rate, mechanical strength, or durability of the final material.

The application of the Rietveld method showed acceptable values for the statistical refinement indicators (GOF and *R*_wp_) (refer to Supporting Information, Table S7) due to the sludge ash having crystalline phases in its composition and with different concentrations. In addition, a high *R*_P_ was observed with the other techniques applied in the refinement carried out using the TOPAS and EVA programs, particularly in the detection of the constituents most present in the samples, such as quartz-α (SiO_2_), allinite, hematite, and clay minerals.

The molar ratios between the two compounds were calculated using the SiO_2_ and Al_2_O_3_ contents obtained from the XRF analysis (calculations based on a 100 g sample), as well as the crystallinity percentages (see Supporting Information, Table S8). It can be observed that both the SSA from Barueri and Bragança Paulista exhibit ratios that are lower than those proposed in the existing literature, which range between 3.3 and 4.5.^3^ Consequently, it was determined that a minimum of 12 and 20 g of SiO_2_ would be required to be added to the SSA from BA and BP, respectively, for the reaction with 100 g of SSA to occur.

#### 3.1.3 Axial Compressive Strength of Geopolymer with SSA Only

**Figure 1:**
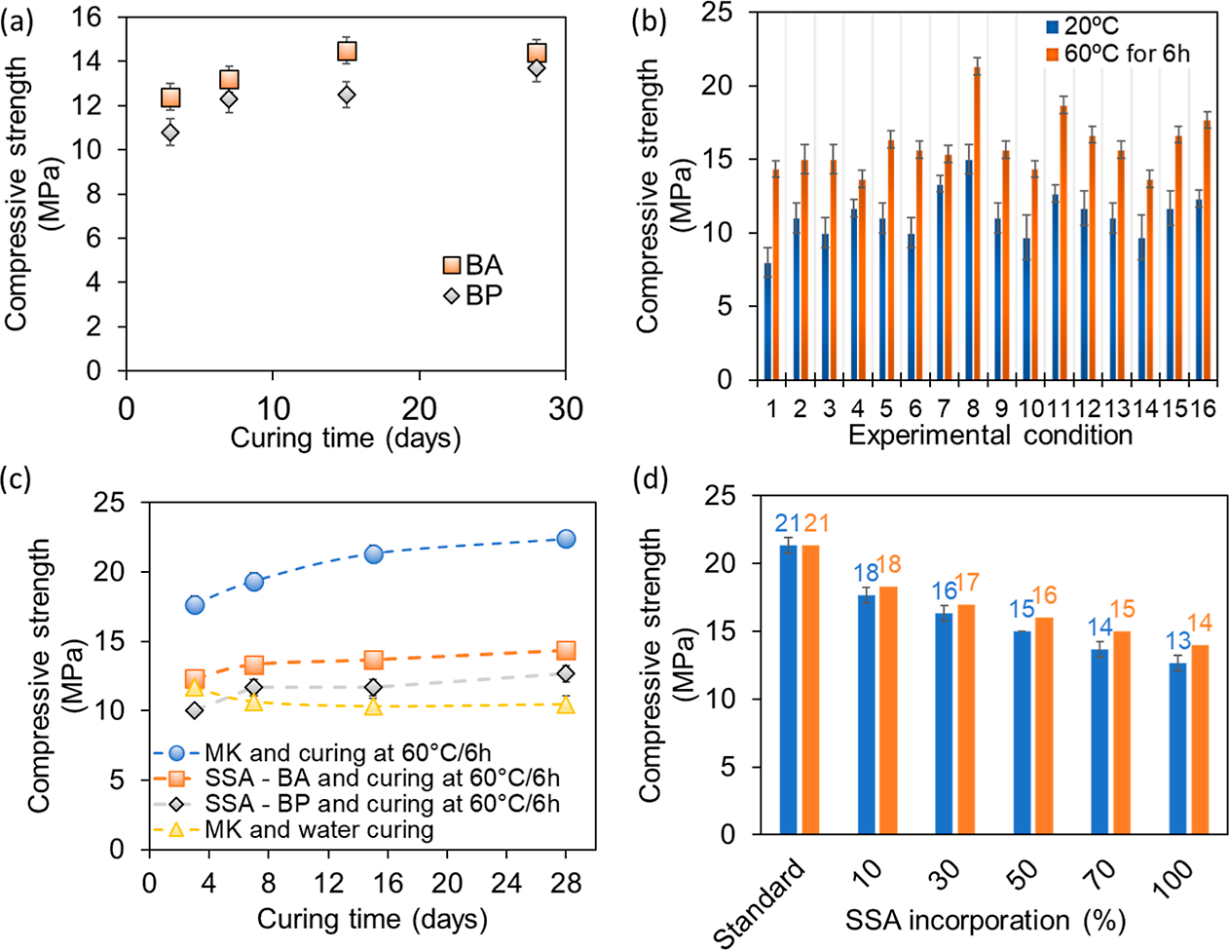
(a) Axial compressive strength values for different curing times with BA and BP SSA materials. Initial cure at 60 °C for 6 h; (b) axial compressive strength values for the each experimental conditions used; (c) evolution of axial compressive strength as a function of time for MK and curing at 60 °C for 6 h, SSA—BA and curing at 60 °C for 6 h, SSA—BP at 60 °C for 6 h, and MK and water curing; (d) study of different proportions of SSA in the production of geopolymer and its final compressive strength. *Mean ± standard deviation.

Figure 1a displays the results of the compressive strength of the samples prepared with BA and BP ashes during the exploratory stage (refer to Supporting Information for the exact values of the compressive strength, Table S9).

These results were instrumental in the subsequent stages, notably in the synthesis of the geopolymerization process. After only 3 days of curing, the minimum strength achieved was 12 MPa for BA and 10 MPa for BP, reaching a maximum of 13–14 MPa for both samples after 15 days of curing. It is noteworthy that, despite both materials reaching similar values after 28 days of curing, BA exhibited a higher axial compressive strength for all curing periods, reaching a stable value after 15 days. In contrast, BP demonstrated a slower rate of increase, reaching a stable value after 28 days. Given that a slightly higher value was obtained with the addition of SSAs compared to the 10 MPa established in regulations for wastes immobilized in cement, it was decided to include other materials in the formulation to improve the quality of the geopolymer but without SSAs in its composition. Among these materials, MK was used to increase the amorphous phase of the raw material. Calcium hydroxide, sand and water were also added to improve the workability of the geopolymer.

#### 3.1.4 Fractional Factorial Design

It is not clear from the specialized literature how different experimental conditions affect geopolymer formatting in relation to its ability to resist axial compression. To this end, five parameters were selected and evaluated according to the response variables *Y*_1_ and *Y*_2_, both relating to axial compressive strength (MPa), but for different initial curing temperature conditions (refer to Supporting Information, Table S10). For these tests, the method described for the material named “standard geopolymer” was used. Figure 1b presents the variations in compressive strength for different experimental conditions and curing. The best experimental conditions were achieved from experimental conditions CTA-8 (curing at room temperature, unit: g): *X*_1_ (mass of MK) = 120, *X*_2_ (mass of AS) = 125, *X*_3_ (mass of sand) = 360, *X*_4_ (mass of water) = 55, *X*_5_ (mass of lime) = 14.5; and CT60-8 (curing starting at 60 °C for 6 h, unit: g): *X*_1_ = 120, *X*_2_ = 125, *X*_3_ = 360, *X*_4_ = 55, *X*_5_ = 14.5. In other words, experimental condition N8 (CTA-8, CT60-8) was the best for both initial curing processes. However, the values achieved were significantly different, with 15 ± 1 and 21.33 ± 0.58 MPa, for curing at room temperature and curing starting at 60 °C for 6 h, respectively. This is a clear indication that the initial curing temperature plays an important role in the axial compressive strength after 15 days of total curing.

Accelerated curing is used because the temperature acts chemically, compensating for the effect caused by the low temperature and aiding the interaction of the activator solution in the polymerization reaction, since geopolymerization is an exothermic reaction. The lowest values were obtained for the experimental conditions CTA-1 (curing at room temperature, unit: g): *X*_1_ = 100, *X*_2_ = 90, *X*_3_ = 120, *X*_4_ = 25, *X*_5_ = 20 and CT60-4 (curing starting at 60 °C for 6 h, unit: g): *X*_1_ = 120, *X*_2_ = 125, *X*_3_ = 120, *X*_4_ = 25, *X*_5_ = 20; g; curing starting at 60 °C for 6 h. The equations generated from the application of this experimental design are presented in the Supporting Information, Text S5, for each curing condition.

**Figure 2:**
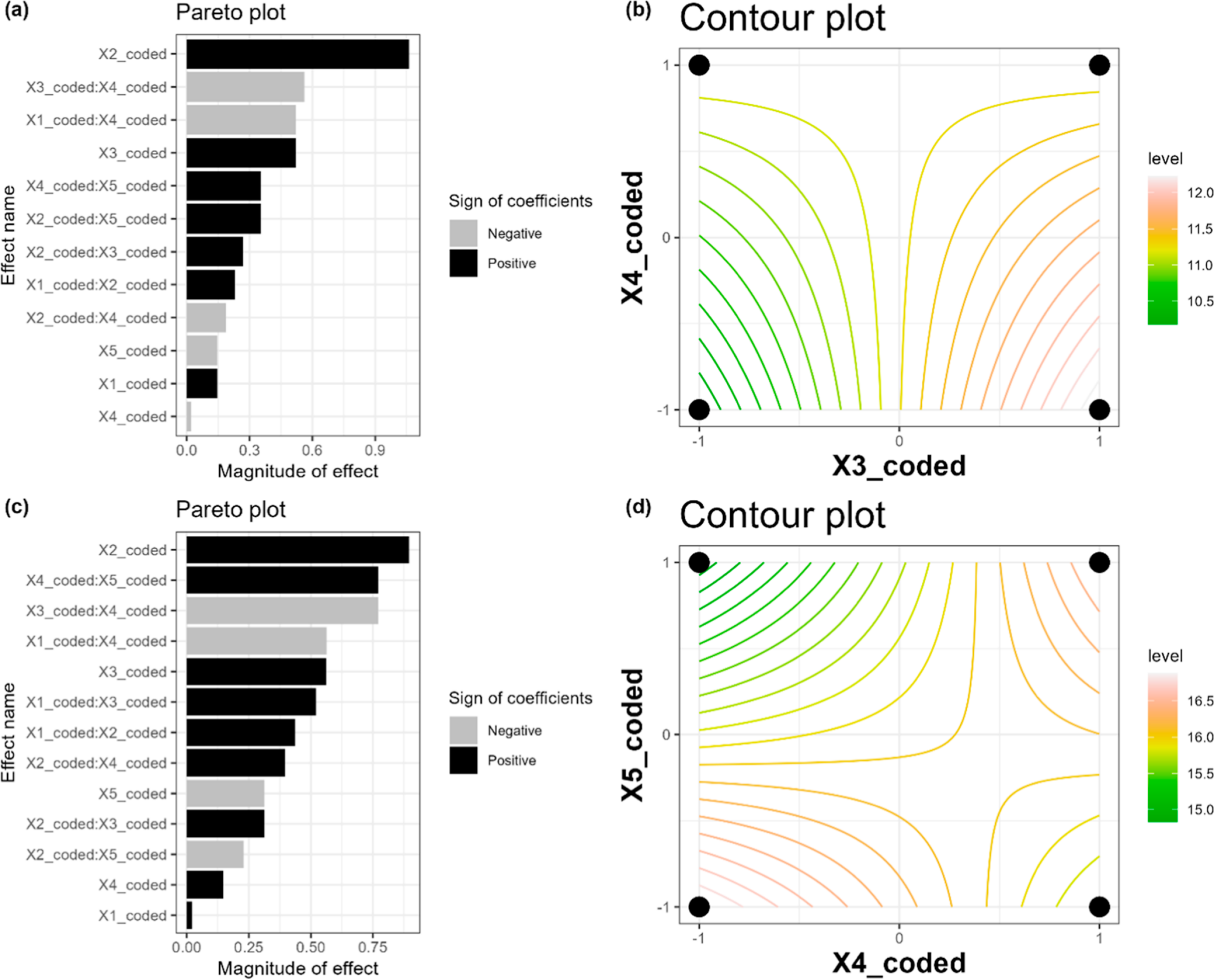
Pareto charts and contour curves for geopolymer fabrication from five different dependent variables. (a,b) Cured at room temperature; (c,d) with the beginning of curing at 60 °C/6 h. *X*_1_ = mass of MK, *X*_2_ = mass of AS, *X*_3_ = mass of sand, *X*_4_ = mass of water, *X*_5_ = mass of lime. Curing time = 15 days.

The correlation of determination (*R*^2^) obtained from the application of multiple linear modeling, which were 0.95 and 0.96 for the curing condition at room temperature and at 60 °C/6 h, respectively (see Supporting Information, Table S11, for the summary statistics). In other words, eqs S1 and S2 are able to explain 95% and 96% of geopolymer formation under different experimental conditions. To help understand the results, Pareto and contour curve graphs were generated, as shown in Figure 2.

It can be observed that for both initial curing processes, the most significant effect is *X*_2_ (AS, g), which has a positive impact on the enhancement of axial compressive strength. In other words, an increase in the mass of AS results in a proportional increase in the resistance of the geopolymer. The second most significant effect was the interaction between variables *X*_3_ (mass of sand) and *X*_4_ (mass of water) when the curing process was conducted at room temperature, which had a detrimental impact on strength, and *X*_4_ (mass of water) and *X*_5_ (mass of lime) when the curing process was conducted at 60 °C/6 h, which had a beneficial effect on strength. Figure 2b,d illustrates the contour curves generated from the regression model, which demonstrate that to enhance the resistance of the geopolymer, it is optimal to increase the amount of *X*_3_ and decrease the amount of *X*_4_ when conducting the initial cure at room temperature. Conversely, when the initial cure is conducted at room temperature, it is optimal to decrease both amounts of *X*_4_ and *X*_5_. It should be noted that when the variables are considered separately, an initial cure at room temperature results in a positive effect for *X*_3_ and a negative effect for *X*_4_. Conversely, an initial cure at 60 °C/6 h yields a positive effect for *X*_4_ and a negative effect for *X*_5_.

These findings indicate that the addition of water to the mixture during the curing process at room temperature should be kept to a minimum to prevent any adverse effects on the final strength of the material. However, it should be sufficient to allow the mixture to work effectively. Interestingly, the addition of water has a positive effect on the strength of the material when the initial curing process occurs at 60 °C/6 h. However, an examination of Figure 1b reveals a notable discrepancy between the outcomes of the experiments conducted at varying initial temperatures. To enhance the geopolymer’s resistance to axial compression, it is recommended to employ a higher initial temperature during the curing process and utilize a greater quantity of AS.

#### 3.1.5 Study of the Addition of SSA

After establishing the best experimental conditions (CT60-8: *X*_1_ = 120, *X*_2_ = 125, *X*_3_ = 360, *X*_4_ = 55, *X*_5_ = 14.5; g; curing starting at 60 °C for 6 h), the addition of SSA was tested, as well as the evolution of the axial compressive strength between 3 and 28 days.

Figure 1c illustrates the strength development of geopolymers with MK or SSA as the primary material, demonstrating clear distinctions in behavior based on the curing method. In the case of MK-based samples, the strength exhibited a consistent increase over the course of the curing period, reaching a peak at 28 days. In contrast, samples based on SSA exhibit minimal strength gains after 7 days, indicating that this may be the optimal curing time for these materials. A comparison of the final strengths indicates that the addition of SSA tends to result in a reduction in the strength of the geopolymer relative to that of MK. All samples were subjected to a curing process at 60 °C for a period of 6 h, followed by a subsequent phase of room temperature and water curing. This resulted in the attainment of strengths exceeding 10 MPa, which is in accordance with the prevailing national standards.

It is noteworthy that MK-only samples demonstrate substantially higher axial compressive strength, achieving regulatory requirements from the outset (see Supporting Information, Table S12, for values). The curing conditions have a significant impact on the strength of the samples. The samples that were cured at 60 °C for 6 h demonstrated superior performance compared to the samples that were cured in water, which exhibited markedly lower resistance. The samples that were based on SSA also demonstrated inferior performance, particularly those that were based on SSA-BP. The materials that were used included MK and SSA from BA and BP sources. The standard MK-only samples were cured at both 60 °C for 6 h and in water for comparison. Since the addition of SSA reduced the final strength of the material, different proportions were tested, as shown in Figure 1d.

**Figure 3:**
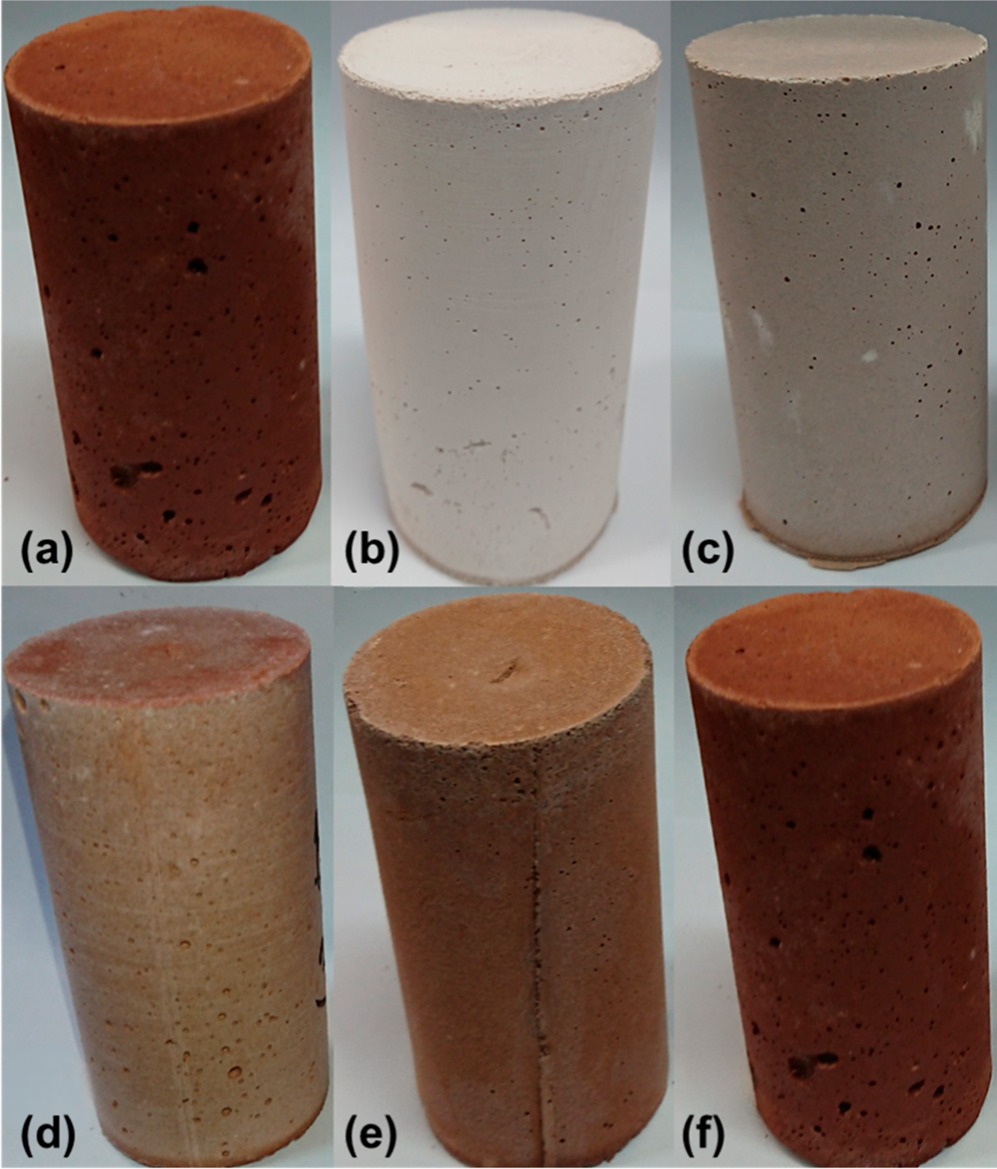
Geopolymer with the following specimens: (a) 100% SSA, (b) 100% MK, (c) 10% SSA, (d) 30% SSA, (e) 50% SSA, (f) 70% SSA.

The drop in strength is explained by the fact that the binder composed of MK and SSA requires a higher water/binder ratio, resulting in a decrease in cohesion between the particles. However, for a substitution of 10% and 100%, the decreases in mechanical strength values are approximately 15 and 34%, respectively, indicating that the use of SSA in materials for immobilizing radioactive waste can be viable from a technical point of view. This is especially important because reducing the mass of MK reduces the cost of the final product, given that the added SSA is an abundant and cheap product. With a view to producing a lower-cost product with good resistance to axial compression, adding 10% SSA to the mixture used in geopolymer production would be an attractive and viable alternative. It is important to note, however, that the cost of drying and calcining was not evaluated, nor was the yield of sewage sludge for ash. Despite this, it is known that sewage sludge is an abundant material and is disposed of in landfills, as well as having favorable chemical characteristics for the formation of geopolymers. Photographs of the geopolymer samples produced in accordance with the various SSA additions are presented in Figure 3.

**Figure 4:**
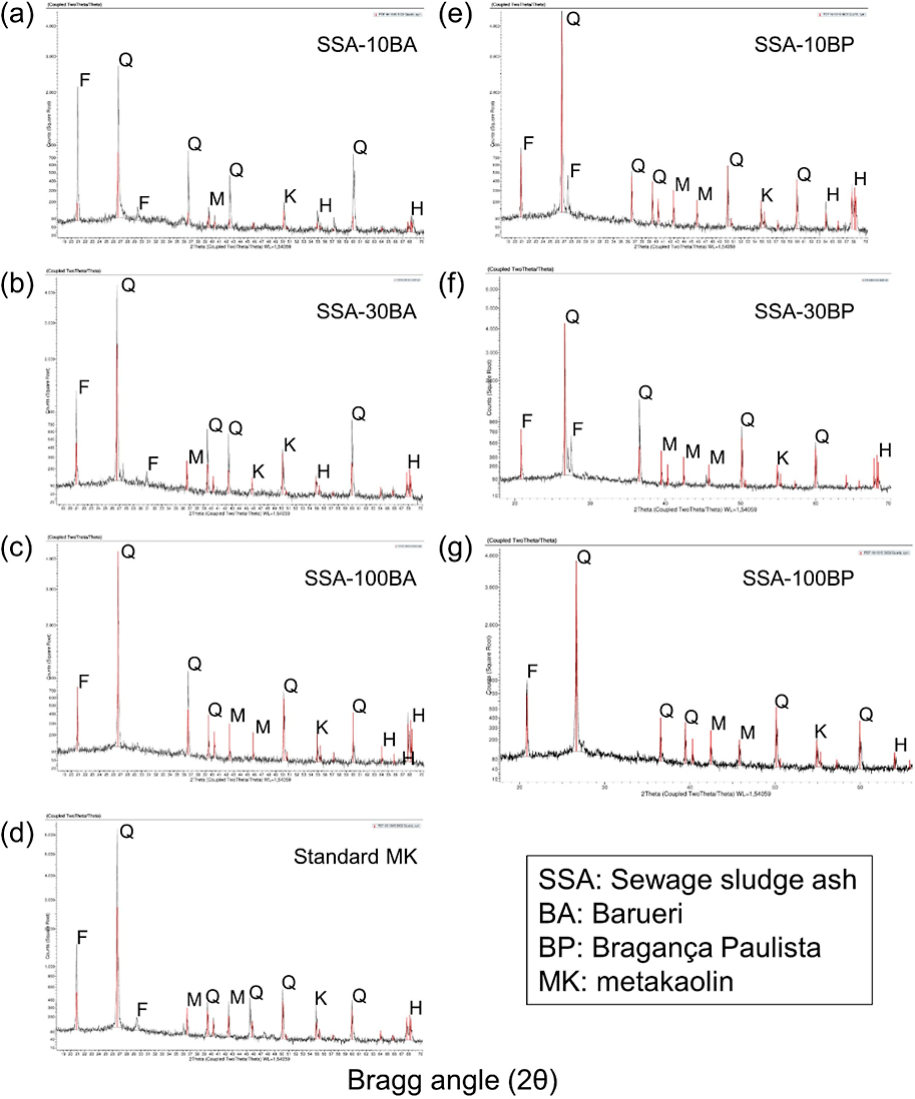
Diffractograms of geopolymers with the addition of SSA and MK-pattern. XRD standards for geopolymers with the addition of SSA: (a) SSA from Barueri with 10% MK, (b) SSA from Barueri with 30% MK, (c) SSA from Barueri with 100% MK, (d) standard MK, (e) SSA from Bragança Paulista with 10% MK, (f) SSA from Bragança Paulista with 30% MK, (g) SSA from Bragança Paulista with 100% MK. F: faujasite, Q: quartz, K: kaolinite, H: hematite, M: muscovite. All samples were analyzed at room temperature.

XRD studies were also carried out on standard MK geopolymer samples without the addition of SSA and with the addition of SSA (see Supporting Information, Figure S6, for the XRD analysis of each SSA). Different proportions of SSA were used to replace MK: 10% (SSA-10), 30% (SSA-30) and 100% (SSA-100). All the samples were compared with the standard and the respective degrees of crystallinity (see Supporting Information, Table S12). Figure 4 shows the formation of the crystalline phases.

The raw materials MK and SSA showed a variation in the baseline in the range of 12 to 32°, for both geopolymers with the addition of the ashes produced from B and BP, respectively, which is characteristic of the presence of an anamorphic phase. The crystalline phases found were: quartz (SiO_2_), kaolinite (Al_2_Si_2_O_5_(OH)_4_), muscovite (KAl_3_Si_3_O_10_(OH)_2_) and hematite (Fe_2_O_3_), found in the standard MK; quartz (SiO_2_), kaolinite (Al_2_Si_2_O_5_(OH)_4_, muscovite (KAl_3_Si_3_O_10_(OH)_2_, hematite (Fe_2_O_3_) and faujasite (Na_2_Al_2_Si_4_O_12_ 8H_2_O) found in the geopolymers with SSA. All the samples were cured at 60 °C for 6 h and then cured for 15 days at room temperature.

For the geopolymer pastes with SSA addition, all the samples presented a deviation line between 12 and 32°, which can be attributed to the amorphous phase of the geopolymer gels. The baseline for higher 2θ values compared to MK and SSA showed amorphous phases and can be attributed to the geopolymerization reaction as already observed in the literature.^40^

The presence of SSA influences the formation of a material with the characteristics of a zeolite, since a zeolite peak lower in intensity is observed after the curing period when compared to standard MK. The formation of the geopolymer gel and the material with the characteristics of a zeolite are directly related to the activity of the raw materials and the curing temperature.^4,41^

In a highly alkaline environment, with initial curing at high temperature, both alkalinity and temperature are considered important variables, playing important roles in the final outcome of the process. The alkaline nature of the environment directly influences the chemical and physical properties of the substance in question, while the high temperature during the initial curing phase has a significant impact on the formation and stability of the molecular structure. There is therefore an interdependence between the two. This situation favors the crystallization of aluminosilicate gels forming zeolite-type structures and the crystallization process is drastically reduced with an increase in SiO_2_/H_2_O.^42^

In this study, standard MK showed greater reactivity than SSA, so it was expected that geopolymers with greater amounts of it would show more intense zeolite formation and, consequently, greater compressive strength, which is what happened. Mortars produced from materials with a microporous-crystalline structure based on a 3D cage system of zeolites have lower compressive strength compared to the amorphous structure based on the 3D network of geopolymers based on aluminum silicate sources.^4^ Faujasite was also observed in SEM scans of broken geopolymer samples.

#### 3.1.6 Characterization of Geopolymers by SEM and EDS: Morphological and Compositional Analysis

The microstructures of the geopolymer pastes were studied by SEM analysis, which was carried out on the standard samples, SSA-10%, SSA30% and SSA100%, with the aim of evaluating the influence of SSA on the microstructure of the mortars that showed superior performance in terms of compressive strength. Figure S7–S17 show the microstructures of the geopolymers with initial curing at 60 °C/6 h and 15 days of curing. All the microstructures showed significant porosity, which may be due to the crystallization of the geopolymer gels and the subsequent formation of “Na P” type zeolites. Na P-type zeolites are identifiable by their “wool-ball” (WB) or “pine cone (PL) type” crystals. The regions represented by the letter *A* indicate the crystallization process. It can be seen that massive geopolymer gels make the transition to crystalline phases. Note that all the geopolymers synthesized had faujasite in their microstructure, which is a mineral group from the zeolite family of silicate minerals.

The geopolymerization potential of SSA was tested with sodium hydroxide activator solutions prepared at concentrations of 8, 10, 12, 14, and 16 M. The concentration that allowed the formation of the most resistant material was 12 M. In this case, the samples had an axial compressive strength close to 10 MPa, being the minimum acceptable according to standards. Compared to those found in the literature, this value is at least five times lower, demonstrating the need to improve the preparation method. Furthermore, it is possible that this result is only due to the solidification of the activator solution, since even though it did not have the ideal characteristics, the material produced with SSA from PNM also resulted in a product similar to the others, but without clear evidence that it is a real geopolymer.

The pore size and porosity of the geopolymer matrix is important because it relates to the size and quantity of the pores present in the material and can be related to long-term durability. Pores can facilitate the entry of liquid agents and contaminants, causing corrosion and weakening and consequently the durability of the structure.^43^ Previous investigations showed that the porosity of geopolymer cement has smaller pores and reduced capillarity when compared to the pores in the Portland cement matrix.^44^ This work corroborates previous studies,^45^ which pointed out that geopolymer matrices are similar in immobilizing heavy metals or radioactive waste. Among the selected ranges regarding grain diameters (see Supporting Information, Text S1—Preparation of the SSA), the optimal range was identified as 0.077–0.106 mm. Further insights into the role of this range on geopolymerization can be found in Supporting Information, Text S6.

### 3.2 Immobilization with Simulated Wastes and Characterization of Geopolymers with Radioactive Material

Table 2 shows the axial compressive strength results for the simulated wastes for the SSA addition values of 10, 30 and 50% (BA-SSA and BP-SSA). For the simulated wastes, AC and IER were used with 5%, 8% and 10% load incorporation in the immobilization matrix.

**Table 2:** Geopolymer with Different Materials Contaminated by Cesium^a^

material	SSA origin	saturated (MPa)			unsaturated (MPa)		
		5%	8%	10%	5%	8%	10%
		SSA 10%					
AC	BA	14.87 ± 0.6	12.38 ± 0.6	11.68 ± 0.6	14.37 ± 0.6	11.68 ± 0.6	10.58 ± 0.6
	BP	14.42 ± 0.6	12.53 ± 0.6	10.98 ± 0.6	13.68 ± 0.6	12.03 ± 0.6	10.23 ± 0.6
IER	BA	14.47 ± 0.6	11.98 ± 0.6	12.03 ± 0.6	14.08 ± 0.6	11.33 ± 0.6	10.83 ± 0.6
	BP	14.57 ± 0.6	11.93 ± 0.6	11.93 ± 0.6	13.93 ± 0.6	11.53 ± 0.6	10.53 ± 0.6
		SSA 30%					
AC	BA	14.32 ± 0.4	11.25 ± 0.4	10.87 ± 0.4	11.83 ± 0.4	10.11 ± 0.4	9.85 ± 0.4
	BP	13.68 ± 0.4	11.36 ± 0.4	10.91 ± 0.4	11.70 ± 0.4	10.26 ± 0.4	9.57 ± 0.4
IER	BA	13.35 ± 0.4	11.74 ± 0.4	10.65 ± 0.4	11.40 ± 0.4	10.49 ± 0.4	9.65 ± 0.4
	BP	13.25 ± 0.4	11.52 ± 0.4	10.33 ± 0.4	11.08 ± 0.4	10.69 ± 0.4	9.87 ± 0.4
		SSA 50%					
AC	BA	9.32 ± 0.4	9.25 ± 0.4	9.87 ± 0.4	8.83 ± 0.4	8.11 ± 0.4	7.85 ± 0.4
	BP	8.68 ± 0.4	9.36 ± 0.4	8.91 ± 0.4	7.70 ± 0.4	8.26 ± 0.4	7.57 ± 0.4
IER	BA	8.35 ± 0.4	8.74 ± 0.4	9.65 ± 0.4	8.40 ± 0.4	7.49 ± 0.4	6.65 ± 0.4
	BP	8.25 ± 0.4	8.52 ± 0.4	8.33 ± 0.4	7.08 ± 0.4	7.69 ± 0.4	6.87 ± 0.4

a The columns designated as 5%, 8%, and 10% indicate the quantity of activated carbon (AC) or ion exchange resin (IER) employed in the experiment.

It was necessary to saturate both AC and IER with calcium hydroxide for 24 h to correct the mass balance of the mixture, as the adsorption of the materials sequesters cations that are important for the geopolymerization reaction. This was observed by carrying out a series of OFAT experiments, in which the quality of the geopolymer was assessed by measuring its mechanical resistance to compression. It was observed that without saturation, the mechanical strength drops by around 3 MPa, due to the sequestration of cations.

**Figure 5:**
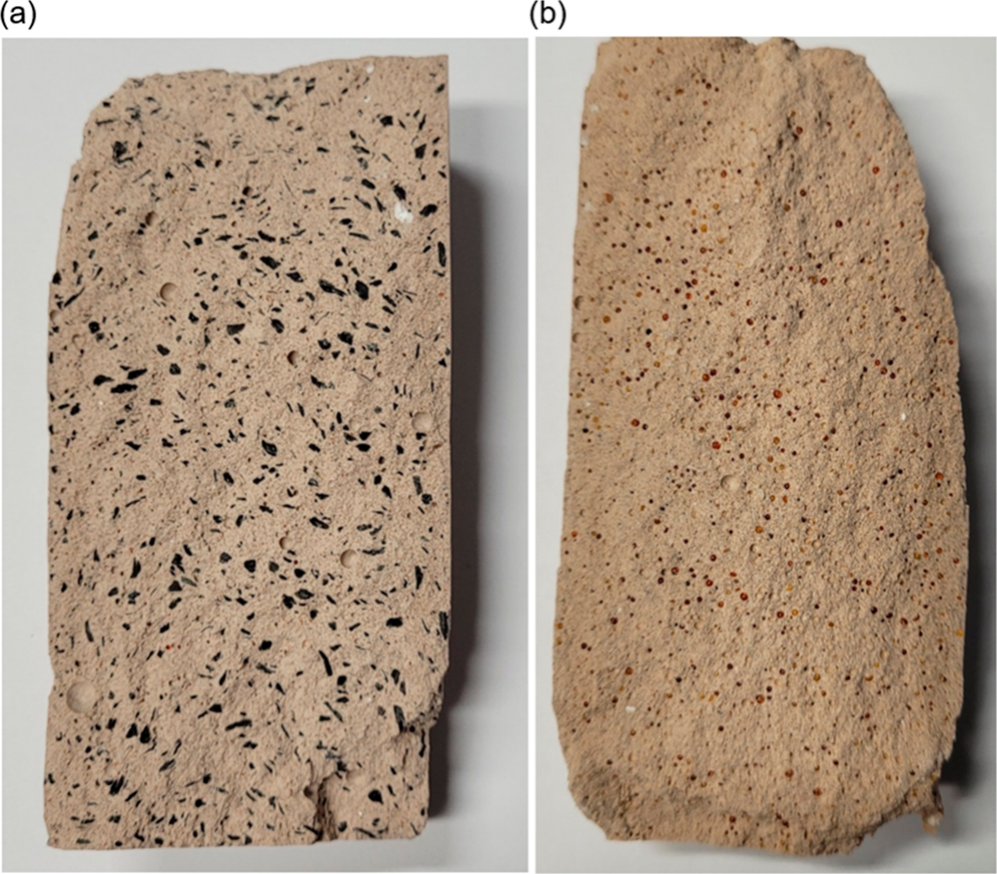
Geopolymer specimens with incorporation of simulated waste with (a) activated carbon; (b) ion exchange resin.

We can see a minimum of 10 MPa for BA and BP with a 10% load for saturated wastes: 10 MPa for BP with initial (accelerated) curing at 60 °C for 6 h and curing for 15 days at room temperature, and a maximum of 14 MPa for both under the same conditions. On the other hand, the samples with 50% added SSA did not show results higher than 10 MPa, leaving them below the recommended standard. Figure 5 displays the materials AC and IER immobilized by the geopolymers, indicating good homogeneity across the samples, with lower presence of the wastes close to their boarders.

It was possible to obtain about 15 MPa of compressive strength, complying with the national standards considering cement as the immobilizing agent, therefore it can be used as an immobilization matrix for wastes of low and medium activity levels. The diffractograms with simulated AC wastes and IER are shown in Figure S18. The degrees of crystallinity obtained were, for AC: 43.77% amorphous and 56.23% crystalline; IER: 37.67% amorphous and 62.33% crystalline. The choice between immobilizing simulated wastes with AC or IER requires a careful analysis of the properties of the geopolymers. XRD tests can reveal whether the incorporation of AC or IER influences the crystalline structure of the geopolymers, indicating whether these additions are compatible with the objectives of wastes immobilization. In addition, XRD analysis can provide information on the thermal stability of the geopolymers, helping to determine their compressive strength, which is important for immobilization applications and meeting the requirements established regulations.

SEM and EDS analyses were also carried out for the geopolymers with 10% and 30% added SSA-B, for immobilization with simulated wastes containing AC. The microstructures of the geopolymer pastes were studied for the standard samples, SSA-10%, and SSA-30%, from Barueri 5% saturated with AC and IER, as they were the ones with the highest compressive strength. The aim of this stage was to assess the influence of SSA on the microstructure of the mortars that showed superior performance in terms of compressive strength.

**Figure 6:**
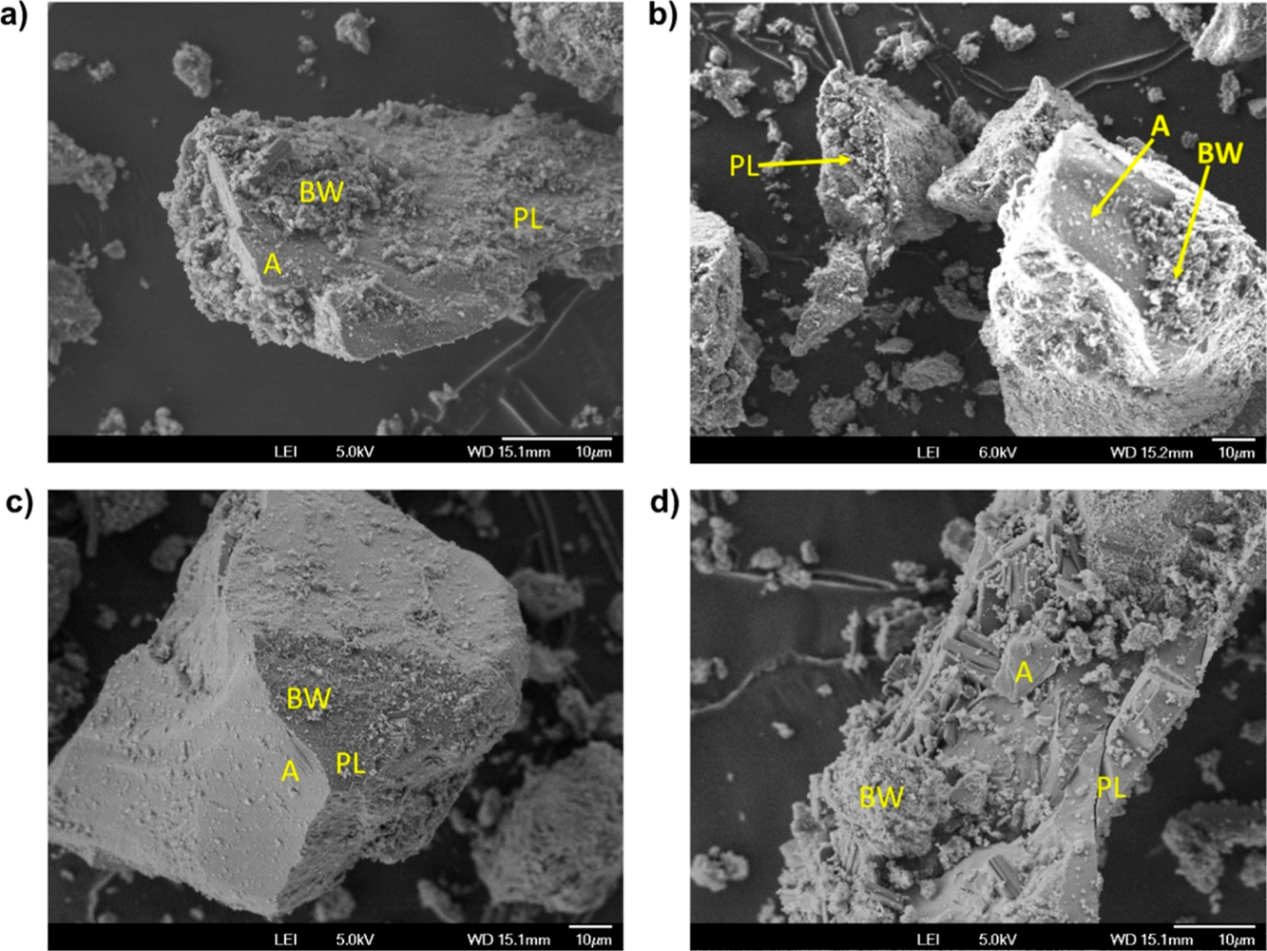
SEM for the geopolymers with (a) 10% addition of SSA—waste simulated with AC; (b) 30% addition of SSA—waste simulated with AC; (c) 10% addition of SSA—simulated waste with IER; (d) 30% addition of SSA—simulated waste with IER. *A, BW, PL: crystallization points.

Figure 6 shows the microstructures of the geopolymers with initial curing of 6 h at 60 °C and 15 days of curing. All the microstructures showed significant porosity, which may be due to the crystallization of the geopolymer gels and, subsequently, the formation of Na P-type zeolites. Na P-type zeolites are identifiable by their “ball-wool” (BW) or “pine cone (PL) type” crystals. The other regions represent the crystallization process where the massive geopolymer gels make the transition to crystalline phases. It is important to note that, like the geopolymers produced before immobilization, they also had faujasite in their microstructure.

Table 3 presents the elemental composition as determined by the EDS method. The data demonstrates significant variations in the elemental composition of geopolymer formulations with AC and IER, which were simulated as radioactive waste materials with varying additions of SSA. The principal discrepancies observed in the AC-based geopolymers pertain to the reduction in oxygen percentage (from 57 to 42%) and sodium percentage (from 12 to 7%) upon an increase in SSA from 10 to 30%. In contrast, IER-based geopolymers exhibit a high silicon content (over 58% with both 10% and 30% SSA), indicating a more stable, silicon-rich structure with fewer observable elemental variations. The increase in sodium and aluminum with higher SSA amounts is anticipated, given their significant concentrations, as illustrated in Table S5. This silicon-rich composition may facilitate cesium immobilization due to its chemical durability and resistance to leaching.

**Table 3:** Leaching Results in Relation to Retention and Efficiency for Each Sample Studied

Bq					
	*P*	SSA-B10	SSA-B30	SSA-BP10	SSA-B30
total initial activity	335 ± 1	312 ± 12	355 ± 15	305 ± 5	355 ± 15
2 h	9 ± 0	13 ± 0	17 ± 1	13 ± 2	17 ± 1
7 h	20 ± 0	24 ± 1	27 ± 1	25 ± 0	27 ± 1
1 day	17 ± 1	20 ± 1	21 ± 1	22 ± 2	21 ± 1
2 days	11 ± 1	11 ± 0	11 ± 1	11 ± 1	11 ± 1
3 days	11 ± 1	11 ± 1	11 ± 1	11 ± 0	11 ± 1
4 days	11 ± 1	11 ± 1	11 ± 1	11 ± 1	11 ± 1
5 days	11 ± 0	12 ± 1	11 ± 0	15 ± 0	11 ± 0
6 days	11 ± 1	10 ± 0	10 ± 0	14 ± 0	10 ± 0
7 days	8 ± 0	10 ± 0	11 ± 1	14 ± 0	11 ± 1
8 days	9 ± 0	10 ± 0	10 ± 0	11 ± 0	10 ± 0
9 days	9 ± 0	9 ± 0	11 ± 0	11 ± 0	11 ± 0
10 days	6 ± 1	8 ± 1	9 ± 0	10 ± 1	9 ± 0
11 days	4 ± 0	4 ± 1	3 ± 0	4 ± 1	3 ± 0
total leachate per sample	134 ± 1	150 ± 1	160 ± 1	170 ± 3	160 ± 1
retained in samples	201 ± 0	162 ± 11	195 ± 16	136 ± 8	195 ± 16
retention efficiency	60 ± 0	52 ± 2	55 ± 2	44 ± 2	55 ± 2
leaching rate (cm d^–1^)	2.55 × 10^–5^	2.85 × 10^–5^	3.05 × 10^–5^	3.23 × 10^–5^	3.25 × 10^–5^

#### 3.2.1 Leaching Tests with ^137^Cs

CNEN standard NN 6.09 also establishes other acceptance criteria for the disposal of low and medium-level radioactive waste, such as the leaching rate.^46^ In general, the leaching rate of the dominant radionuclides in the solidified product must be kept extremely low. This is especially important for beta and gamma emitters, where the release rate must be less than 0.005 cm day^–1^ over a cumulative period of 150 days at a constant temperature of 22 ± 4 °C. This strict restriction aims to minimize environmental contamination and protect public health.

The first two experimental values were excluded from analysis because the phenomenon of washout occurs before the samples enter a state of normality, i.e. the stationary phase. Washout, in leaching tests, refers to the process by which certain soluble or dissolved components of a sample are removed or washed out of the solid matrix during the initial phase of the leaching test. This occurs when water or another solvent is added to the solid sample to simulate leaching conditions, in which the goal is to extract the soluble components and determine their concentration in order to assess the environmental impact or retention effectiveness of a given material.

**Figure 7:**
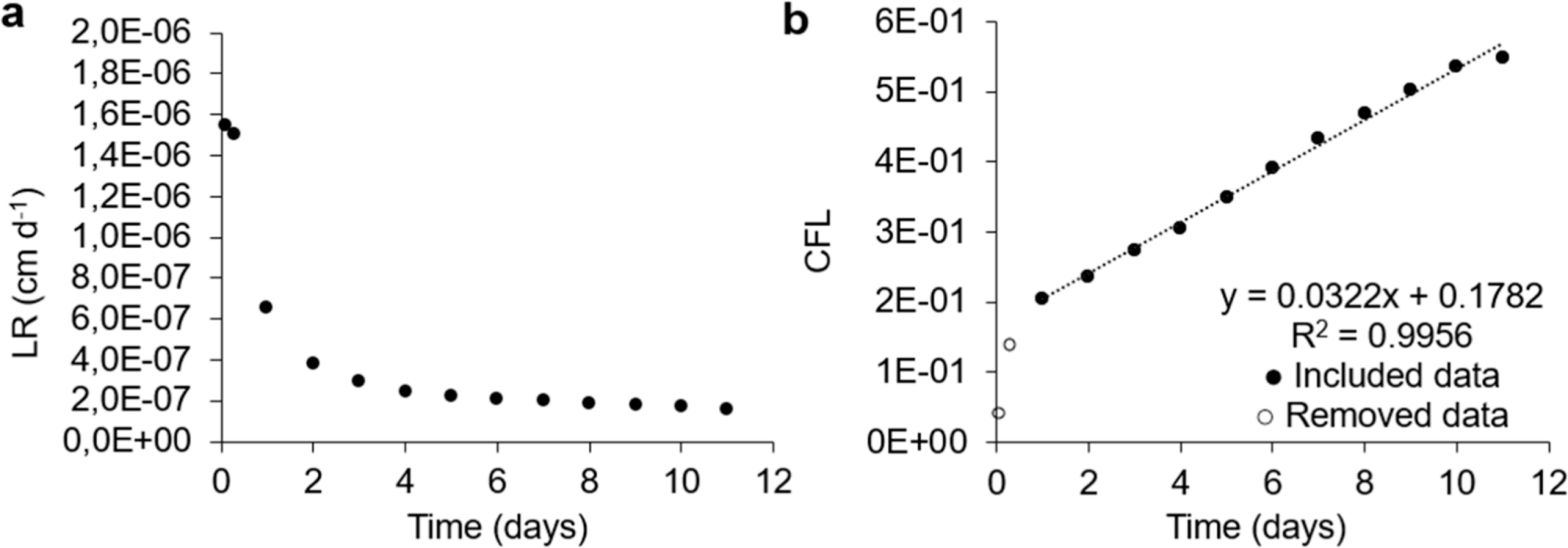
Geopolymer with the addition of 30% SSA from BA (a) leaching rate (LR) of cesium; (b) cumulative fraction of cesium leachate (CFL).

Table 3 lists the leaching results for the samples without the addition of SSA and for the samples with 10% and 30% additions of SSA-BA and SSA-BP. Figure 7 shows the leaching results when 30% of SSA from BA was added in the mixture (refer to Supporting Information for the other leaching study graphs). The cesium leaching rate in standard geopolymer showed a similar behavior in all geopolymer samples containing 10% and 30% SSA from BA and BP. The need to discard the two initial points of the washout curve was observed, as previously seen in the literature.^47^ The data revealed that the cesium leaching rates in all the samples were very close, indicating a consistent pattern in the geopolymers that received SSA additions, compared to the standard geopolymer, which did not receive such additions. This trend was also corroborated when analyzing the CFL in the three geopolymer variations mentioned above: standard, with 10% SSA, and with 30% SSA from BA and BP.

After the initial washing phase, characterized by an observation of rapid leaching of the cesium associated with the surface of the geopolymer samples, the LRs are predominantly under the control of the diffusion process in stationary mode, maintaining a constant rate over time. This phenomenon implies that the release of cesium from the geopolymer samples is governed mainly by molecular diffusion in the surrounding medium. When analyzing the LRs calculated for the 11th day of leaching, there is a variation in these values, ranging from 2.55 × 10^–5^ to 3.23 × 10^–5^ cm d^–1^. These figures represent the specific rates at which cesium is being released from the samples into the surrounding environment during this specific period of time.

In contrast, Abdelrahman et al.^48^ modeled the long-term leaching behavior of the radionuclides ^137^Cs, ^60^Co and ^152,154^Eu from cement-clay matrices. Three materials were studied, Portland cement, Portland-bentonite cement and Portland-red clay cement. The leaching rate was approximately 1.0 × 10^–2^ cm d^–1^ for the materials containing bentonite and red clay. From the authors’ graphs, it is not possible to know for sure the leaching rate obtained for Portland cement, but it is clear that there has been an increase in the rate for this material. According to the authors, the decrease in the leaching rate with the addition of bentonite and red clay to the cement grout was due to their low porosity, as well as the high sorption capacity of these clays. The Portland cement-bentonite matrix showed a pronounced increase in the resistance of all the radionuclides studied.

## 4 Conclusion

This study investigated the feasibility of using sewage sludge as a raw material for geopolymer production, employing a systematic approach from sludge collection to final product characterization. The collection from wastewater treatment plants was successful, ensuring a reliable raw material source. Preparation of sewage sludge ash was effective, with temperature and drying parameters influencing ash quality.

Characterization analyses revealed important physical, chemical, and mineralogical properties of the ash, essential for assessing geopolymerization potential. Specifically, the Si and Al content and degree of crystallinity were vital metrics. X-ray fluorescence and diffraction analyses indicated that ash from the Parque Novo Mundo WWTP was unsuitable for geopolymer production, whereas sludge from Barueri and Bragança Paulista showed promise, though requiring Si–Al ratio adjustments for optimal mechanical properties.

The geopolymer synthesis process proved viable, transforming waste into a potentially useful material. Key parameters such as activator solution concentration, curing conditions, and preparation methods were studied, revealing that an 8 M sodium hydroxide solution produced the strongest material. Accelerated curing and proper preparation, including prewetting the ash, enhanced the geopolymer’s properties.

A factorial experimental design optimized synthesis conditions, identifying influential factors and their interactions. This led to the development of an empirical model describing the relationship between process variables and geopolymer characteristics, facilitating quality prediction and control. Minimum water addition was important for workability, while higher initial curing temperatures and activator solution amounts improved mechanical properties.

Characterization of the produced geopolymer demonstrated its suitability for applications such as radioactive waste immobilization and sustainable building materials, confirming the material’s viability with simulated wastes. Thus, sewage sludge ash, with added metakaolin, can produce a geopolymer suitable for radioactive waste deposition.

In summary, this study advances scientific and technological knowledge in geopolymers, showcasing the potential of waste as a sustainable raw material. It promotes sustainable practices in the nuclear industry and beyond, benefiting both industry and society.

## Supporting Information Available

Text S1: Sampling and preparation of sewage sludge ash (SSA); feasibility of geopolymers with different precursors; factorial design methodology. Text S2: Calculation of cesium activity. Text S3: Leaching tests and parameters. Text S4: Analysis of crystalline phases. Text S5: Equations from the factorial experimental design. Text S6: Sieving and granulometry. Supplementary Tables Table S1: Experimental conditions for geopolymer synthesis. Table S2: Exploratory tests for geopolymer resistance. Table S3: Real and coded values in factorial design. Table S4: Geopolymer formulation details. Table S5: Elemental analysis of sewage sludge by ICP–OES. Table S6: X-ray fluorescence analysis of SSA. Table S7: R/DRX values and GOF from SSA analysis. Table S8: SiO_2_/Al_2_O_3_ molar ratios. Table S9: Compressive strength values over curing time. Table S10: Experiments from fractional factorial design. Table S11: Statistical summary of linear model fits. Table S12: Evolution of axial compressive strength. Table S13: Degree of crystallinity of geopolymers. Table S14: EDS analysis for geopolymers with SSA. Supplementary Figures Figure S1: Sludge dryer and gas scrubbing system. Figures S2 and S3: SSA preparation and calcination process. Figure S4: SSA samples from different WWTPs. Figure S5: Scheme of leaching tests. Figures S6–S18: Diffractograms, SEM, and EDS analyses of geopolymers with SSA and different precursors. Figures S19–S31: Leaching behavior of cesium in geopolymers with varying SSA additions. Supporting References: References for Supporting Information (PDF)
Raw data (XLSX)
