# Supplementary material for: Toward Sustainable Radioactive Waste Management: Geopolymerization of Sewage Sludge Ash as a Viable Solution
Source: ACS Omega. 2025 Feb 19;10(8):7683–96. doi: 10.1021/acsomega.4c07195 (PMC11886906; doi:10.1021/acsomega.4c07195)
Supplement: Supplementary file 1 — ao4c07195_si_001.pdf [file ao4c07195_si_001.pdf]

Supporting Information

Towards Sustainable Radioactive Waste Management: Geopolymerization of Sewage Sludge Ash as a Viable Solution

Alexandre Las Casas*, Leandro Goulart de Araujo†, Roberto Vicente, Júlio Takehiro Marumo

Nuclear and Energy Research Institute, IPEN-CNEN/SP, São Paulo, Av. Prof. Lineu Prestes, 2242

†Present Address: Université de Lyon, Université Claude Bernard Lyon 1, CNRS, IRCELYON UMR 5256, 69626 Villeurbanne Cedex, France

Corresponding author: Dr. Alexandre Las Casas

*E-mail address: alexandre.las.casas@alumni.usp.br

Abbreviations

AS: Activating Solution

BA: Barueri Sewage Plant

BP: Bragança Paulista Sewage Plant

IER: Ion Exchange Resin

MK: Metakaolin

PNM: Parque Novo Mundo Sewage Plant

SSA: Sewage Sludge Ash

WWTP: Wastewater Treatment Plant

Text S1

Sampling sewage sludge

Sludge was collected from three domestic sewage treatment plants run by the São Paulo Basic Sanitation Company (SABESP): (1) 500 kg of dewatered sludge from the Parque Novo Mundo (PNM) sewage treatment plant; (2) 500 kg of dewatered sludge from the Bragança Paulista (BP) sewage treatment plant (isolated system); (3) 500 kg of pelletized dry sludge from the rotary kiln thermal drying process at the Barueri (BA) sewage treatment plant.

The sludge collected at SABESP's sewage treatment plants, after undergoing physical and chemical treatment during the solid phase of sewage treatment, is disposed of in the plant's sludge yard in piles and heaps for later transportation and final disposal in landfills. Sampling was carried out in accordance with standard NBR 10007 [1]. In short, three sections of the pile were chosen for collection: top, middle and bottom, from which samples were taken and deposited on a plastic tarpaulin for subsequent quartering. The sludge samples from the three stations were packed in 200 L metal drums in order to reduce health risks, given the high concentration of pathogens and toxic metals present.

Preparation of the SSA

The transformation of sludge into SSA involves a natural drying process on drying beds, together with drying in a rotary kiln coupled to a gas scrubber before calcination. This process is necessary not only to eliminate the water, but also to remove some of the organic matter. The dewatered sludge has more than 70% humidity and, without prior drying, there is a very high risk of the muffle furnace, used to carry out the thermal treatment of sewage sludge, being compromised. This is due to the impregnation of organic matter in the refractory ceramic inside the equipment. Because of the amount of sludge collected, which would need to go through the drying process, a gas-heated dryer was designed and built (drying capacity of 1 kg h^{-1}). The emission of toxic gases was reduced by attaching a gas scrubber. This system is shown in Fig. S1.

After the drying process, the sludge was calcined for 6 h at 650 °C in a muffle furnace (Prolab, Brazil). The time and temperature were determined by means of tests in which samples from the three sewage treatment plants were calcined for periods of time between 1-8 h, with an increment of 1 h and temperatures between 350-950°C, with increments of 100ºC. Obtaining a product capable of reacting and forming a geopolymer was the criterion adopted for defining the drying and calcination temperatures and times. The ideal values for calcination temperature and time were 650ºC and 6 h, respectively. After solidification, the geopolymers were subjected to axial compressive strength tests and water immersion tests for a period of 24 h.

To complete the process of preparing the SSA, the samples were ground in a ball mill manufactured by the Center for Materials Science and Technology (CECTM/IPEN) for 4 h and then sieved in a Quimis electromechanical sieve for 2 h. Samples with grain diameters of 0.045, 0.077, and 0.106 mm were selected. Based on this sequence of preparation steps, the sewage sludge obtained from three different treatment plants resulted in the so-called SSA, as shown in Figures S2-S4.

Feasibility of geopolymers with different precursors

The second stage of the work aimed to investigate the feasibility of using geopolymers, exploring the combination of MK as the main precursor and the addition of SSA to improve its properties. It was considered that the axial compressive strength of the geopolymer with SSA alone was within the limit recommended by the CNEN standard NN 6.09 [2], 10 MPa.

The synthesis of the standard MK-based geopolymer was carried out following the conventional steps for MK activation. The required alkalinity was obtained by precisely mixing the activating solutions. The curing time and thermal curing conditions were optimized to guarantee the efficient formation of the geopolymer, ensuring a solid and stable matrix.

The addition of SSA to the standard geopolymer was an important step in modifying and improving the final properties of the geopolymer. Various concentrations of SSA were tested in an attempt to identify the ideal proportion that would provide the resulting material with greater mechanical strength, durability, and thermal stability. This phase was performed systematically, carefully recording the variations in the geopolymer's characteristics as the amount of SSA varied.

Factorial design

The effect of different experimental conditions on the manufacture of geopolymer was evaluated using the axial compressive strength as the dependent response (*Y*, MPa). To do this, a fractional factorial design was used with the operating conditions described in Table S3.

Five parameters that may affect the axial compressive strength of the geopolymer were studied: X_1 = MK mass, X_2 = SA, X_3 = mass of sand, X_4 = mass of water, X_5 = mass of lime. The minus (-) and plus (+) symbols were used to designate low and high levels, respectively. The experiments were conducted using a 2^{5-1} factorial design, totaling sixteen different experimental conditions. The experiments for the two-level factorial design were carried out according to the methodology. All the variables are numerical, allowing for adjustments at any level. The results of the experimental design were processed using R [3] in the IDLE RStudio software to evaluate the effects, as well as the statistical parameters and statistical graphs

(Pareto chart and contour curves). The "pid" packages were used [4] and the "lm" function, belonging to R's "stats". Both coded and real values for each variable are shown in Table S3. Interactions between the independent variables were determined using analysis of variance (ANOVA) and the main effects on axial compressive strength were identified based on the p-value with > 95% confidence level. The complete coded equation Eq. (S1) was used to explain the 2^{5-1} factorial design in the manufacture of geopolymers.

$$Y = \beta_0 + \beta_1 A_1 + \beta_2 A_2 + \beta_3 A_3 + \beta_4 A_4 + \beta_5 A_5 + \beta_6 A_1{:}A_2 + \beta_7 A_1{:}A_3 + \beta_8 A_1{:}A_4 + \beta_9 A_1{:}A_5 + \beta_{10} A_2{:}A_3 + \beta_{11} A_2{:}A_4 + \beta_{12} A_2{:}A_5 + \beta_{13} A_3{:}A_4 + \beta_{14} A_3{:}A_5 + \beta_{15} A_4{:}A_5 \quad (1)$$

where Y is the predicted response, β_0 represents the overall mean, β_i is the regression coefficient related to the interactions and the main variables A_1 = mass of MK, A_2 = mass of AS, A_3 = mass of sand, A_4 = mass of water, A_5 = mass of lime. The variables A_1, A_2, A_3, A_4, A_5 each represent a main effect, referring to the primary variables of interest. The variables $A_1{:}A_2$, $A_1{:}A_3$, $A_1{:}A_4$, and so on, represent the effects of interactions. Interactions with three or more variables were not considered.

Text S2

Calculation of cesium activity

The activity of the original ^{137}Cs solution was 2.54 × 10^5 Bq, and its total mass was 50.45 g. The original concentration of the ^{137}Cs solution was calculated from Eq. (S1):

$$C = \frac{A}{m_T} \quad \text{(S1)}$$

$$= \frac{2.54 \times 10^5 Bq}{50.45\ g} = 5.03 \times 10^3 \frac{Bq}{g}$$

where A is the activity of the cesium solution and m_T its total mass. An initial mass (m_0) of 1.11 g was collected from this solution, and its initial activity (A_0) was calculated from Eq. (S2):

$$A_0 = C\ m_0 \quad \text{(S2)}$$

$$= 5.03 \times 10^3 \frac{Bq}{g} 1.11\ g = 5.6 \times 10^3 Bq\ theoretical$$

$$A_o = 5{,}7 \times 10^3 Bq\ experimental$$

This same sample was then diluted in a mass of water (m_{H_2O}) of 31.81 g, where C_0 (Bq g^{-1}) is the concentration of the diluted sample, according to Eq. (S3):

$$C_0 = \frac{A_0}{m_{H_2O}} \quad \text{(S3)}$$

$$= \frac{5.6 \times 10^3 Bq}{31.81\ g} = 1.80 \times 10^2 \frac{Bq}{g}$$

The theoretical activity was then measured using a sample mass of 1.87g (m_{sample}). This was the same value as the mass measured experimentally later to obtain its real value. The theoretical activity ($A_{0,sample}$) was calculated from the concentration of the diluted sample and the mass weighed for its actual measurement, as shown in Eq.(S4):

$$A_{o(sample)} = C_0\ m_{sample} \quad \text{(S4)}$$

$$= 1.80 \times 10^2 \frac{Bq}{g}\ 1.87\ g = 3.37 \times 10^2 Bq = \ 3.4 \times 10^2 Bq$$

Text S3

Leaching tests

The cesium leaching characteristics of the geopolymer samples were evaluated based on the leaching rate (LR) and the cumulative leached fraction (CLF). The LRn is defined as the amount of a constituent of a solid material that is leached during a given time interval. The LR was measured from the ^{137}Cs leached from the geopolymer samples and was calculated from Eq. (S5) [5,6]):

$$LR = \frac{\sum_n a_n}{A_0}\left(\frac{V}{S}\right)\left(\frac{1}{t}\right) \tag{S5}$$

Where a_n is the amount, in Bq, of a nuclide released from the sample during the leaching interval n, A_0 is the total activity of a given nuclide in the sample, V is the volume of the sample (cm^3), S is the surface area of the sample (cm^2), and t is the leaching time since the start of the leaching test (day).

Figure S5 shows the leaching behavior of the geopolymer samples expressed as LR as a function of time. LR can be described as the fraction dependent on the volume-surface ratio of the sample of the initial activity of the leached constituent, with its average calculated over the leaching time. For cylindrical specimens with a diameter of 5 cm and a height of 10 cm, LR is numerically equal to the total fraction of the constituent leached during the leaching test, divided by the leaching time, expressed in cm day^{-1}. The fraction cumulative leached (FCL_N) is defined as the sum of the fractions of the initial activity of the nuclide contained in the sample, leached during all previous leaching intervals, including the N interval. The FCL_N of ^{137}Cs was calculated from Eq. (S6) [5]:

$$FCL_N = \frac{\sum_1^N a_n}{A_0} \tag{S6}$$

Text S4

Analysis of the crystalline phases

For the qualitative and quantitative analysis of the crystalline phases present, crystallinity content and analysis of the crystalline structure of the material, the results were compared with the reference diffraction in standard database files using Bruker's Diffrac Suite Topas version 5.0 software (quantitative analysis) and the Rietveld method. The Diffrac Suite Topas software was used to analyze the X-ray diffraction results. The libraries used to determine the crystalline phases were: quartz (SiO_2, PDFcard#461045), kaolinite($Al_2Si_2O_5(OH)_4$, PDFcard#140164), muscovite ($KAl_3Si_3O_{10}(OH)_2$, PDFcard#210993) and hematite (Fe_2O_3, PDFcard#130534) were found in the standard MK; quartz (SiO_2, PDFcard#461045), kaolinite ($Al_2Si_2O_5(OH)_4$, PDFcard#140164), muscovite ($KAl_3Si_3O_{10}(OH)_2$, PDFcard#210993), hematite (Fe_2O_3,PDFcard#130534) and faujasite ($Na_2Al_2Si_4O_{12}$ $8H_2O$, PDFcard#391380).

Text S5

Equations from the factorial experimental design

By applying a linear model based on a factorial experimental design, two equations were generated, one for each curing condition:

Curing at room temperature	$Y_1 = 11.23 + 0.15X_{1codif} + 1.06X_{2codif} + 0.52X_{3codif} - 0.02X_{4codif} - 0.15X_{5codif} + 0.23X_{1codif}{:}X_{2codif} + 0.27X_{2codif}{:}X_{3codif} - 0.19X_{2codif}{:}X_{4codif} + 0.35\ X_{2codif}{:}X_{5codif} - 0.15X_{1codif}{:}X_{3codif} - 0.56X_{3codif}{:}X_{4codif} + 0.06X_{3codif}{:}X_{5codif} - 0.52X_{1codif}{:}X_{4codif} + 0.35X_{4codif}{:}X_{5codif}$	(S1)
Initial curing at 60 ºC/6h	$Y_2 = 15.98 + 0.02X_{1codif} + 0.9X_{2codif} + 0.56X_{3codif} + 0.15X_{4codif} - 0.31X_{5codif} + 0.44X_{1codif}{:}X_{2codif} + 0.31X_{2codif}{:}X_{3codif} + 0.4X_{2codif}{:}X_{4codif} -0.23X_{2codif}{:}X_{5codif} + 0.52X_{1codif}{:}X_{3codif} - 0.77X_{3codif}{:}X_{4codif} - 0.56X_{1codif}{:}X_{4codif} + 0.77X_{4codif}{:}X_{5codif}$	(S2)

Text S6

Sieving and granulometry

Particles with a diameter between 0.077 and 0.106 mm gave the best results during the mixing process and during geopolymerization. The size of the particles influences the process, because large particles can make it difficult for the alkaline solution to pass into deeper regions. With large particles, the contact area is smaller, making it more difficult for the activator solution to interact with the SSA and MK. The particles that receive AS and undergo dissolution precipitate and organize themselves in the geopolymer structure by means of a chemical reaction. After hardening, the reaction does not continue, which impairs mobility and diffusion capacity. As a result, the smaller particles become trapped in the matrix and are unable to receive the alkaline solution homogeneously, participating in the reaction only as an aggregate. The regions that do not react weaken the final structure of the product. On the other hand, the smaller particles mostly increase the contact area and consequently geopolymerization [7,8].

Table S1 Experimental conditions for the manufacture of geopolymers. Initial pH > 12

		Sludge drying		Calcination		Cure	
	**WWTP**	**Temperature**	**Time**	**Temperature**	**Time**	**Temperature**	**Time**
		(ºC)	(h)	(ºC)	(h)	(ºC)	(h)
E1	BA	-	6	350	2	60	2
E2	BA	-	6	450	2	60	2
E3	BA	80	6	550	2	60	2
E4	BA	80	6	550	2	60	2
E5	BA	-	6	600	2	60	2
E6	BA	40	2	650	2	40	2
E7	BA	60	4	650	2	50	3
E8	BA	100	6	650	3	50	4
E9	BA	200	6	650	4	60	5
E10	BA	380	24	650	6	60	6
E11	BA	-	6	750	2	60	6
E12	BA	-	6	850	2	60	6
E13	BA	-	6	950	2	60	6
E14	BA	-	6	950	5	60	6
E15	PNM	-	-	350	2	60	2
E16	PNM	-	-	450	2	60	2
E17	PNM	80	24	550	2	60	2
E18	PNM	80	24	550	3	60	2
E19	PNM	-	-	600	2	60	2
E20	PNM	-	-	630	6	40	2
E21	PNM	100	24	650	2	50	3
E22	PNM	100	24	650	2	50	4
E23	PNM	200	24	650	3	60	5
E24	PNM	380	24	650	6	60	6
E25	PNM	-	-	750	2	60	6
E26	PNM	-	-	850	2	60	6
E27	PNM	-	-	950	2	60	6
E28	PNM	-	-	950	5	60	6
E29	BP	-	-	630	6	60	2
E30	BP	-	-	350	2	60	2
E31	BP	-	-	450	2	60	2
E32	BP	80	24	550	2	60	2
E33	BP	80	24	550	2	60	2
E34	BP	-	-	600	2	40	2
E35	BP	100	24	650	2	50	3
E36	BP	100	24	650	2	50	4
E37	BP	100	24	650	3	60	5
E38	BP	200	24	650	4	60	5
E39	BP	380	24	650	6	60	6
E40	BP	-	-	750	2	60	6
E41	BP	-	-	850	2	60	6
E42	BP	-	-	950	2	60	6
E43	BP	-	-	950	5	60	6

Table S2 Exploratory tests in the manufacture of geopolymer. Initial pH > 12 for all experimental conditions used. NR = did not resist, R = resisted, Y = yes, N = no.

	**Resistence**		**Heat release**		
	**Mechanical**	**Immersion**	**NaOH**		
		**Water (24h)**	**8M**	**12M**	**14M**
E1	NR	NR	Y	N	N
E2	NR	NR	Y	N	N
E3	NR	NR	Y	N	N
E4	NR	NR	Y	N	N
E5	NR	NR	Y	N	N
E6	NR	NR	Y	N	N
E7	NR	NR	Y	N	N
E8	R	NR	Y	N	N
E9	R	NR	Y	N	N
E10	R	R	Y	Y	Y
E11	NR	NR	Y	N	N
E12	NR	NR	Y	N	N
E13	NR	NR	Y	N	N
E14	NR	NR	Y	N	N
E15	NR	NR	N	N	N
E16	NR	NR	N	N	N
E17	NR	NR	N	N	N
E18	NR	NR	N	N	N
E19	NR	NR	N	N	N
E20	NR	NR	N	N	N
E21	NR	NR	N	N	N

Table S3 Real and coded values in the project matrix for X_1 = MK mass, X_2 = AS, X_3 = sand mass, X_4 = water mass, X_5 = lime mass; X_5 encodings were calculated from X_1*X_2*X_3*X_4. The unit of masses is grams.

Standard Order	X_1 (cod.)	X_2 (cod.)	X_3 (cod.)	X_4 (cod.)	X_5 (cod.)	X_1 (real)	X_2 (real)	X_3 (real)	X_4 (real)	X_5 (real)
1	-	-	-	-	+	100	90	80	10	20
2	+	-	-	-	-	120	90	80	10	3
3	-	+	-	-	-	100	105	80	10	3
4	+	+	-	-	+	120	105	80	10	20
5	-	-	+	-	-	100	90	120	20	3
6	+	-	+	-	+	120	90	120	20	20
7	-	+	+	-	+	100	105	120	20	20
8	+	+	+	-	-	120	105	120	20	3
9	-	-	-	+	-	100	90	80	10	3
10	+	-	-	+	+	120	90	80	10	20
11	-	+	-	+	+	100	105	80	10	20
12	+	+	-	+	-	120	105	80	10	3
13	-	-	+	+	+	100	90	120	20	20
14	+	-	+	+	-	120	90	120	20	3
15	-	+	+	+	-	100	105	120	20	3
16	+	+	+	+	+	120	105	120	20	20

Table S4 Geopolymer formulation. Activating solution = 1.16 g (8 M), NaOH mass = 0.10 g, $CsNO_3$ mass = 0.10 g, water mass = 0.51 g, sand mass = 3.33 g, lime mass = 0.13 g, total mass = 6.44 g.

**Samples**	**MK (g)**	**SSA (g)**
Standard	1.11	-
SSA - BA 10%	1.00	0.11
SSA - BA 30%	0.78	0.33
SSA - BP 10%	1.00	0.11
SSA - BP 30%	0.78	0.33

Table S5 Results of the elemental analysis obtained by ICP-OES for the sludge

	Concentration (mg L^{-1})		
**Element**	**BA**	**PNM**	**BP**
Al	21.7	38.0	116.5
B	3.2	0.9	0.3
Ba	0.7	0.7	0.8
Bi	0.1	-	-
Ca	14.2	198.8	22.6
Cu	2.1	3.1	1.3
Fe	16.5	77.0	81.6
Ga	0.3	0.3	0.4
K	0.2	9.5	9.9
Li	2.0	2.0	2.0
Mg	-	13.8	4.1
Na	15.1	34.8	84.0

Table S6 Results of the X-ray fluorescence analysis of the ash obtained from the sludge from the treatment plants. *ND = not determined

	**Content (%)**		
**Compound**	**BA**	**PNM**	**BP**
Al_2O_3	24±1	5.8±0.6	25±1
Na_2O	0.44±0.04	17±1	25±1
SiO_2	35±1	7.6±0.8	21±1
Fe_2O_3	10±1	14±1	13±1
P_2O_5	8.9±0.9	4.0±0.4	10±1
CaO	8.2±0.8	42±1	2.1±0.2
TiO_2	1.8±0.2	0.53±0.05	1.2±0.1
K_2O	2.1±0.4	0.46±0.05	1.2±0.1
MgO	2.0±0.2	1.5±0.2	0.85±0.09
SO_3	5.7±0.6	2.3±0.2	0.46±0.05
ZnO	0.35±0.04	1.3±0.1	0.07±0.01
CuO	0.17±0.02	0.22±0.02	0.03±0.01
Cr_2O_3	0.15±0.02	0.21±0.02	0.03±0.01
NiO	0.08±0.01	0.05±0.01	0.014±0.001
Ag_2O	0.06±0.01	ND	ND
PbO	0.03±0.01	ND	0.013±0.001
Cl	ND*	3.8±0.4	0.04±0.01
As_2O_3	ND	ND	<0.010
Br	ND	ND	<0.010
ZrO_2	0.020±0.002	0.011±0.001	ND
MnO	0.13±0.01	0.06±0.01	ND
BaO	0.19±0.02	ND	ND
SrO	0.017±0.002	ND	ND
Rb_2O	<0.010	ND	ND
Y_2O_3	<0.010	ND	ND

Table S7 R/DRX values - errors and GOF from sewage sludge ash analysis. Prevalence ratio (R_p), R_{wp} = weighted error (%), experimental ratio = R_{exp}, GOF = quality of fit.

**R values**	**BA**	**PNM**	**BP**
R_{exp}	8.38	10.18	8.84
R_{wp}	9.92	19.47	11.65
R_p	7.64	14.92	9.07
GOF	1.18	1.91	1.32

Table S8 Molar ratio between SiO_2 and Al_2O_3. Molecular masses: 101.96 g mol^{-1} (Al_2O_3), 60.08 g mol^{-1} (SiO_2)

**Compound**	**BA**	**BP**
Al_2O_3	20.4	15.8
SiO_2	29.7	13.2
Ratio SiO_2/Al_2O_3	2.5	1.4

* Mass (g) per 100 g of material

Table S9 Compressive strength values for different curing times. Initial cure at 60°C for 6 h.

Exp. Standard Order	Compressive Strength (MPa)	Curing time(d)
BA	12.4±0.6	3
	13.2±0.6	7
	14.5±0.6	15
	14.4±0.6	28
BP	10.8±0.6	3
	12.3±0.6	7
	12.5±0.6	15
	13.7±0.6	28

Table S10 Experiments conducted from two 2^{5-1} fractional factorial experimental designs (total of 16 experiments for each cure condition). X_1 = MK mass, X_2 = AS, X_3 = sand mass, X_4 = water mass, X_5 = lime mass. Curing time = 15 days. CTA = Room Temperature Cure; CT60 = Curing Temperature starting at 60ºC for 6 h. * Mean ± standard deviation

	Encoded					Real					
**Exp. Standard Order**	**X1**	**X2**	**X3**	**X4**	**X5**	**X1**	**X2**	**X3**	**X4**	**X5**	**Compressive strength***
						**(g)**					**(MPa)**
CTA-1	-	-	-	-	+	100	90	120	25	20	8 ± 1
CTA-2	+	-	-	-	-	120	90	120	25	14,5	11 ± 1
CTA-3	-	+	-	-	-	100	125	120	25	14,5	10 ± 1
CTA-4	+	+	-	-	+	120	125	120	25	20	11.67 ± 0.58
CTA-5	-	-	+	-	-	100	90	360	55	14,5	11 ± 1
CTA-6	+	-	+	-	+	120	90	360	55	20	10 ± 1
CTA-7	-	+	+	-	+	100	125	360	55	20	13.33 ± 0.58
CTA-8	+	+	+	-	-	120	125	360	55	14,5	15 ± 1
CTA-9	-	-	-	+	-	100	90	120	25	14,5	11 ± 1
CTA-10	+	-	-	+	+	120	90	120	25	20	9.67 ± 1.53
CTA-11	-	+	-	+	+	100	125	120	25	20	12.67 ± 0.58
CTA-12	+	+	-	+	-	120	125	120	25	14,5	11.67 ± 1.15
CTA-13	-	-	+	+	+	100	90	360	55	20	11 ± 1
CTA-14	+	-	+	+	-	120	90	360	55	14,5	9.67 ± 1.53
CTA-15	-	+	+	+	-	100	125	360	55	14,5	11.67 ± 1.15
CTA-16	+	+	+	+	+	120	125	360	55	20	12.33 ± 0.58
CT60-1	-	-	-	-	+	100	90	120	25	20	14.33 ± 0.58
CT60-2	+	-	-	-	-	120	90	120	25	14,5	15 ± 1
CT60-3	-	+	-	-	-	100	125	120	25	14,5	15 ± 1
CT60-4	+	+	-	-	+	120	125	120	25	20	13.67 ± 0.58
CT60-5	-	-	+	-	-	100	90	360	55	14,5	16.33 ± 0.58
CT60-6	+	-	+	-	+	120	90	360	55	20	15.67 ± 0.58
CT60-7	-	+	+	-	+	100	125	360	55	20	15.33 ± 0.58
CT60-8	+	+	+	-	-	120	125	360	55	14,5	21.33 ± 0.58
CT60-9	-	-	-	+	-	100	90	120	25	14,5	15.67 ± 0.58
CT60-10	+	-	-	+	+	120	90	120	25	20	14.33 ± 0.58
CT60-11	-	+	-	+	+	100	125	120	25	20	18.67 ± 0.58
CT60-12	+	+	-	+	-	120	125	120	25	14,5	16.67 ± 0.58
CT60-13	-	-	+	+	+	100	90	360	55	20	15.67 ± 0.58
CT60-14	+	-	+	+	-	120	90	360	55	14,5	13.67 ± 0.58
CT60-15	-	+	+	+	-	100	125	360	55	14,5	16.67 ± 0.58
CT60-16	+	+	+	+	+	120	125	360	55	20	17.67 ± 0.58

Table S11 Statistical summary of the fits of the multiple linear models applied. [a] Significance codes: 0 '***' 0.001 '**' 0.01 '*' 0.05 '.' 0.1 ' ' 1

	**Estimate**	**Standard error**	**t value**	**Pr(>|t|)**	**Code[a]**
	Curing condition starts at room temperature				
(Intercept)	11.23	0.2023	55.507	1.29E-05	***
X_{1codif}	0.1462	0.2023	0.723	0.522	
X_{2codif}	1.0625	0.2023	5.252	0.0134	*
X_{3codif}	0.52	0.2023	2.57	0.0825	.
X_{4codif}	-0.02	0.2023	-0.099	0.9275	
X_{5codif}	-0.1462	0.2023	-0.723	0.522	
X_{1codif}:X_{2codif}	0.2288	0.2023	1.131	0.3404	
X_{2codif}:X_{3codif}	0.27	0.2023	1.335	0.2743	
X_{2codif}:X_{4codif}	-0.1875	0.2023	-0.927	0.4224	
X_{2codif}:X_{5codif}	0.3538	0.2023	1.748	0.1787	
X_{3codif}:X_{4codif}	-0.5625	0.2023	-2.78	0.069	.
X_{1codif}:X_{4codif}	-0.5212	0.2023	-2.576	0.082	.
X_{4codif}:X_{5codif}	0.3538	0.2023	1.748	0.1787	
R^2	0.95				
	Curing condition starts at 60 ºC/6h				
(Intercept)	15.98	0.27034	59.111	0.000286	***
X_{1codif}	0.02125	0.27034	0.079	0.944503	
X_{2codif}	0.89625	0.27034	3.315	0.08019	.
X_{3codif}	0.5625	0.27034	2.081	0.172946	
X_{4codif}	0.1475	0.27034	0.546	0.640051	
X_{5codif}	-0.3125	0.27034	-1.156	0.367129	
X_{1codif}:X_{2codif}	0.4375	0.27034	1.618	0.246998	
X_{2codif}:X_{3codif}	0.31125	0.27034	1.151	0.36865	
X_{2codif}:X_{4codif}	0.39625	0.27034	1.466	0.280352	
X_{2codif}:X_{5codif}	-0.22875	0.27034	-0.846	0.486558	
X_{1codif}:X_{3codif}	0.52125	0.27034	1.928	0.193642	
X_{3codif}:X_{4codif}	-0.77	0.27034	-2.848	0.104327	
X_{1codif}:X_{4codif}	-0.56375	0.27034	-2.085	0.172367	
X_{4codif}:X_{5codif}	0.77	0.27034	2.848	0.104327	
R^2	0.96				

Table S12 Evolution of axial compressive strength (MPa) from 3 to 28 days. Standard and ash samples of sewage sludge from BA and BP WWTPs were used. The experimental conditions are those from experimental runs No. 8 shown in Table S10.

	**Compressive strenght* (MPa)**	**Curing (days)**
	**MK only**	
	**Curing at 60ºC/6h**	
Standard	17.67 ± 0.58	3
	19.33 ± 0.58	7
	21.33 ± 0.58	15
	22.39 ± 0.58	28
	**Curing in water**	
	11.67 ± 0.58	3
	10.67 ± 0.58	7
	10.33 ± 0.58	15
	10.47 ± 0.58	28
	**Only sewage sludge at 60ºC/6h**	
BA	12.33 ± 0.58	3
	13.33 ± 0.58	7
	13.67 ± 0.58	15
	14.33 ± 0.58	28
BP	10.00 ± 0.00	3
	11.67 ± 0.58	7
	11.67 ± 0.58	15
	12.67 ± 0.58	28

Table S13 Degree of crystallinity of the prepared geopolymers

		Amorphous (%)	Crystalline (%)
MK - standard		31.41	68.59
BA	SSE-10	43.77	56.23
	SSE-30	37.67	62.33
	SSE-100	49.06	50.94
BP	SSE-10	41.67	58.33
	SSE-30	46.62	53.38
	SSE-100	38.64	61.36

Table S14 EDS for the geopolymer with different additions of SSA with simulated waste with AC or IER. For the EDS spectra, refer to Fig. S19-S21.

Simulated material	SSA addition (%)	Element	Mass (g)	Element (%)
AC	10	O	49.1 ± 0.3	56.9 ± 0.7
		Na	13.6 ± 0.3	12.0 ± 0.3
		Al	7.5 ± 0.3	8.9 ± 0.2
		Si	28.6 ± 0.4	21.2 ± 0.3
		Ca	1.2 ± 0.1	1.0 ± 0.1
		Total	100.0	100.0
	30	O	41.0 ± 0.3	42.0 ± 0.7
		Na	6.5 ± 0.3	7.0 ± 0.3
		Al	7.3 ± 0.3	6.9 ± 0.2
		Si	19.6 ± 0.4	21.2 ± 0.3
		P	0.8 ± 0.2	1.0 ± 0.2
		K	1.6 ± 0.2	1.3 ± 0.1
		Ca	1.6 ± 0.1	1.0 ± 0.1
		Fe	1.6 ± 0.1	1.0 ± 0.2
		Ag	1.0 ± 0.1	1.3 ± 0.1
		La	2.1 ± 0.2	2.0 ± 0.1
		Ce	7.9 ± 0.2	6.2 ± 0.2
		Nd	9.0 ± 0.1	9.1 ± 0.1
		Total	100.0	100.0
IER	10	O	26.3 ± 0.2	27.3 ± 0.1
		Na	6.9 ± 0.1	6.1 ± 0.2
		Al	8.7± 0.1	7.5 ± 0.2
		Si	58.1 ± 0.3	59.1 ± 0.1
		Total	100.0	100.0
	30	O	13.0 ± 0.1	12.2 ± 0.2
		Na	10.8 ± 0.1	9.9 ± 0.2
		Al	15.9 ± 0.1	16.8 ± 0.1
		Si	60.3 ± 0.2	61.1 ± 0.2
		Total	100.0	100.0

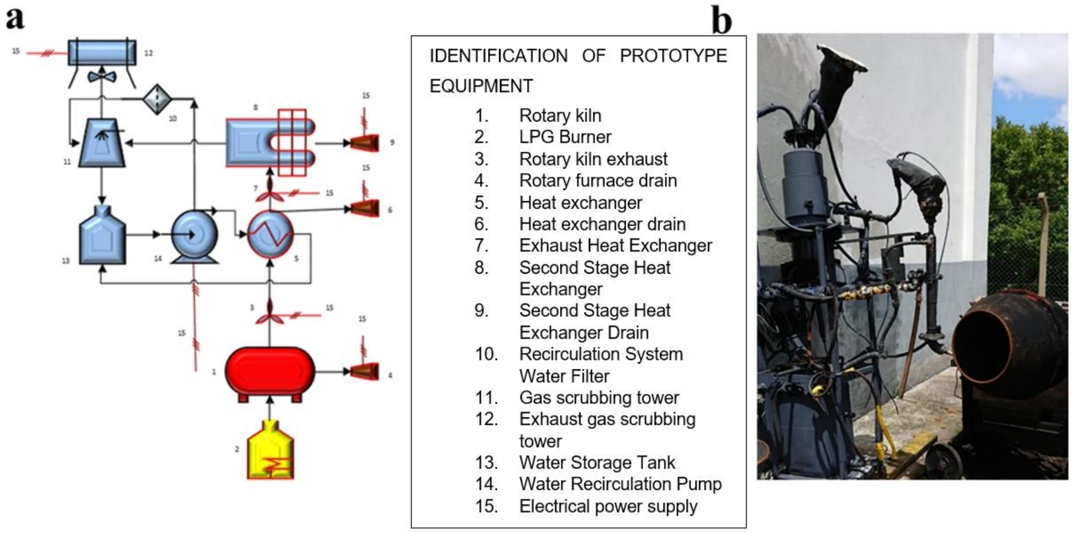

Fig. S1 - Prototype (a) sludge dryer with the gas scrubbing system (b) flow scheme of the drying and scrubbing process of the gases.

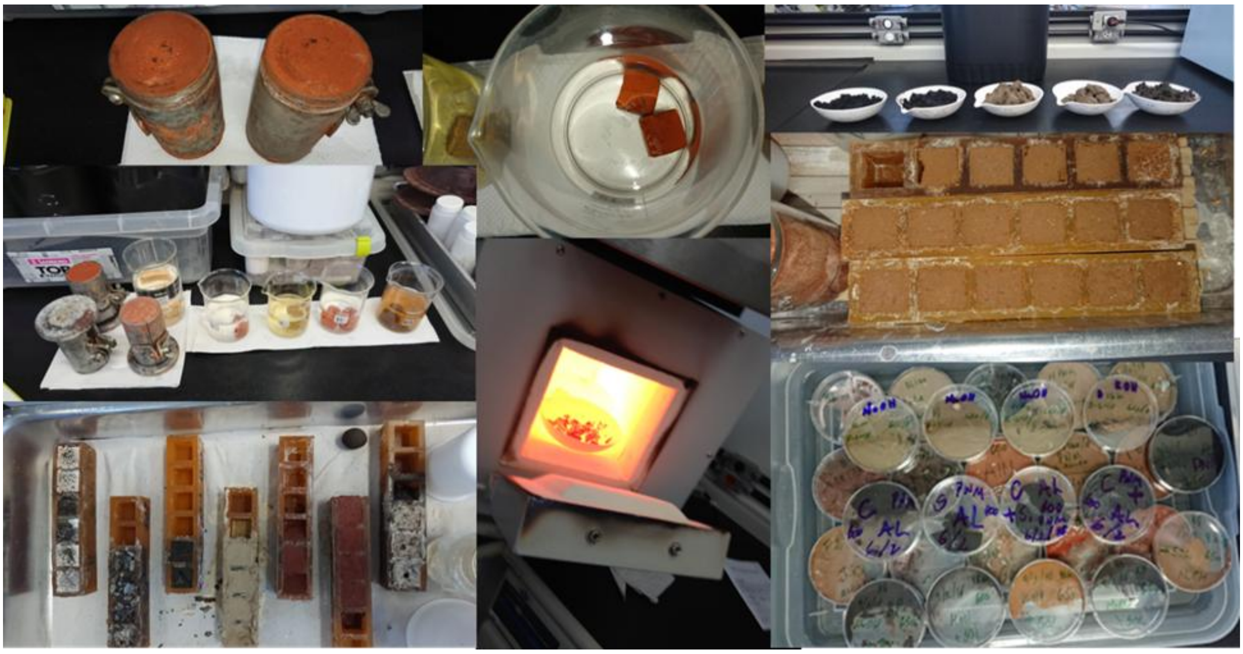

Fig. S2 Exploratory tests: calcination temperature, calcination time and activator solution concentration.

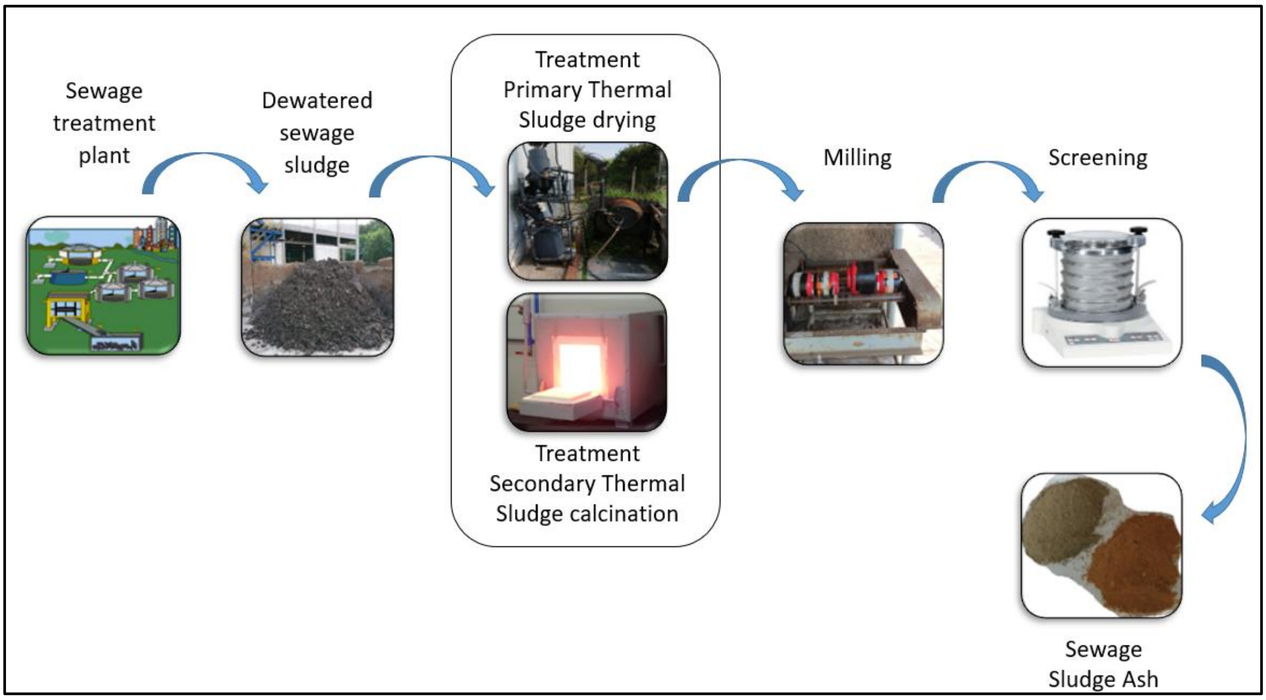

Fig. S3 Flow chart for obtaining sewage sludge ash

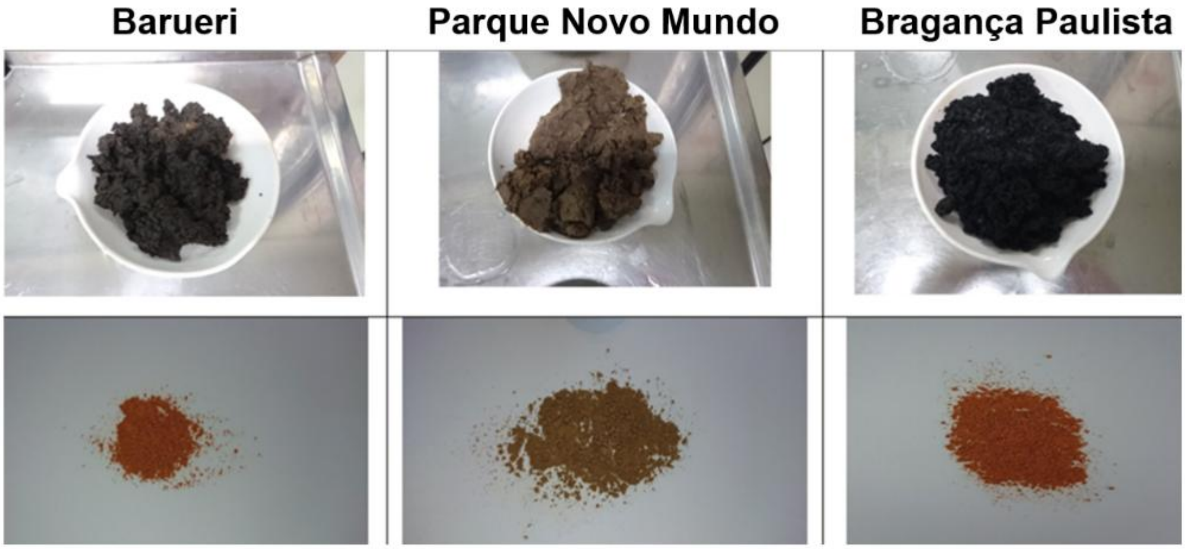

Fig. S4 Ashes from sewage samples obtained from different sewage treatment plants.

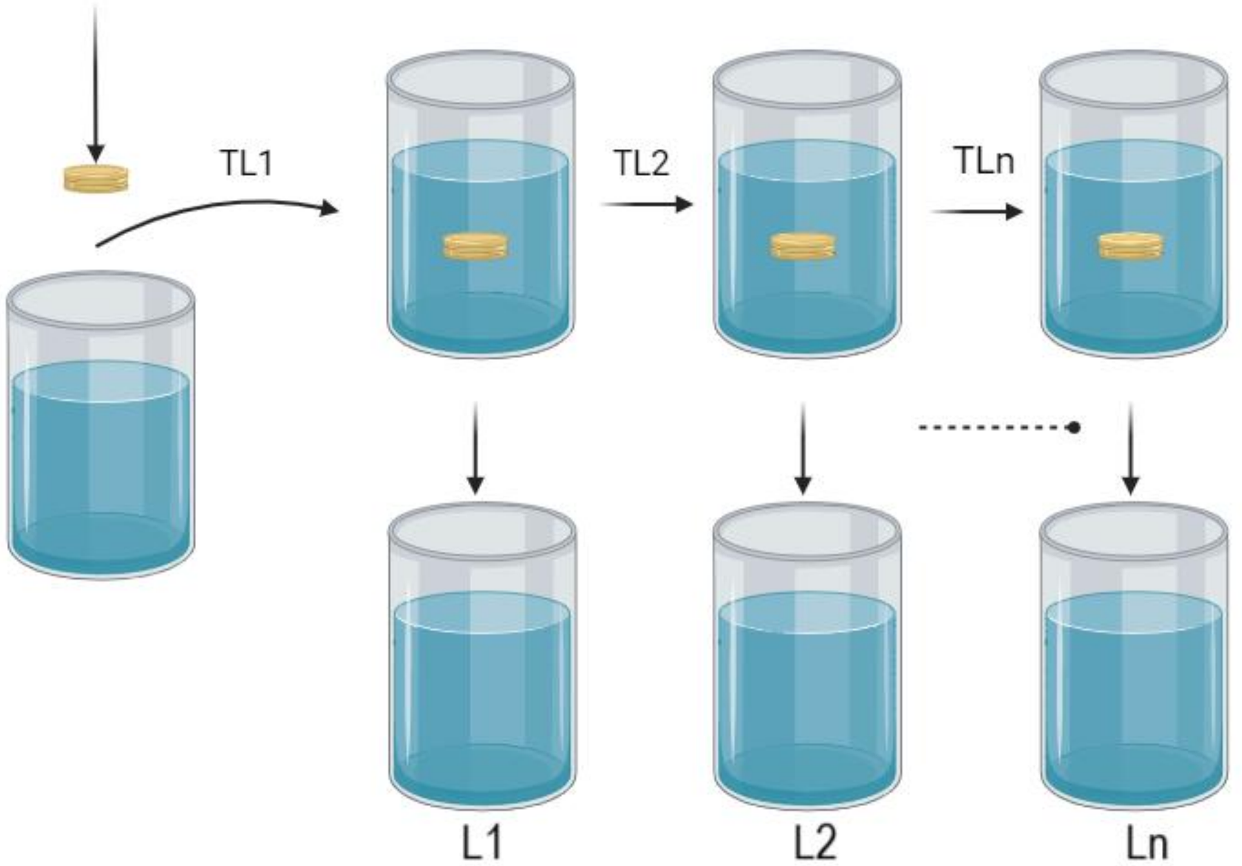

Figure S5 Scheme of leaching tests

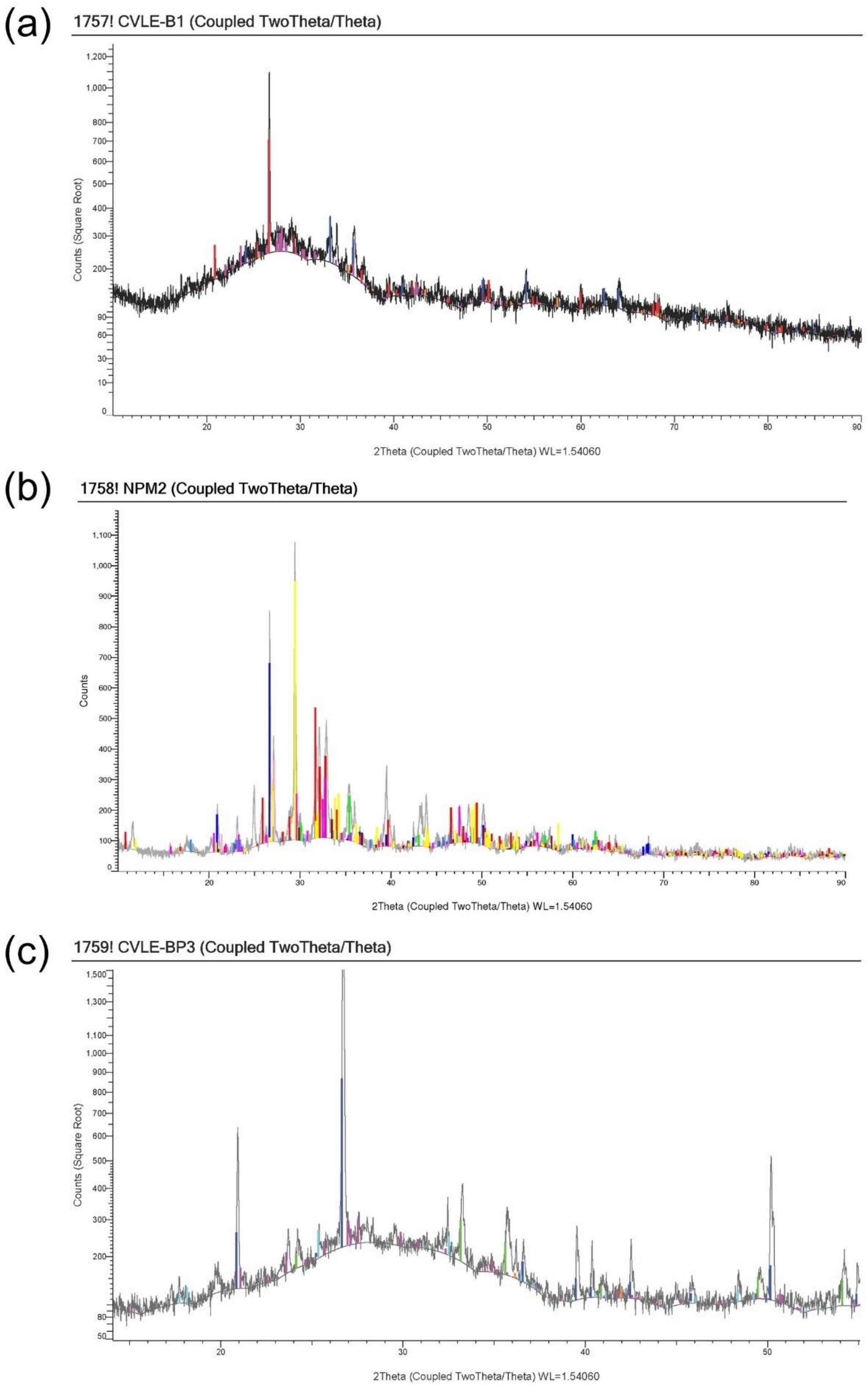

Figure S6 Diffractogram of the SSA obtained from three WWTPs: (a) BA; (b) PNM; (c) BP

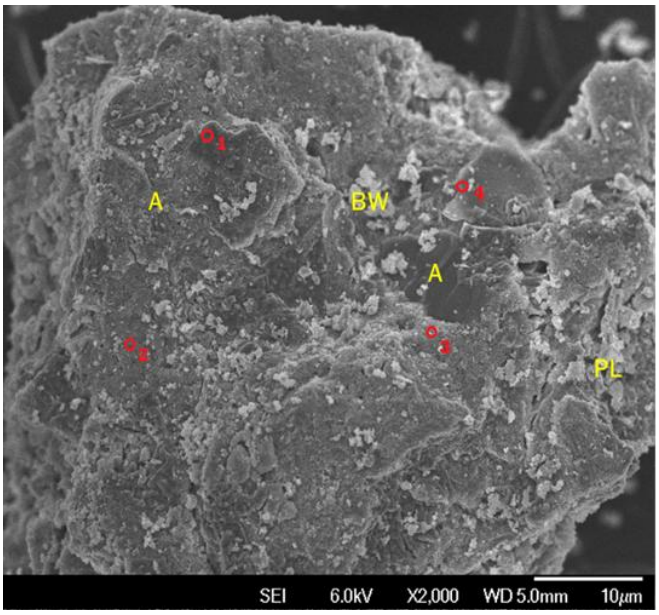

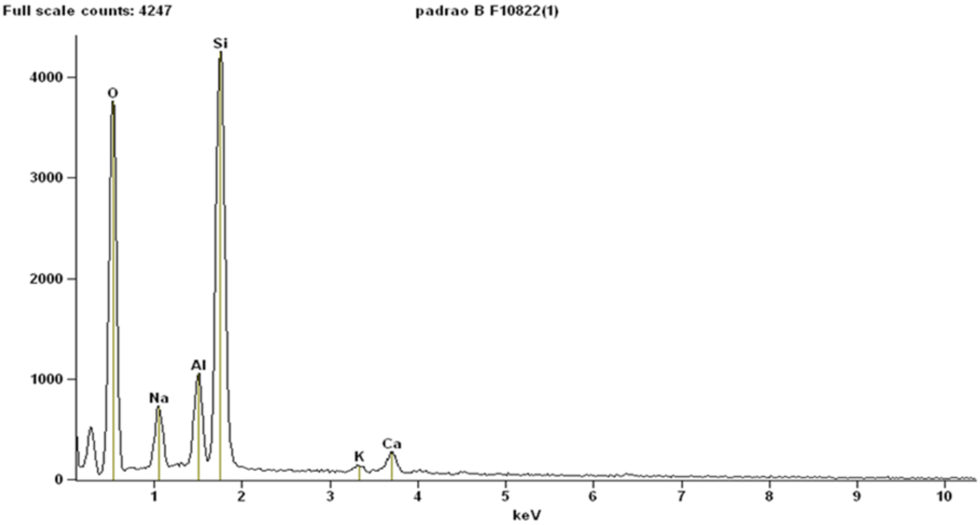

Element	Mass (g)	Element (%)
O	55.4 ± 0.4	68.2 ± 0.5
Na	7.0 ± 0.2	6.0 ± 0.1
Al	5.5 ± 0.1	4.0 ± 0.1
Si	28.4 ± 0.2	20.0 ± 0.2
K	0.8 ± 0.1	0.4 ± 0.1
Ca	2.8 ± 0.2	1.4 ± 0.1
Total	100.0	100.0

Fig. S7 SEM for standard geopolymer with MK and no added sewage sludge ash; EDS for standard geopolymer with MK and no added sewage sludge ash

*A, WB, PL: Crystallization points.

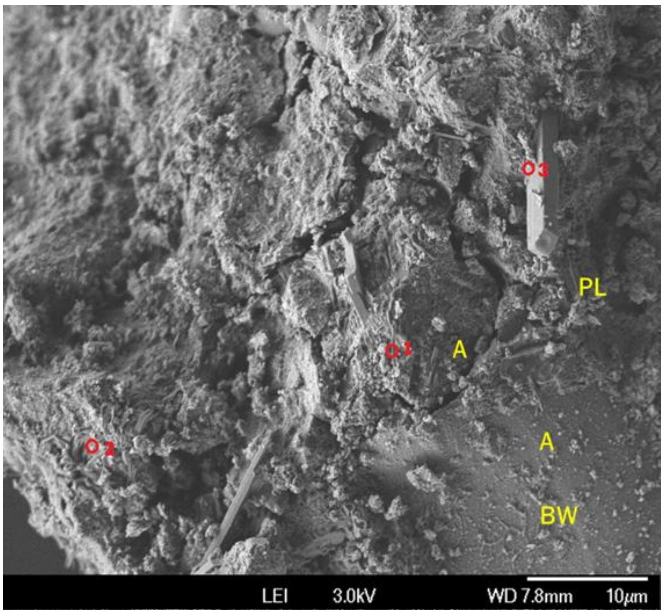

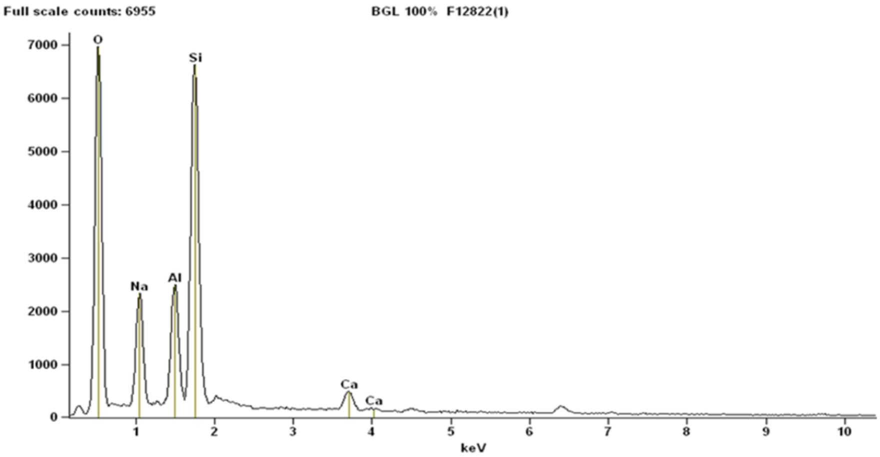

Element	Mass (g)	Element (%)
O	52.5 ± 0.3	64.9 ± 0.3
Na	12.0 ± 0.1	10.3 ± 0.1
Al	8.0 ± 0.1	5.9 ± 0.1
Si	24.9 ± 0.2	17.6 ± 0.1
Ca	2.6 ± 0.1	1.3 ± 0.1
Total	100.0	100.0

Fig. S8 Geopolymer SEM with addition of 100% BA sewage sludge ash; Geopolymer EDS with addition of 100% BA sewage sludge ash

*A, WB, PL: Crystallization points.

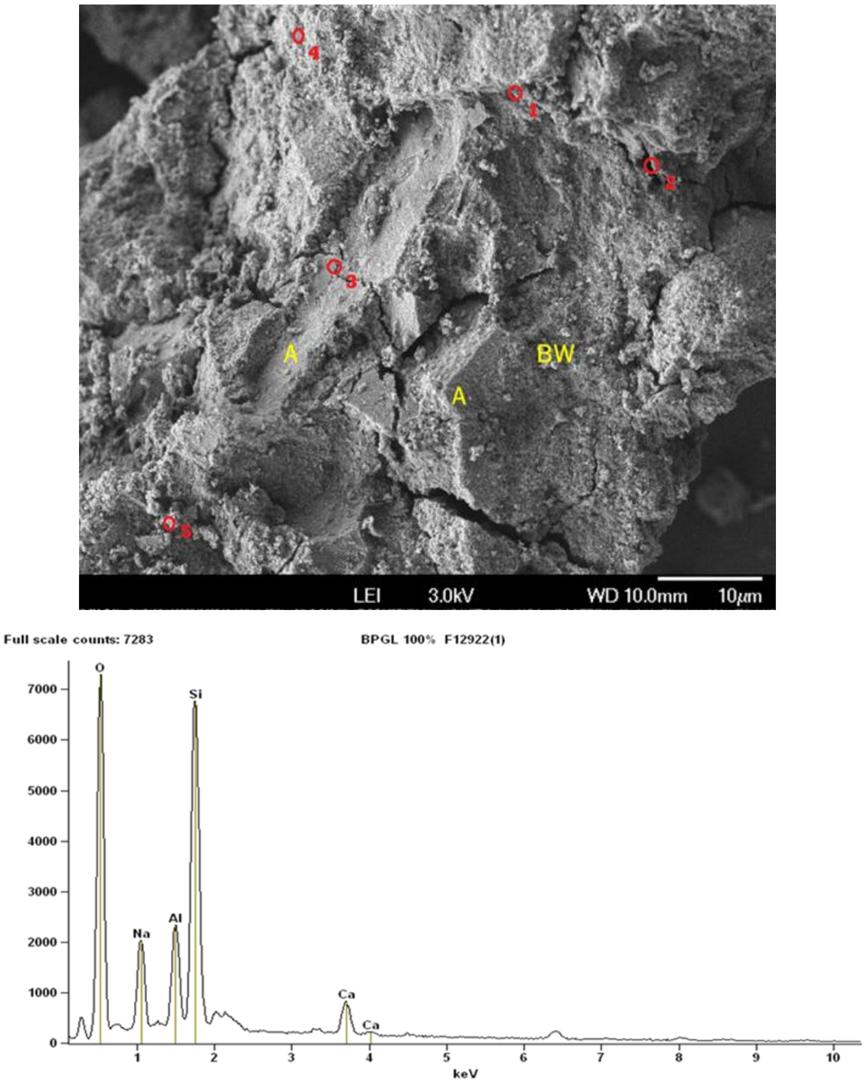

Element	Mass (g)	Element (%)
O	54.3 ± 0.3	67.1 ± 0.3
Na	10.6 ± 0.1	9.1 ± 0.1
Al	6.6 ± 0.1	4.8 ± 0.1
Si	23.8 ± 0.2	16.7 ± 0.1
Ca	4.7 ± 0.1	2.3 ± 0.1
Total	100.0	100.0

Fig. S9 SEM of the geopolymer with the addition of 100% BP sewage sludge ash; Geopolymer EDS with addition of 100% BP sewage sludge ash

*A, WB, PL: Crystallization points.

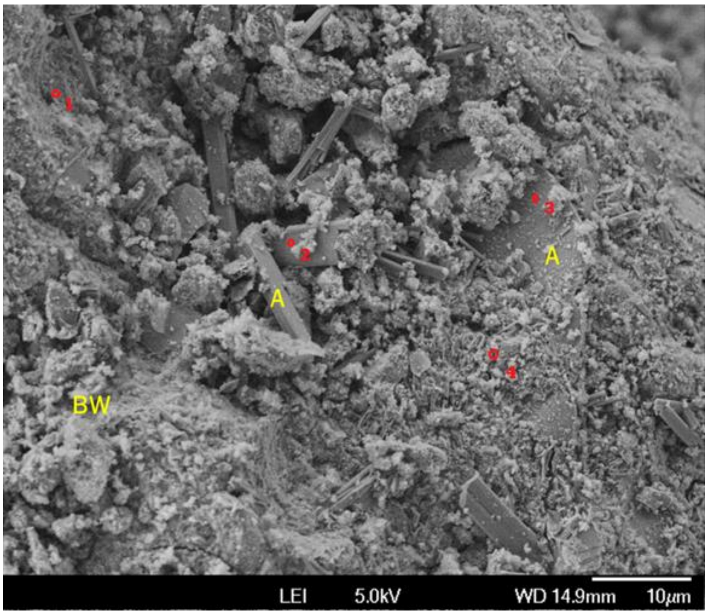

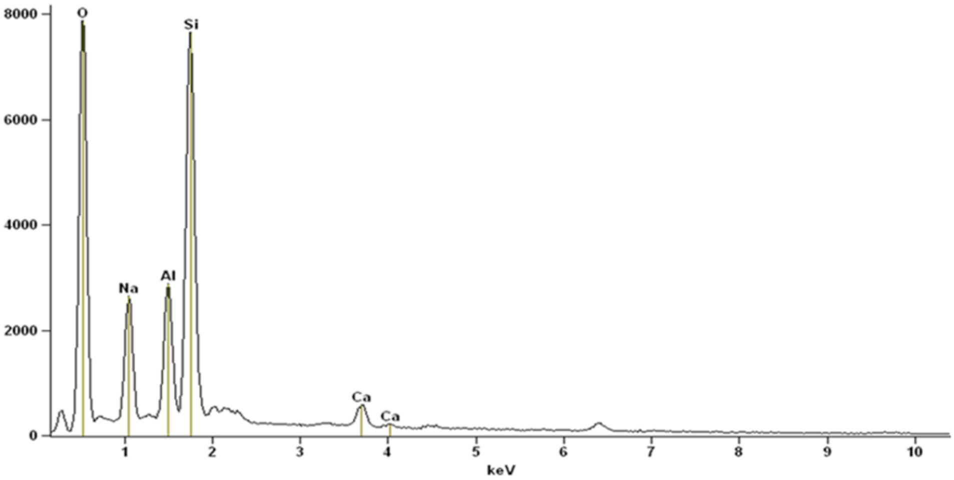

Element	Mass (g)	Element (%)
O	52.6 ± 0.3	65.1 ± 0.3
Na	12.3 ± 0.1	10.6 ± 0.1
Al	7.7 ± 0.1	5.7 ± 0.1
Si	24.5 ± 0.2	17.3 ± 0.1
Ca	2.8 ± 0.1	1.4 ± 0.1
Total	100.0	100.0

Fig. S10 SEM of the geopolymer with the addition of 10% of BA sewage sludge ash; Geopolymer EDS with the addition of 10% BA sewage sludge ash

*A, WB, PL: Crystallization points.

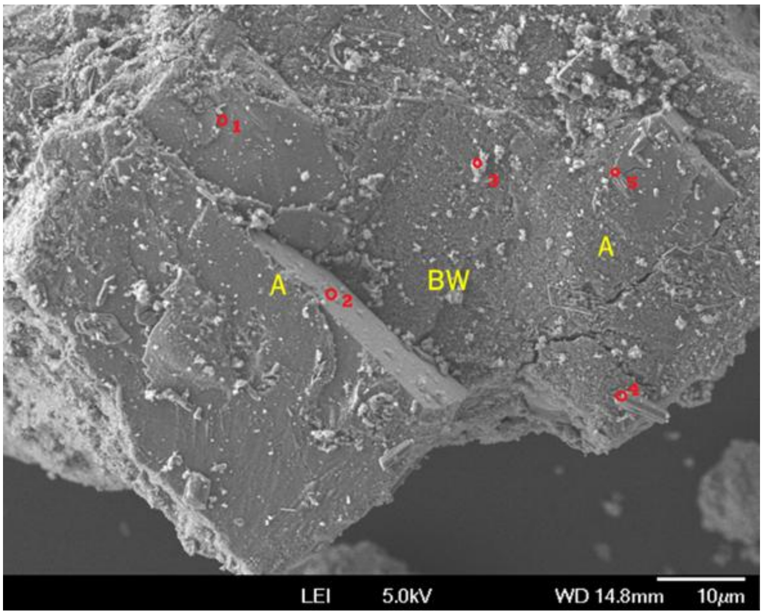

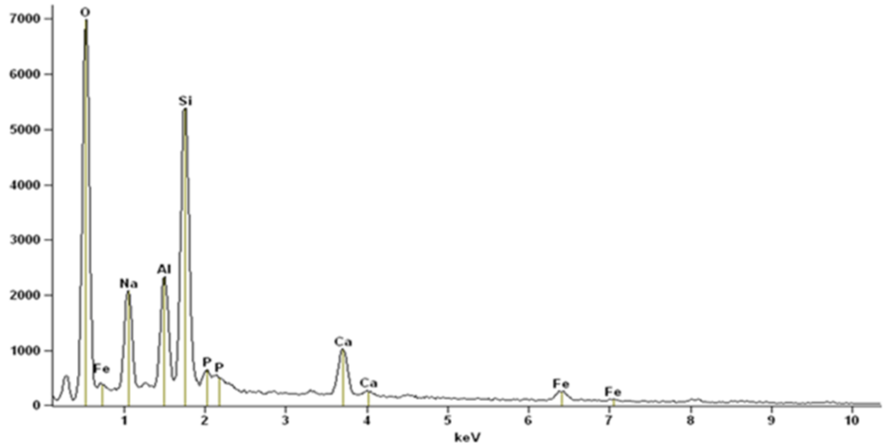

Element	Mass (g)	Element (%)
O	52.5 ± 0.3	66.5 ± 0.4
Na	11.0 ± 0.1	9.7 ± 0.1
Al	6.8 ± 0.1	5.1 ± 0.1
Si	18.7 ± 0.2	13.5 ± 0.1
P	0.8 ± 0.1	0.5 ± 0.0
Ca	6.1 ± 0.1	3.1 ± 0.1
Fe	4.1 ± 0.3	1.5 ± 0.1
Total	100.0	100.0

Fig. S11 SEM of the geopolymer with the addition of 10% ash from sewage sludge BP; Geopolymer EDS with the addition of 10% ash from BP sewage sludge

*A, WB, PL: Crystallization points.

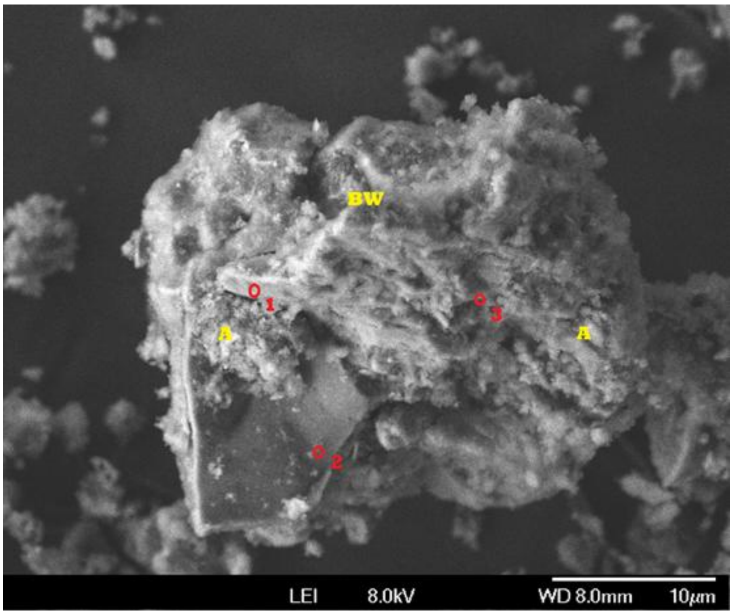

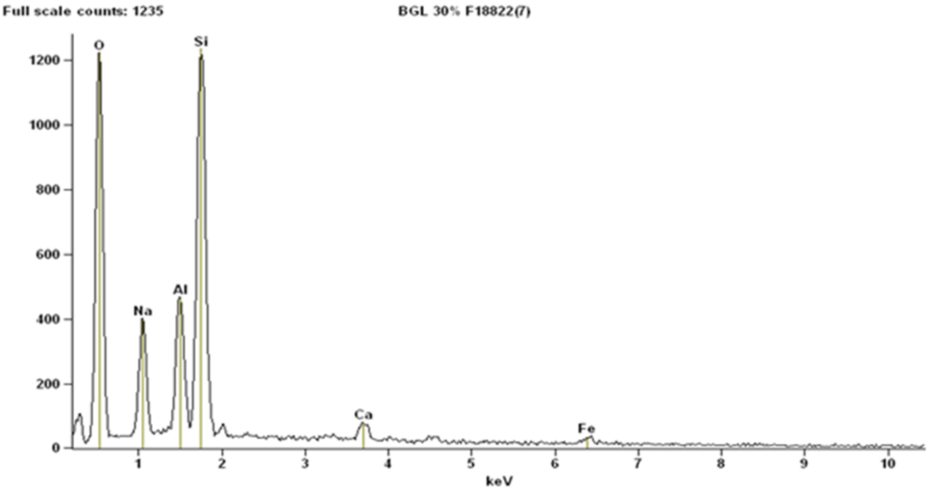

Element	Mass (g)	Element (%)
O	53.0 ± 0.7	65.8 ± 0.9
Na	12.3 ± 0.2	10.6 ± 0.2
Al	6.2 ± 0.2	4.6 ± 0.1
Si	24.6 ± 0.4	17.4 ± 0.3
Ca	1.9 ± 0.3	0.9 ± 0.1
Fe	1.9 ± 0.4	0.7 ± 0.1
Total	100.0	100.0

Fig. S12 SEM of the geopolymer with the addition of 30% ash from BA sewage sludge; Geopolymer EDS with the addition of 30% BA sewage sludge ash

*A, WB, PL: Crystallization points.

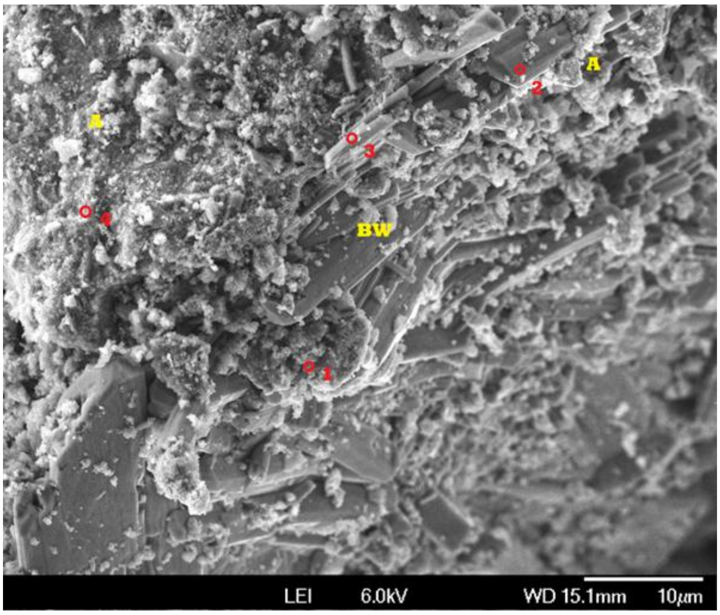

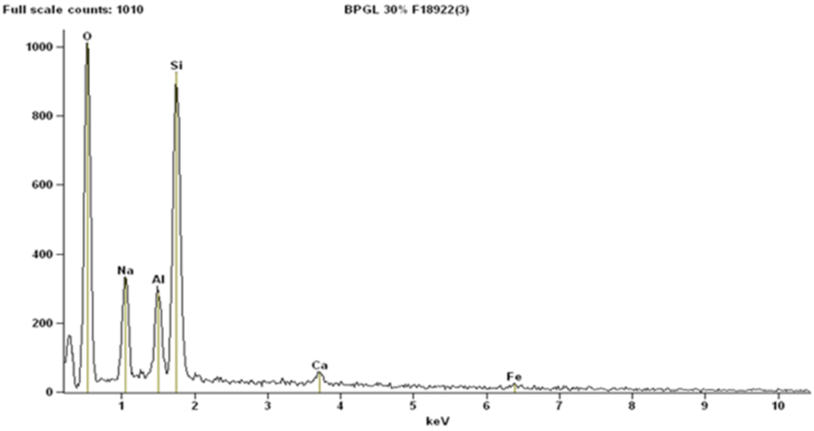

Element	Mass (g)	Element (%)
O	50.6 ± 0.6	63.9 ± 0.7
Na	11.1 ± 0.3	9.7 ± 0.3
Al	8.2 ± 0.3	6.2 ± 0.2
Si	25.3 ± 0.4	18.2 ± 0.3
Ca	2.2 ± 0.1	1.1 ± 0.1
Fe	2.5 ± 0.3	0.9 ± 0.1
Total	100.0	100.0

Fig. S13 SEM of the geopolymer with the addition of 30% ash from BP sewage sludge; Geopolymer EDS with the addition of 30% ash from BP sewage sludge.

*A, WB, PL: Crystallization points.

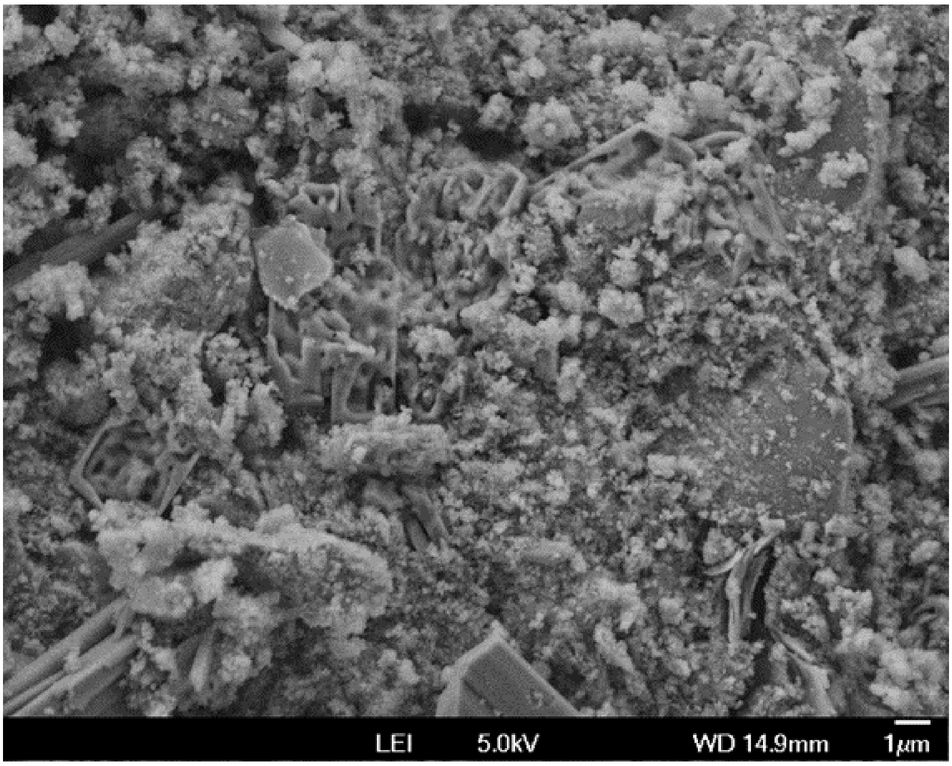

Fig. S14 SEM of the geopolymer with the addition of 10% of BA sewage sludge ash

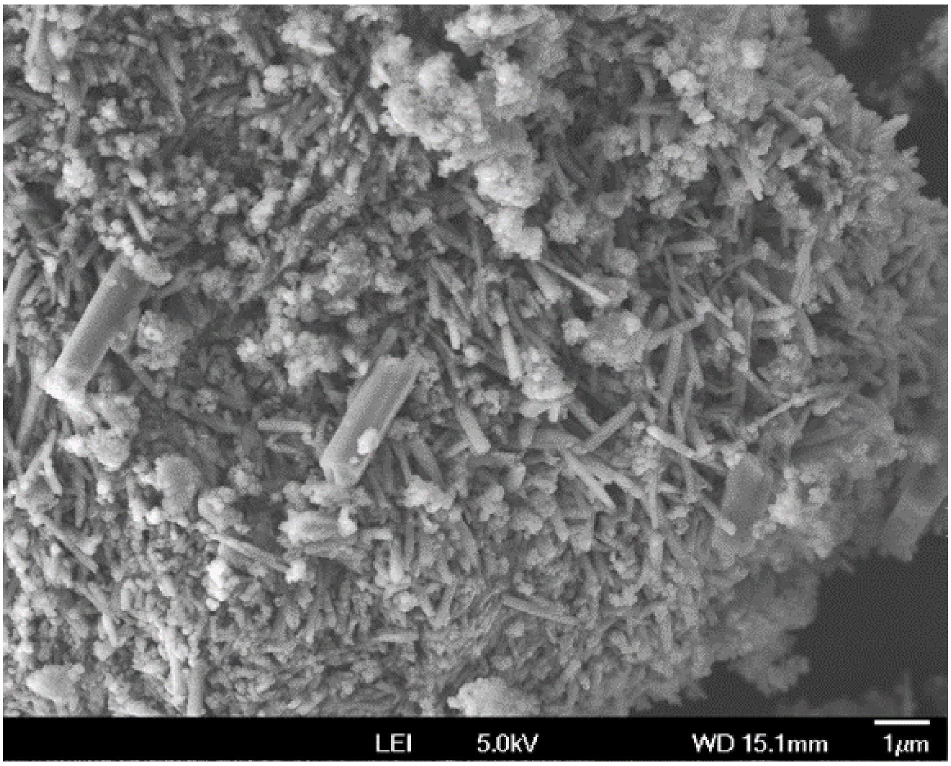

Fig. S15 – SEM of the geopolymer with the addition of 10% ash from sewage sludge from BP

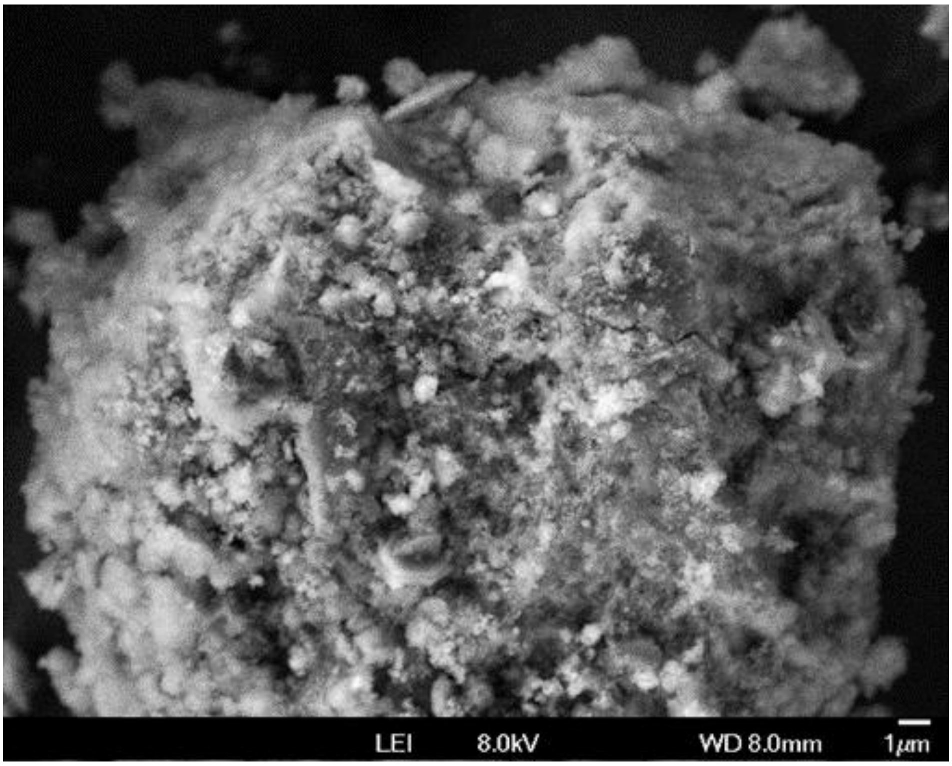

Fig. S16 SEM of the geopolymer with the addition of 30% ash from BA sewage sludge

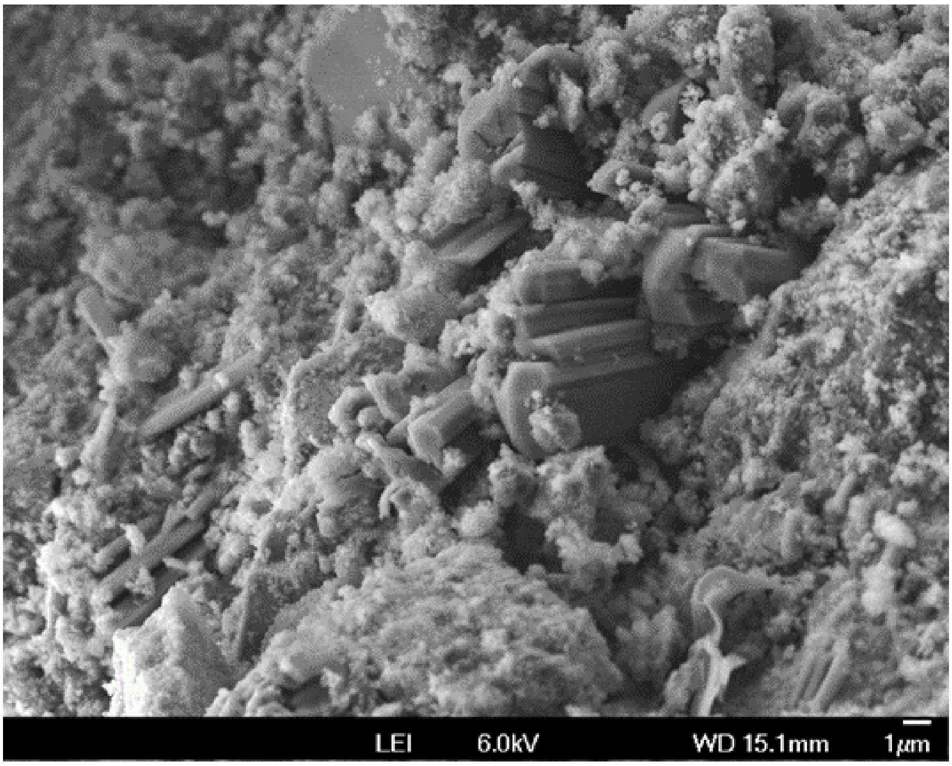

Fig. S17 SEM of the geopolymer with the addition of 30% ash from sewage sludge from BP
.

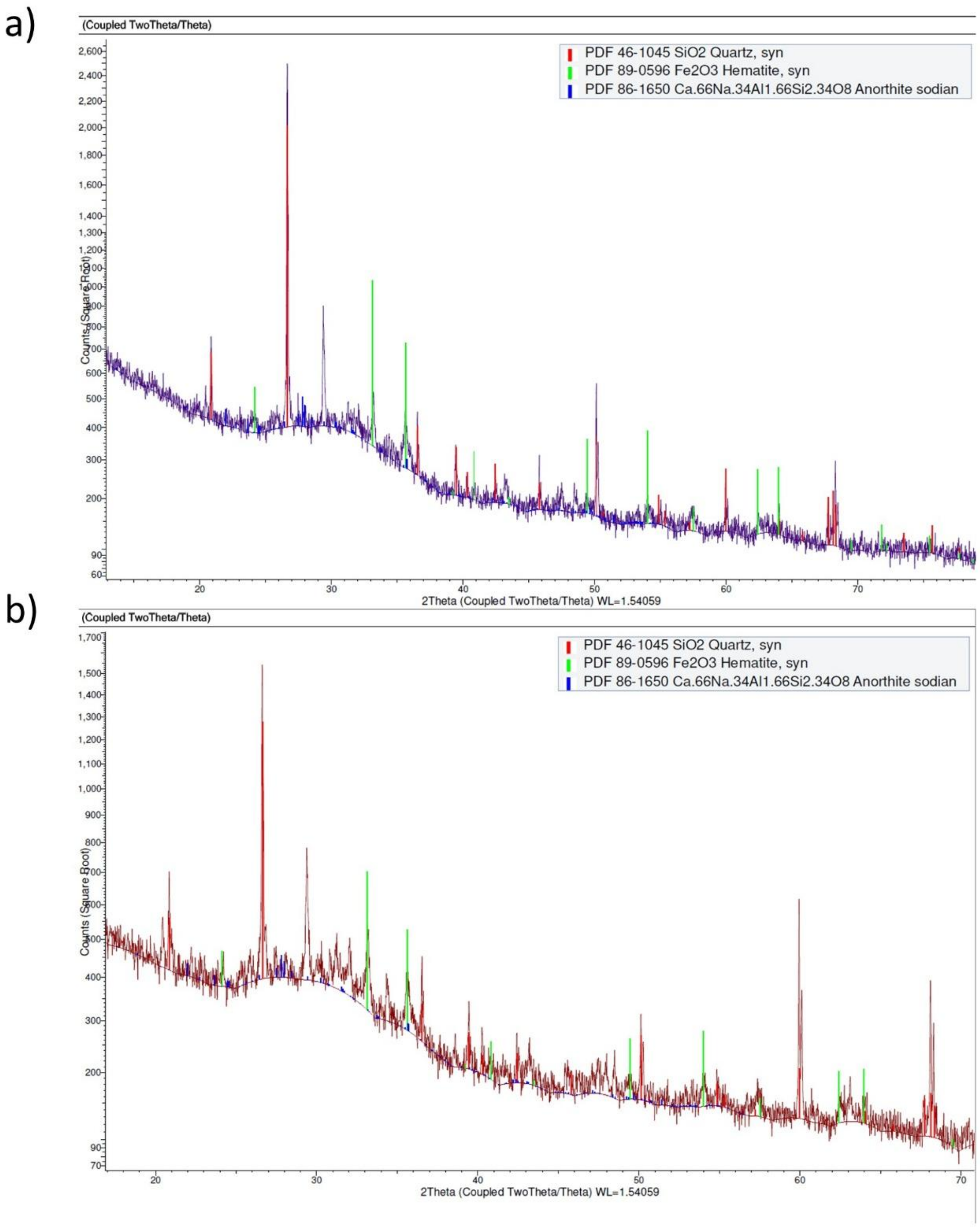

Fig. S18 Diffractogram of the geopolymers with a) simulated IER waste; b) simulated AC waste

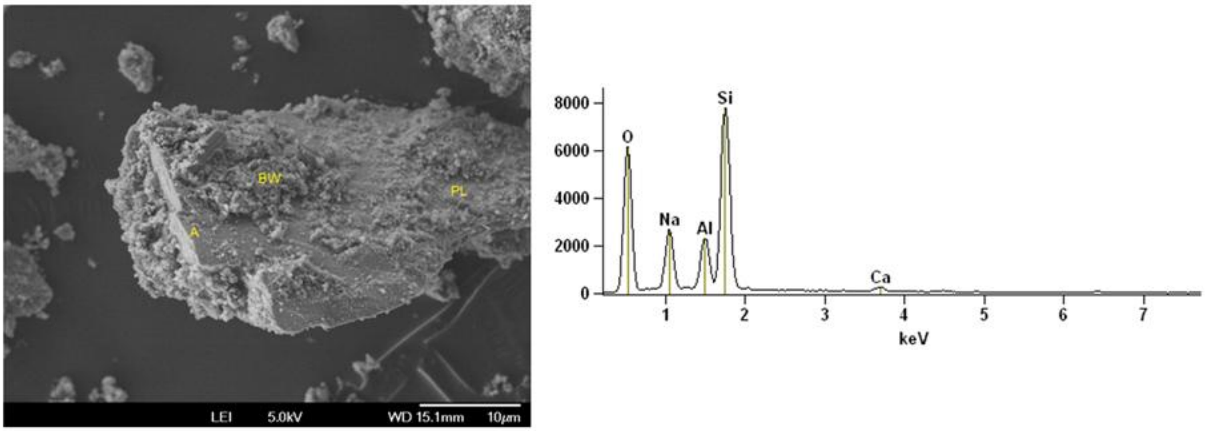

Fig. S19 SEM and EDS for Geopolymer with 10% addition of SSA – waste simulated with activated carbon

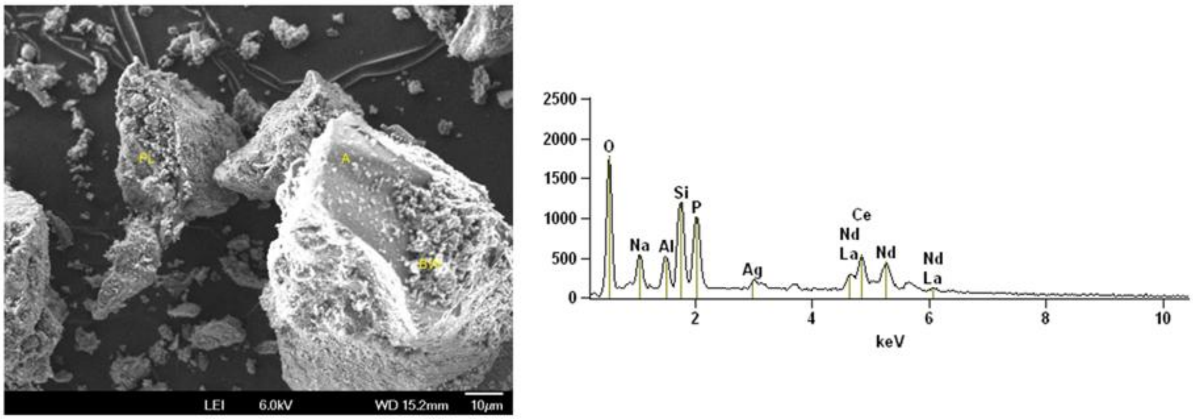

Fig. S20 SEM and EDS for Geopolymer with 30% addition of SSA – waste simulated with activated carbon

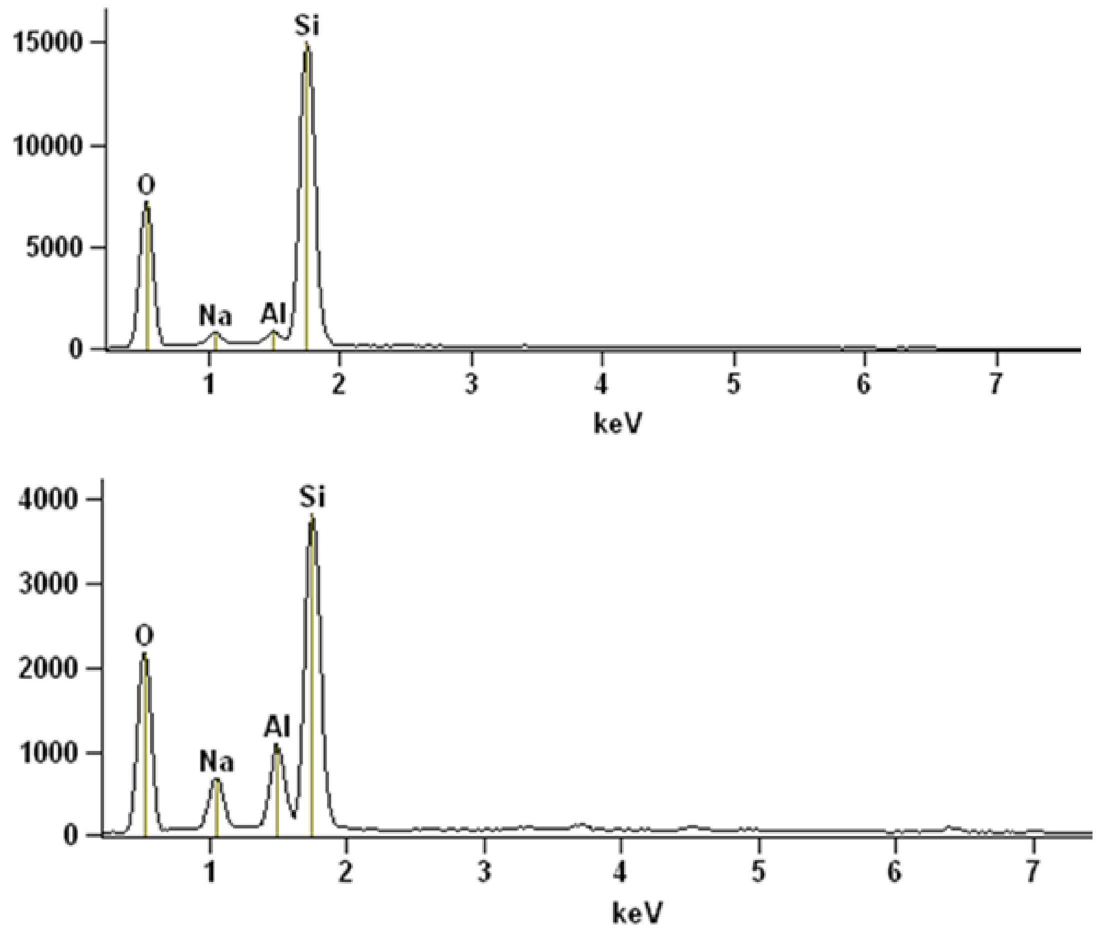

Fig. S21 EDS (upper) geopolymer with 10% addition of SSA – simulated waste with IER (down) geopolymer with 30% addition of SSA – simulated waste with IER

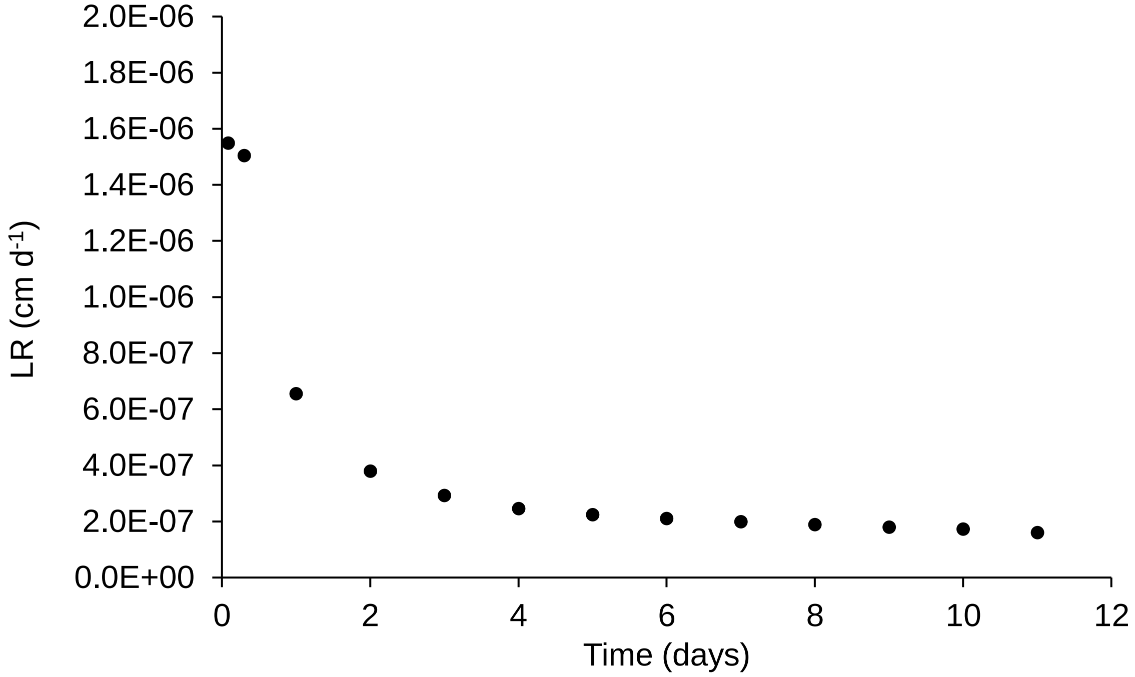

Fig. S22 Leaching rate (LR) of cesium in standard geopolymer

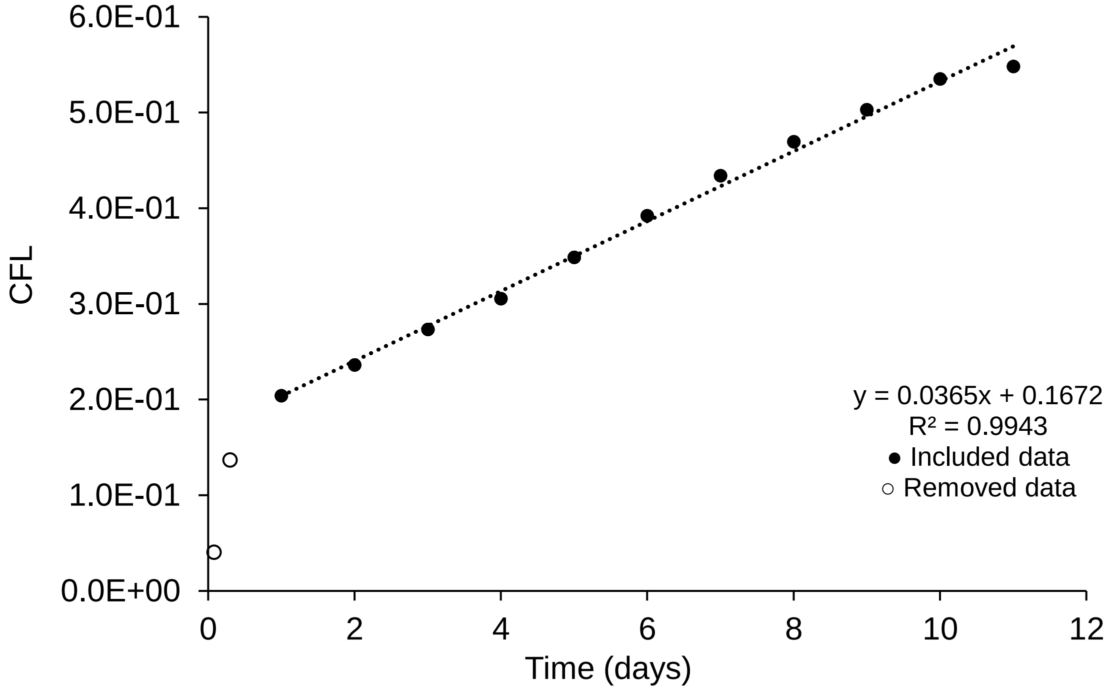

Fig. S23 Cumulative fraction of cesium leachate (FCL) into standard geopolymer

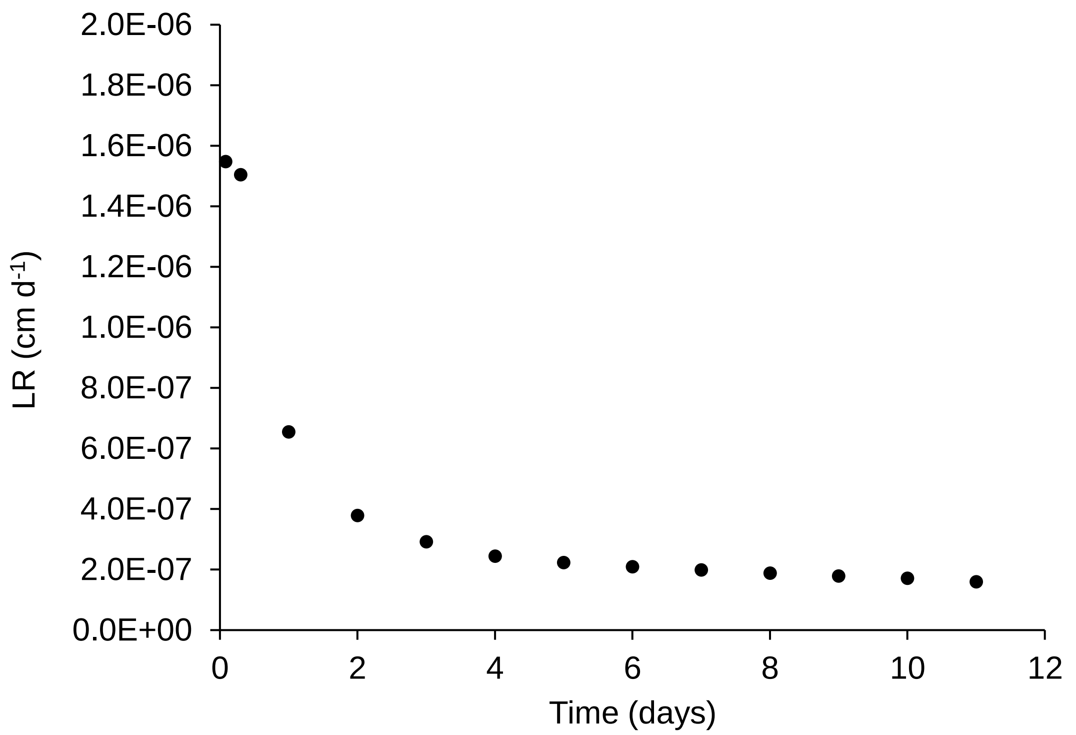

Fig. S24 Leaching rate (LR) of cesium in geopolymer with the addition of 10% SSA from BA

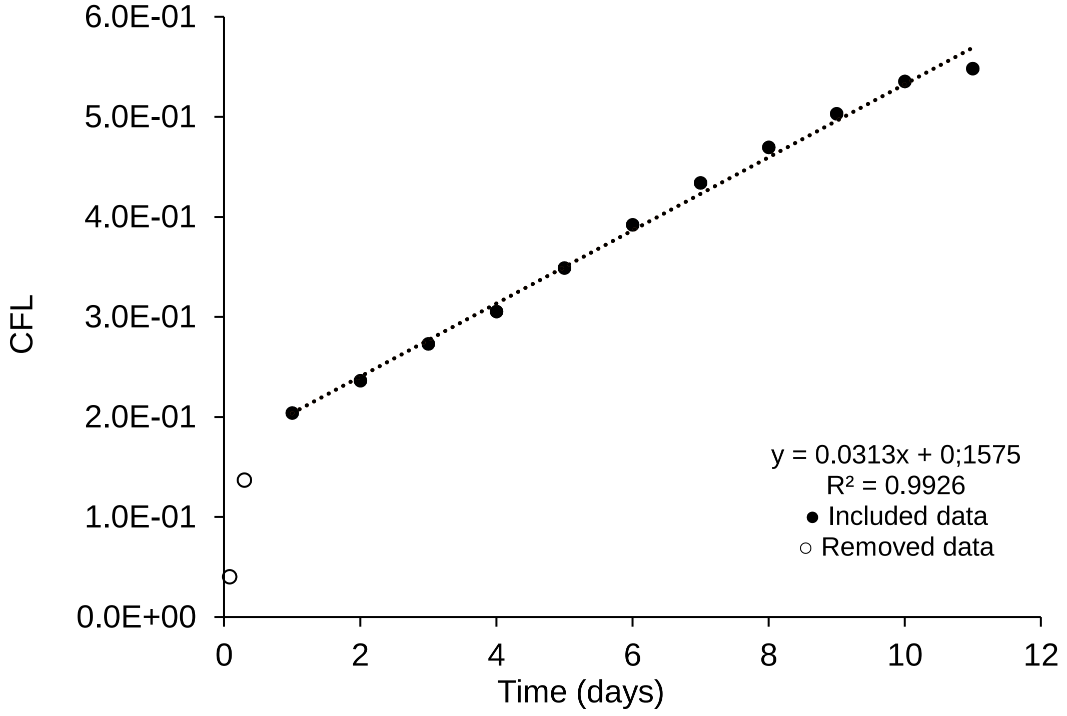

Fig. S25 Cumulative fraction of cesium leachate (FCL) in geopolymer with the addition of 10% SSA from BA.

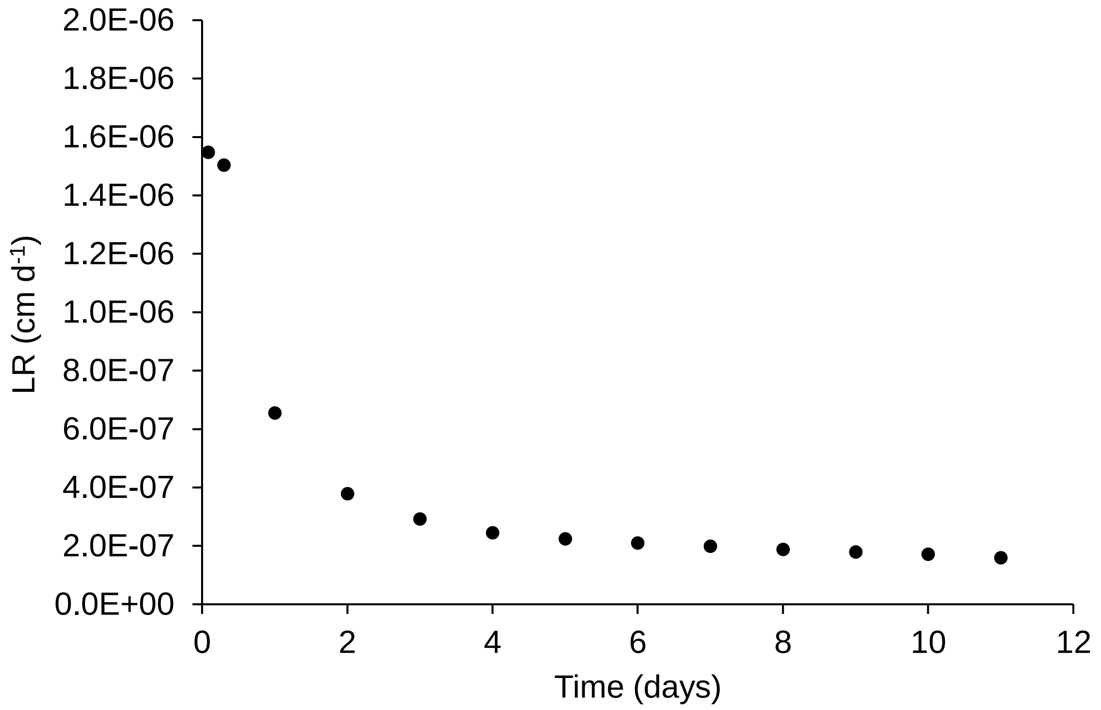

Fig. S26 Leaching rate (LR) of cesium in geopolymer with the addition of 30% SSA from BA

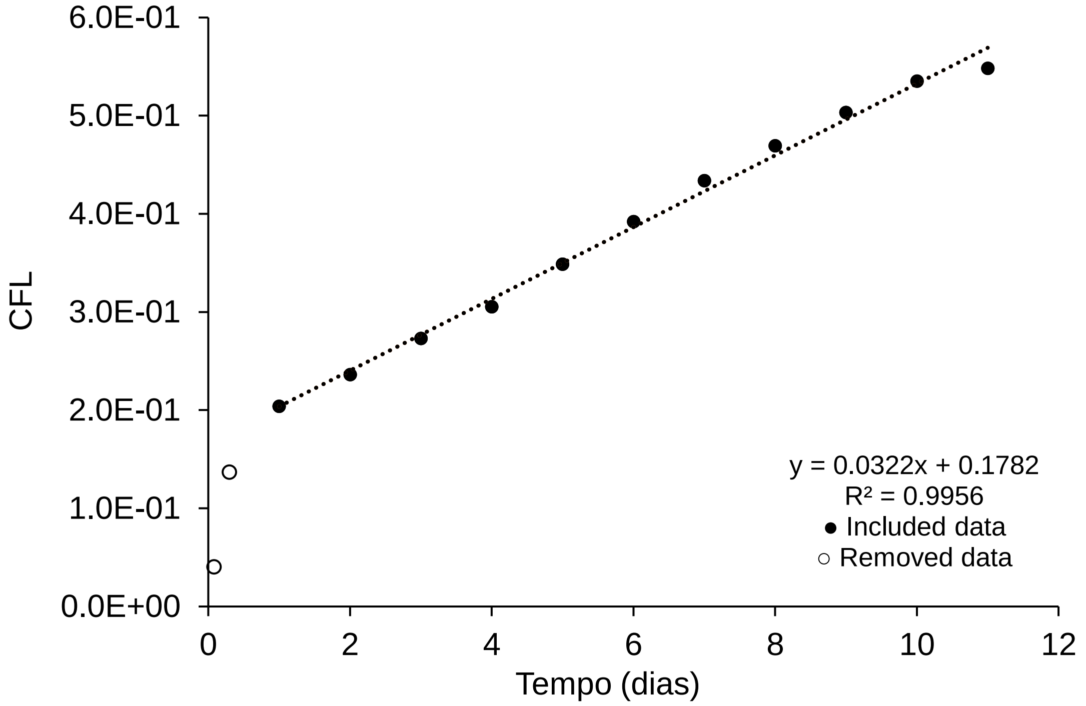

Fig. S27 Cumulative fraction of cesium leachate (FCL) in geopolymer with the addition of 30% SSA from BA.

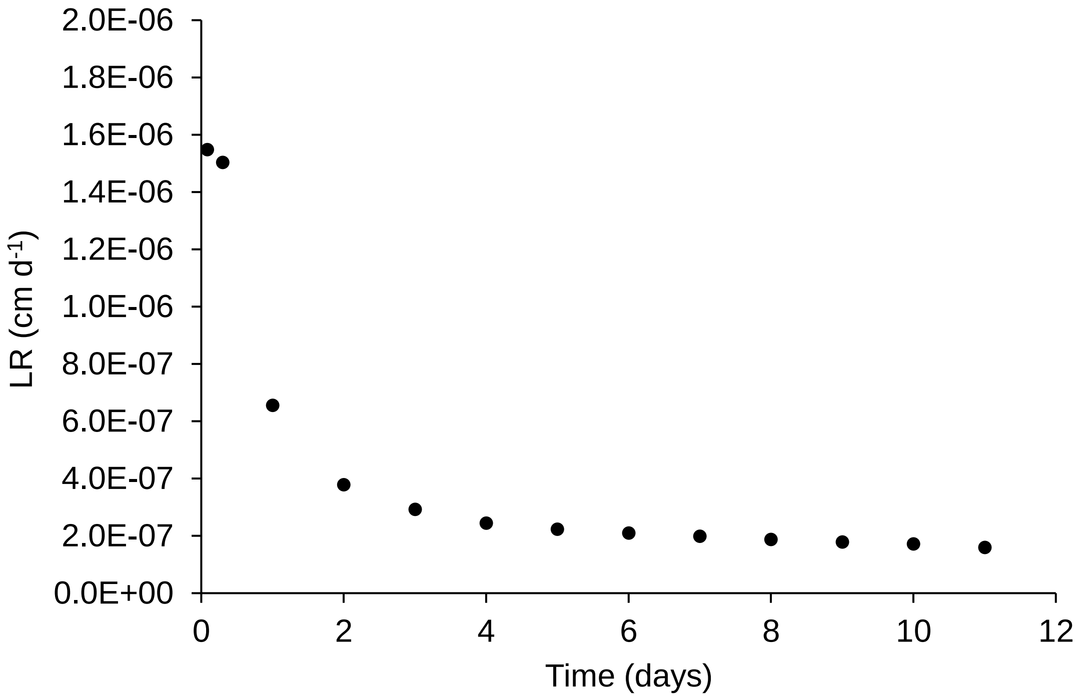

Fig. S28 Leaching rate (LR) of cesium in geopolymer with the addition of 10% SSA from BP

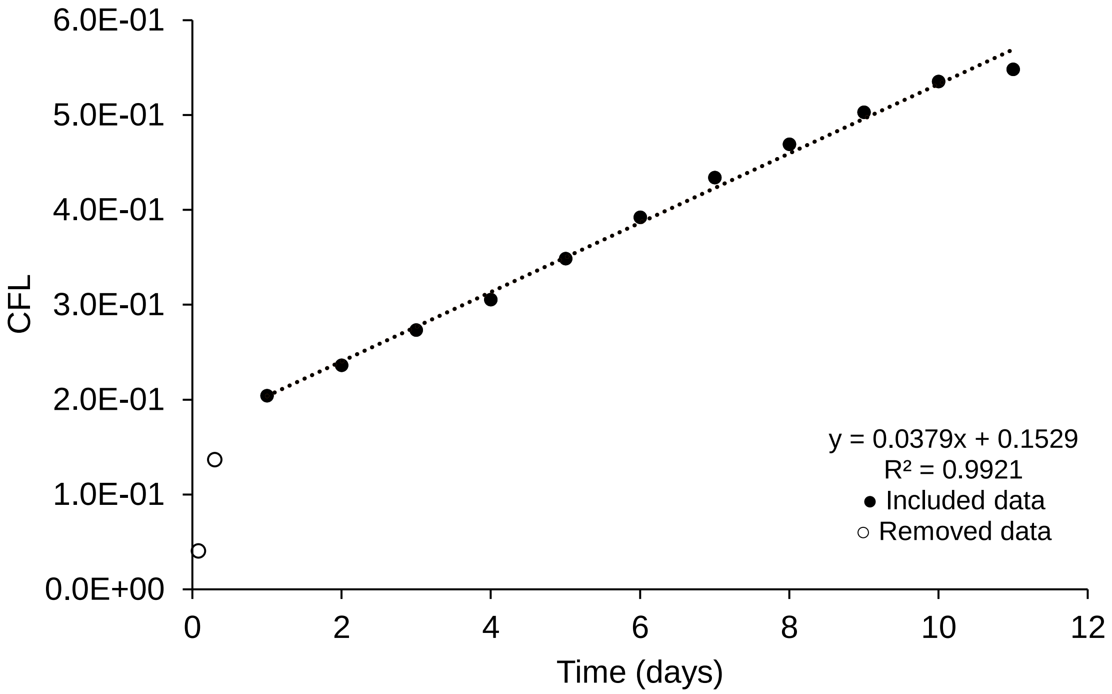

Fig. S29 Cumulative fraction of cesium leachate (FCL) in geopolymer with the addition of 10% SSA from BP.

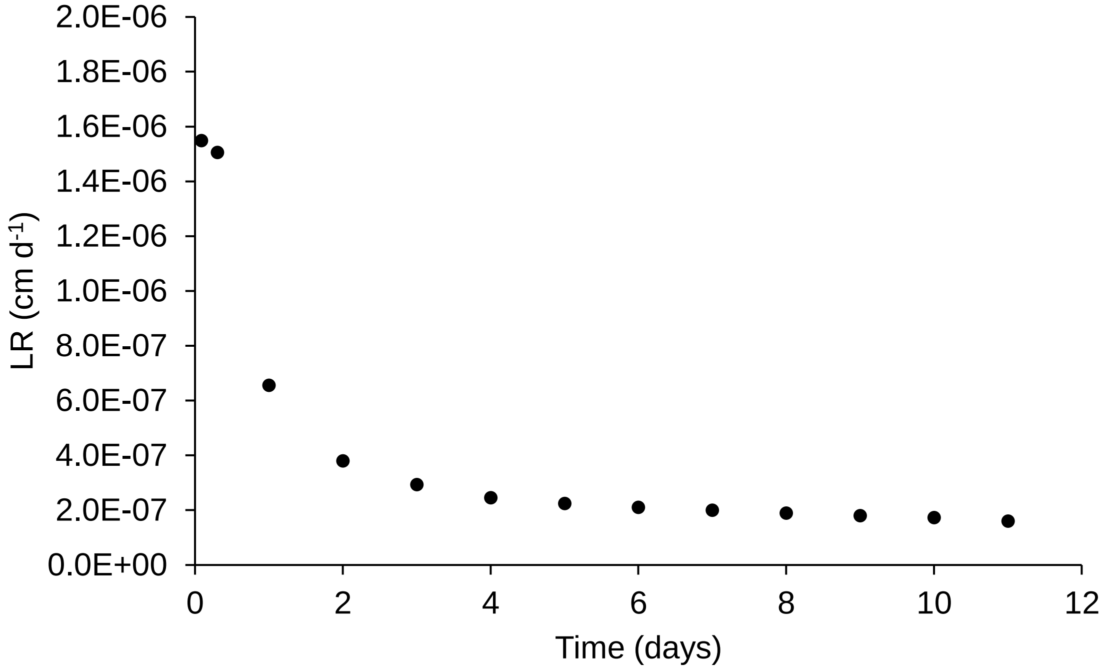

Fig. S30 Leaching rate (LR) of cesium in geopolymer with the addition of 30% SSA from BP

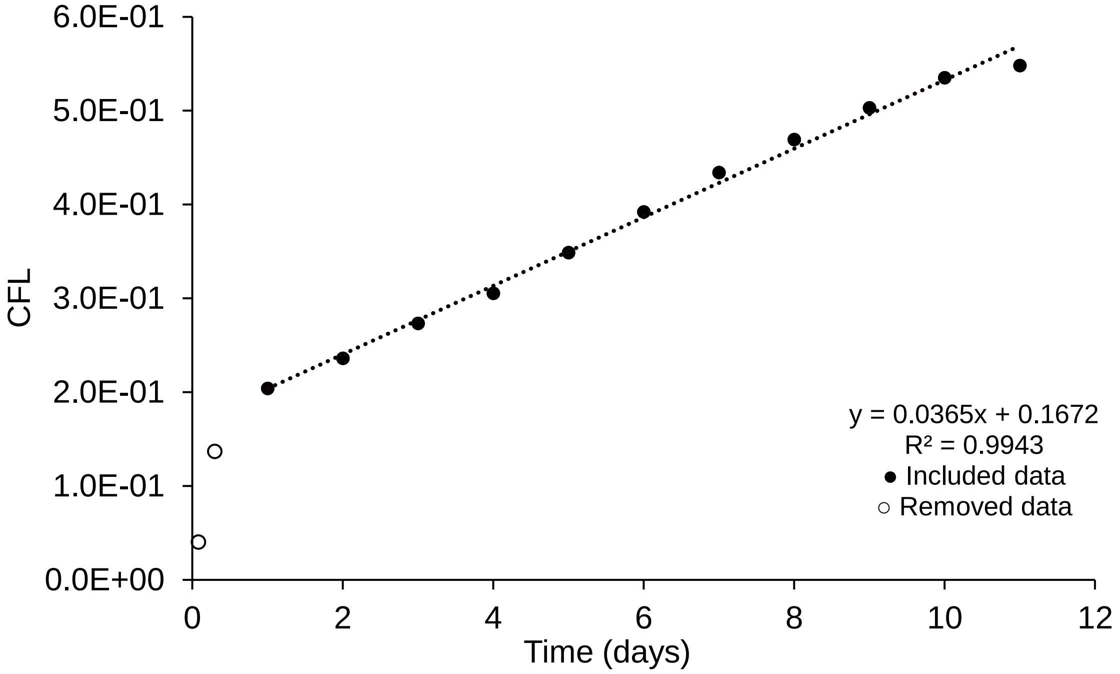

Fig. S31 Cumulative fraction of cesium leachate (FCL) in geopolymer with the addition of 30% SSA from BP.

Supporting References

[1] ABNT, NBR 10007/2004 - Amostragem de resíduos sólidos, (2004) 25.
[2] CNEN, Acceptance criteria for disposal of low- and intermediate-level radioactive waste - CNEN-NN-6.09. Resolução CNEN 012/02., (2002) 1–12.
[3] R Core Team, R: A language and environment for statistical computing. R Foundation for Statistical Computing, (2023). https://www.r-project.org/.
[4] K. Dunn, pid: Process Improvement using Data, (2018). https://cran.r-project.org/package=pid.
[5] Q. Li, Z. Sun, D. Tao, Y. Xu, P. Li, H. Cui, J. Zhai, Immobilization of simulated radionuclide 133Cs+ by fly ash-based geopolymer, Journal of Hazardous Materials 262 (2013) 325–331. https://doi.org/10.1016/j.jhazmat.2013.08.049.
[6] Z. Xu, Z. Jiang, D. Wu, X. Peng, Y. Xu, N. Li, Y. Qi, P. Li, Immobilization of strontium-loaded zeolite A by metakaolin based-geopolymer, Ceramics International 43 (2017) 4434–4439. https://doi.org/10.1016/j.ceramint.2016.12.092.
[7] R.A.A. Boca Santa, Desenvolvimento de geopolímeros a partir de cinzas pesadas oriundas da queima do carvão mineral e metacaulim sintetizado a partir de resíduo da indústria de papel, Universidade Federal de Santa Catarina, 2012.
[8] A. Fernández-Jiménez, A. Palomo, M. Criado, Microstructure development of alkali-activated fly ash cement: a descriptive model, Cement and Concrete Research 35 (2005) 1204–1209. https://doi.org/10.1016/j.cemconres.2004.08.021.

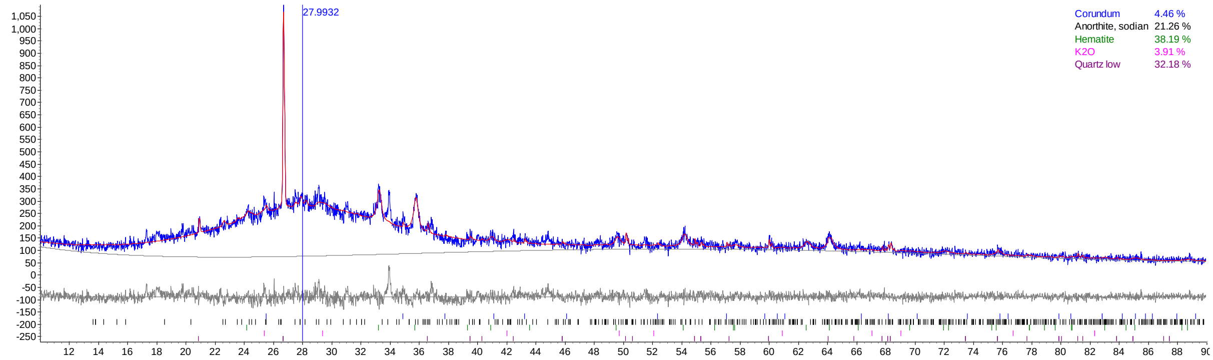

Fig. S32 Raw diffractogram of the SSA obtained from BA

Table S15 Analysis from the SSA obtained from BA

R-Values

Rexp : 8.38	Rwp : 9.92	Rp : 7.64	GOF : 1.18
Rexp`: 14.60	Rwp`: 17.28	Rp` : 14.93	DW : 1.49

Quantitative Analysis - Rietveld

Phase 1	: Corundum	4.462 %
Phase 2	: "Anorthite, sodian"	21.258 %
Phase 3	: Hematite	38.191 %
Phase 4	: K2O	3.908 %
Phase 5	: "Quartz low"	32.181 %

**Degree of crystallinity (%)**	14.64
Crystalline area	615.852
Amorphous area	3590.191

Background

One on X		643.3819
Chebychev polynomial, Coefficient	0	62.08081
	1	6.455612
	2	-21.44625
	3	-7.679536
	4	8.791109

Instrument

Primary radius (mm)	250
Secondary radius (mm)	250
Receiving slit width (mm)	0.3
FDS angle (°)	0.5563
Beam spill, Sample length (mm)	25
Intensity corrected	
Full Axial Convolution	
Filament length (mm)	16
Sample length (mm)	25
Receiving Slit length (mm)	16
Primary Sollers (°)	2.5
Secondary Sollers (°)	2.5

Corrections

Specimen displacement	-0.1194021
LP Factor	26.4

Table S16 Analysis from the SSA obtained from BA - Structures 1-5

Structure 1

Phase name	Corundum
R-Bragg	4.901
Spacegroup	R-3cH
Scale	3.01028e-006
Cell Mass	611.768
Cell Volume (Å^3)	258.46013
Wt% - Rietveld	4.462
Double-Voigt|Approach	
Cry size Lorentzian	9999.0
Cry size Gaussian	1000.0
k: 1 LVol-IB (nm)	852.800
k: 0.89 LVol-FWHM (nm)	845.413
Crystal Linear Absorption Coeff. (1/cm)	124.164
Crystal Density (g/cm^3)	3.930
Lattice parameters	
a (Å)	4.7614242
c (Å)	13.1640398

Site	Np	x	y	z	Atom	Occ	Beq
Al1	12	0.00000	0.00000	0.35228	Al+3	1	0
O1	18	0.30640	0.00000	0.25000	O-2	1	0

Structure 2

Phase name	Anorthite, sodian
R-Bragg	3.781
Spacegroup	C-1
Scale	3.14339e-006
Cell Mass	1084.057
Cell Volume (Å^3)	665.51840
Wt% - Rietveld	21.258
Double-Voigt|Approach	
Cry size Lorentzian	9979.5
Cry size Gaussian	47.6
k: 1 LVol-IB (nm)	44.467
k: 0.89 LVol-FWHM (nm)	42.212
Crystal Linear Absorption Coeff. (1/cm)	119.313
Crystal Density (g/cm^3)	2.705
Lattice parameters	
a (Å)	7.9651069
b (Å)	13.0359616
c (Å)	7.1306355
alpha (°)	94.54117
beta (°)	115.4901
gamma (°)	90.42319

Site	Np	x	y	z	Atom	Occ	Beq
Na1	4	0.26850	-0.02470	0.17010	Na+1	0.225	0
Na2	4	0.26920	0.02484	0.10290	Na+1	0.225	0
Ca1	4	0.26850	-0.02470	0.17010	Ca+2	0.275	0
Ca2	4	0.26920	0.02484	0.10290	Ca+2	0.275	0
Al1	4	0.00420	0.16670	0.21110	Al+3	0.32	1.21
Al2	4	0.00840	0.16330	0.21670	Al+3	0.32	1.22
Al3	4	0.00340	0.81710	0.23370	Al+3	0.16	0.98
Al4	4	0.00370	0.81890	0.23030	Al+3	0.16	1.05
Al5	4	0.68780	0.10990	0.31670	Al+3	0.12	0.65
Al6	4	0.68540	0.10970	0.31790	Al+3	0.16	1.01
Al7	4	0.68250	0.87990	0.35690	Al+3	0.12	0.77
Al8	4	0.68300	0.87980	0.35850	Al+3	0.19	0.95
Si1	4	0.00420	0.16670	0.21110	Si+4	0.18	1.21
Si2	4	0.00840	0.16330	0.21670	Si+4	0.18	1.22

Si3	4	0.00340	0.81710	0.23370	Si+4	0.34	0.98
Si4	4	0.00370	0.81890	0.23030	Si+4	0.34	1.05
Si5	4	0.68780	0.10990	0.31670	Si+4	0.38	0.65
Si6	4	0.68540	0.10970	0.31790	Si+4	0.34	1.01
Si7	4	0.68250	0.87990	0.35690	Si+4	0.38	0.77
Si8	4	0.68300	0.87980	0.35850	Si+4	0.31	0.95
O1	4	0.00700	0.12670	0.97880	O-2	0.5	1.72
O2	4	-0.00350	0.13300	0.97800	O-2	0.5	2.75
O3	4	0.57540	0.99520	0.28060	O-2	0.5	1.35
O4	4	0.58800	0.99070	0.27440	O-2	0.5	1.19
O5	4	0.81260	0.10360	0.18840	O-2	0.5	2.27
O6	4	0.81180	0.10840	0.18860	O-2	0.5	1.7
O7	4	0.81300	0.85330	0.23840	O-2	0.5	3
O8	4	0.82070	0.85250	0.25440	O-2	0.5	1.78
O9	4	0.51490	0.79640	0.27420	O-2	0.5	3.07
O10	4	0.51390	0.79120	0.28200	O-2	0.5	1.67
O11	4	0.51790	0.18680	0.22340	O-2	0.5	3.14
O12	4	0.51570	0.19220	0.21260	O-2	0.5	1.68
O13	4	0.19070	0.10880	0.39160	O-2	0.5	3.02
O14	4	0.20930	0.10740	0.37900	O-2	0.5	2.7
O15	4	0.19020	0.86950	0.42500	O-2	0.5	1.62
O16	4	0.18940	0.86500	0.43820	O-2	0.5	2.52

Structure 3

Phase name	Hematite
R-Bragg	1.091
Spacegroup	R-3cH
Scale	1.41141e-005
Cell Mass	958.129
Cell Volume (Å^3)	301.27371
Wt% - Rietveld	38.191
Double-Voigt|Approach	
Cry size Lorentzian	29.8
Cry size Gaussian	1000.0
k: 1 LVol-IB (nm)	19.073
k: 0.89 LVol-FWHM (nm)	26.595
Crystal Linear Absorption Coeff. (1/cm)	1131.511
Crystal Density (g/cm^3)	5.281
Preferred Orientation Spherical Harmonics	
Order	8
y00	1
y20	-0.4071335
y40	0.2178824
y43m	0.05061979
y60	-0.7987442
y63m	0.03583798
y66p	0.1429219
y80	-0.2097357
y83m	-0.1606211
y86p	-0.007588159
Lattice parameters	
a (Å)	5.0294112
c (Å)	13.7529643

Site	Np	x	y	z	Atom	Occ	Beq
Fe1	12	0.00000	0.00000	0.35520	Fe+3	1	0
O1	18	0.69418	0.00000	0.25000	O-2	1	0

Structure 4

Phase name	K2O
R-Bragg	2.368
Spacegroup	Fm-3m
Scale	9.83900e-006
Cell Mass	188.392
Cell Volume (Å^3)	224.93482
Wt% - Rietveld	3.908
Double-Voigt|Approach	
Cry size Lorentzian	9999.0
Cry size Gaussian	19.9
k: 1 LVol-IB (nm)	18.651
k: 0.89 LVol-FWHM (nm)	17.685
Crystal Linear Absorption Coeff. (1/cm)	169.759
Crystal Density (g/cm^3)	1.391
Lattice parameters	
a (Å)	6.0816146

Site	Np	x	y	z	Atom	Occ	Beq
K1	4	0.00000	0.00000	0.00000	K+1	1	0
O1	4	0.50000	0.50000	0.50000	O-2	0.5	0

Structure 5

Phase name	Quartz low
R-Bragg	1.025
Spacegroup	P3221
Scale	1.68376e-004
Cell Mass	180.253
Cell Volume (Å^3)	113.11249
Wt% - Rietveld	32.181
Double-Voigt|Approach	
Cry size Lorentzian	870.4
Cry size Gaussian	317.8
k: 1 LVol-IB (nm)	217.263
k: 0.89 LVol-FWHM (nm)	233.507
Crystal Linear Absorption Coeff. (1/cm)	94.767
Crystal Density (g/cm^3)	2.646
Preferred Orientation Spherical Harmonics	
Order	8
y00	1
y20	0.3900744
y40	-0.1563357
y43m	-0.8996982
y60	-0.6346874
y63m	0.7988758
y66p	-0.0036836
y80	-0.3002728
y83m	0.6122324
y86p	-0.3472779
Lattice parameters	
a (Å)	4.9145345
c (Å)	5.4077317

Site	Np	x	y	z	Atom	Occ	Beq
Si1	3	0.47230	0.00000	0.66667	Si+4	1	0.793
O1	6	0.41600	0.26580	0.78810	O-2	1	1.479

Table S17 Analysis from the SSA obtained from BA - Peaks

Peaks Phase 1

Phase name	Peaks Phase:0

Type	Position	I		
FP	27.9932	143.4269	Cry size Lor	0.8
			Cry size Gauss	1.5
			k: 1, LVol-IB(nm)	0.455
			k: 0.89, LVol-FWHM(nm)	0.589

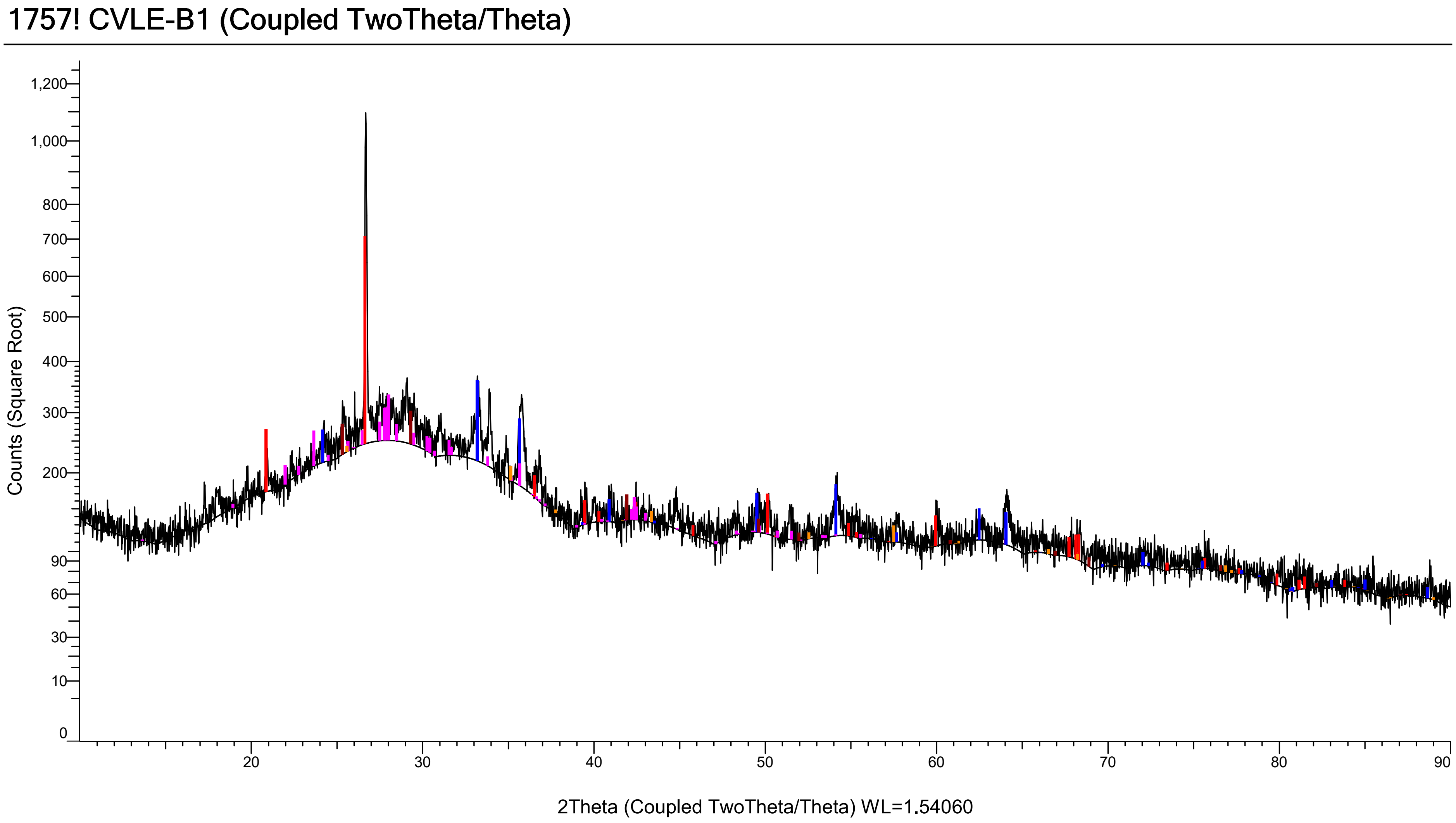

Fig. S33 High definition diffractogram of the SSA obtained from BA

Table S18 Patterns from the SSA obtained from BA. Pattern: PDF 78-2315, Radiation: 1.54060

Formula	SiO2
Name	Silicon Oxide
Name (mineral)	Quartz
Name (common)	
Status	Primary
Ambient	Yes

Lattice:	Hexagonal	Mol. weight =	60.08
S.G.:	P3221 (154)	Volume [CD] =	112.93
		Dx =	
		Dm =	
		I/Icor =	3.100

a =	4.91239	Z =	3
c =	5.40385		
a/b =	1.00000		
c/b =	1.10004		

ICSD Collection Code: 063532
Additional Pattern: See PDF 87-2096, PDF 85-335, PDF 85-457, PDF 85-504, PDF 85-695, PDF 85-794, PDF 85-795, PDF 85-796, PDF 85-797, PDF 85-798, PDF 85-930, PDF 85-1053, PDF 85-1054, PDF 85-1780, PDF 86-1560 and PDF 86-1561
Article Title: Crystal structures of quartz and magnesium germanate by profile analysis of synchrotron-radiation high-resolution powder data

Structure
Publication: J. Appl. Crystallogr.
Detail: volume 21, page 182 (1988)
Authors: Will, G., Bellotto, M., Parrish, W., Hart, M.
Primary Reference
Publication: Calculated from ICSD using POWD-12++

Radiation:	CuKa1	Filter:	Not specified
Wavelength:	1.54060	d-spacing:	
SS/FOM:	F(29)= 999.9 (0.0001, 29)		

d	2θ	I fix	h	k	l
4.25425	20.864	208	1	0	0
3.34268	26.646	999	0	1	1
2.45620	36.554	68	1	1	0
2.28080	39.478	65	1	0	2
2.23605	40.302	32	1	1	1
2.12713	42.462	49	2	0	0
1.97930	45.807	28	2	0	1
1.81747	50.153	110	1	1	2
1.80128	50.636	4	0	0	3
1.67134	54.889	34	0	2	2
1.65873	55.342	15	0	1	3
1.60796	57.247	2	2	1	0
1.54118	59.975	84	1	2	1
1.45254	64.053	14	1	1	3
1.41808	65.803	4	3	0	0
1.38178	67.762	46	1	2	2
1.37463	68.163	59	2	0	3
1.37164	68.332	62	0	3	1
1.28760	73.489	18	1	0	4
1.25565	75.681	26	3	0	2
1.22810	77.692	14	2	2	0
1.19955	79.906	25	2	1	3
1.19756	80.065	14	2	2	1
1.18372	81.196	20	1	1	4
1.17992	81.512	26	3	1	0
1.15276	83.860	15	1	3	1
1.14040	84.980	2	2	0	4
1.11803	87.099	1	2	2	2
1.11423	87.471	2	3	0	3

Table S19 Patterns from the SSA obtained from BA. Pattern: PDF 79-1910, Radiation: 1.54060

Formula	SiO2
Name	Silicon Oxide
Name (mineral)	Quartz
Name (common)	
Status	Primary
Ambient	Yes

Lattice:	Hexagonal	Mol. weight =	60.08
S.G.:	P3121 (152)	Volume [CD] =	113.05
		Dx =	
		Dm =	
		I/Icor =	3.070

a =	4.91400	
c =	5.40600	Z = 3
a/b =	1.00000	
c/b =	1.10012	

ICSD Collection Code: 067121
Remark From ICSD/CSD: REM K Isotropic temperature factors obtained from room press
Remark From ICSD/CSD: REM refinement. Refinement was constrained to have a twin fr
Remark From ICSD/CSD: REM of .035+3.
Temperature Factor: ITF
Remark From ICSD/CSD: REM PRE Mentioned
Article Title: High-pressure crystal chemistry and amorphization of alpha quartz

Structure
Publication: Solid State Commun.
Detail: volume 72, page 507 (1989)
Authors: Hazen, R.M., Finger, L.W., Hemley, R.J., Mao, H.K.
Primary Reference
Publication: Calculated from ICSD using POWD-12++

Radiation:	CuKa1	Filter:	Not specified
Wavelength:	1.54060	d-spacing:	
SS/FOM:	F(28)= 999.9 (0.0001, 29)		

d	2θ	I fix	h	k	l
4.25565	20.857	210	1	0	0
3.34387	26.637	999	1	0	1
2.45700	36.542	67	1	1	0
2.28166	39.462	66	0	1	2
2.23681	40.287	29	1	1	1
2.12782	42.448	49	2	0	0
1.97997	45.790	27	0	2	1
1.81812	50.134	110	1	1	2
1.80200	50.614	4	0	0	3
1.67193	54.868	33	2	0	2
1.65937	55.319	14	1	0	3
1.60848	57.227	2	2	1	0
1.54169	59.953	77	2	1	1
1.45309	64.026	14	1	1	3
1.41855	65.779	4	3	0	0
1.38226	67.735	46	2	1	2
1.37513	68.134	57	0	2	3
1.37210	68.306	60	3	0	1
1.28810	73.455	17	0	1	4
1.25608	75.651	22	0	3	2
1.22850	77.662	12	2	2	0
1.19998	79.871	23	1	2	3
1.18418	81.157	19	1	1	4
1.18030	81.480	23	3	1	0
1.15314	83.826	14	3	1	1
1.14083	84.941	2	0	2	4
1.11841	87.062	1	2	2	2
1.11462	87.433	2	0	3	3

Table S20 Patterns from the SSA obtained from BA. Pattern: PDF 85-0599, Radiation: 1.54060

Formula	Fe_2O_3
Name	Iron Oxide
Name (mineral)	Hematite, syn
Name (common)	
Status	Primary
Ambient	Yes

Lattice:	Rhombo.H.axes	Mol. weight =	159.69
S.G.:	R-3c (167)	Volume [CD] =	301.15
		Dx =	
		Dm =	
		I/Icor =	3.090

a =	5.03200
c =	13.73300
a/b =	1.00000
c/b =	2.72913
Z =	6

ICSD Collection Code: 082137
Remark From ICSD/CSD: REM XDP
Remark From ICSD/CSD: REM K Sample calcinated at 1073 K
Temperature Factor: ITF
Article Title: Effect of mechanical activation on the real structure and reactivity of iron(III) oxide with corundum-type structure

Structure
Publication: J. Solid State Chem.
Detail: volume 123, page 191 (1996)
Authors: Sadykov, V.A., Isupova, L.A., Tsybulya, S.V., Cherepanova, S.V., Litvak, G.S., Burgina, E.B., Kustova, G.N., Kolomiichuk, V.N., Ivanov, V.P., Paukshtis, E.A., Golovin, A.V., Avvakumov, E.G.
Primary Reference
Publication: Calculated from ICSD using POWD-12++

Radiation:	CuKa1	Filter:	Not specified
Wavelength:	1.54060	d-spacing:	
SS/FOM:	F(28)= 999.9 (0.0001, 29)		

d	2θ	I fix	h	k	l
3.67939	24.169	375	0	1	2
2.69685	33.193	1000	1	0	4
2.51600	35.656	750	1	1	0
2.28883	39.333	26	0	0	6
2.20491	40.896	203	1	1	3
2.07686	43.542	23	2	0	2
1.83970	49.506	348	0	2	4
1.69308	54.126	460	1	1	6
1.63539	56.201	7	2	1	1
1.60167	57.493	27	1	2	2
1.59717	57.670	75	0	1	8
1.48505	62.491	265	2	1	4
1.45261	64.050	267	3	0	0
1.41258	66.092	4	1	2	5
1.34842	69.676	20	2	0	8
1.30980	72.045	95	1	0	10
1.30470	72.371	24	1	1	9
1.26147	75.271	4	2	1	7
1.25800	75.515	56	2	2	0
1.22646	77.815	24	0	3	6
1.21303	78.843	11	2	2	3
1.20399	79.552	1	1	3	1
1.19035	80.650	16	3	1	2
1.18850	80.801	29	1	2	8
1.16180	83.062	46	0	2	10
1.14442	84.612	4	0	0	12
1.14006	85.012	63	1	3	4
1.10245	88.648	65	2	2	6

Table S21 Patterns from the SSA obtained from BA. Pattern: PDF 18-1202, Radiation: 1.54060

Formula	(Ca,Na)(Si,Al)4O8
Name	Sodium Calcium Aluminum Silicate
Name (mineral)	Anorthite, sodian, intermediate
Name (common)	
Status	Primary
Ambient	Yes

Lattice:	Triclinic	Mol. weight =	280.42
S.G.:	C-1 (2)	Volume [CD] =	334.56
		Dx =	
		Dm =	2.72
		I/Icor =	-1.000

a =	8.17600	alpha =	93.450
b =	12.86500	beta =	116.100
c =	7.10200	gamma =	90.500
a/b =	0.63552	Z =	4
c/b =	0.55204		

Optical Data: A=1.5625, B=1.5668, Q=1.5718, Sign=+, 2V=85°
Sample Source Or Locality: Specimen from Lake County, Oregon, USA
General Comments: Pattern obtained at 26 C using F2tandard with a=5.4622, average of 3 patterns
Analysis: Chemical analysis reports anorthite 67.2, albite 31.5, orthoclase 1.3
Color: Colorless, light yellow
Additional Pattern: See ICSD 9287 (PDF 71-748); See ICSD 201648 (PDF 86-1650); See ICSD 62806 (PDF 78-1629)
Structure: Structural state intermediate between high and low but more similar to high
Unit Cell: Unit cell sub-cell indexes all powder reflections. True cell is a=8.17, b=12.87, c=14.19, α=93.29, β=116.0, γ=90.77

Primary Reference
Publication: Am. Mineral.
Detail: volume 51, page 177 (1966)
Authors: Stewart, Walker, Wright, Fahey.

Radiation:	CuKa	Filter:	F
Wavelength:	1.54060	d-spacing:	
SS/FOM:	F(30)= 34.0 (0.0136, 65)		

d	2θ	I fix	h	k	l
6.49000	13.633	2	-1	1	0
4.68500	18.927	6	0	-2	1
4.04200	21.973	35	-2	0	1
3.90400	22.759	16	1	-1	1
3.75900	23.650	70	-1	3	0
3.63200	24.489	12	1	3	0
3.47100	25.644	20	-1	-1	2
3.42600	25.987	2	-2	-2	1
3.36500	26.466	31	-1	1	2
3.24100	27.499	40	-2	2	0
3.21000	27.769	70	0	4	0
3.20300	27.831	70	-2	0	2
3.18100	28.028	100	0	0	2
3.13200	28.475	35	2	2	0
3.02700	29.485	25	1	-3	1
2.95100	30.262	31	0	-4	1
2.93600	30.421	31	0	-2	2
2.91000	30.699	10	-2	-2	2
2.83400	31.544	31	1	3	1
2.82200	31.681	16	-2	2	2
2.65000	33.797	18	-1	3	2
2.54700	35.208	6	2	-2	1
2.51500	35.671	40	-2	-4	1
2.46500	36.419	2	-2	4	0
2.45000	36.650	4	-1	5	0
2.43900	36.821	4	2	2	1
2.41800	37.153	4	-3	1	0
2.30800	38.993	4	-3	3	1
2.28200	39.456	2	-3	-3	1
2.22800	40.453	4	1	-5	1
2.21000	40.798	2	-3	-3	2
2.15800	41.826	2	-2	2	3
2.14000	42.195	16	-1	-5	2
2.13200	42.361	35	2	-4	1
2.12600	42.486	20	0	0	3
2.10100	43.016	12	1	5	1
2.01900	44.856	2	-4	0	2
1.92700	47.124	4	-4	2	2
1.88200	48.322	6	-3	3	3
1.87900	48.404	2	-3	5	1
1.84800	49.269	2	-4	0	3
1.84400	49.383	2	-2	4	3
1.83500	49.642	10	4	0	0
1.83400	49.671	2	1	-1	3
1.81900	50.108	2	2	6	0
1.79900	50.705	10	-3	5	2
1.77200	51.533	12	-2	0	4
1.75500	52.070	2	2	-4	2
1.71600	53.345	4	0	4	3
1.71100	53.514	4	1	-7	1

1.69300	54.129	2	-4	-4	1
1.65300	55.550	6	2	4	2

Table S22 Patterns from the SSA obtained from BA. Pattern: PDF 77-2176, Radiation: 1.54060

Formula	K2O
Name	Potassium Oxide
Name (mineral)	
Name (common)	
Status	Primary
Ambient	Yes

Lattice:	Cubic	Mol. weight =	94.2
S.G.:	Fm-3m (225)	Volume [CD] =	225.87
		Dx =	
		Dm =	
		I/Icor =	3.910

a =	6.09000
a/b =	1.00000
c/b =	1.00000
Z =	2

ICSD Collection Code: 060489
Remark From ICSD/CSD: REM F DIS O2(with disorderly orientation in halide posit
Test From ICSD: No R value given
Test From ICSD: At least one TF missing
Remark From ICSD/CSD: REM TEM Mentioned
Article Title: A High-Temperature Crystal Modification of K2 O

Structure
Publication: Acta Crystallogr.
Detail: volume 5, page 851 (1952)
Authors: Carter, G.F., Margrave, J.L., Templeton, D.H.
Primary Reference
Publication: Calculated from ICSD using POWD-12++

Radiation:	CuKa1	Filter:	Not specified
Wavelength:	1.54060	d-spacing:	
SS/FOM:	F(10)= 999.9 (0.0002, 10)		

d	2θ	I fix	h	k	l
3.51606	25.310	899	1	1	1
3.04500	29.307	1000	2	0	0
2.15314	41.925	582	2	2	0
1.83620	49.607	288	3	1	1
1.75803	51.973	168	2	2	2
1.52250	60.788	66	4	0	0
1.39714	66.918	90	3	3	1
1.36177	68.896	155	4	2	0
1.24312	76.581	103	4	2	2
1.17202	82.180	56	5	1	1

Table S23 Patterns from the SSA obtained from BA. Pattern: PDF 46-1212, Radiation: 1.54060

Formula	Al2O3
Name	Aluminum Oxide
Name (mineral)	Corundum, syn
Name (common)	
Status	Primary
Ambient	Yes

Lattice:	Rhombo.H.axes	Mol. weight =	101.96
S.G.:	R-3c (167)	Volume [CD] =	254.81
		Dx =	
		Dm =	
		I/Icor =	-1.000

a =	4.75870	Z =	6
c =	12.99290		
a/b =	1.00000		
c/b =	2.73035		

Sample Source Or Locality: The sample is an alumina plate as received from ICDD
General Comments: Unit cell computed from d#o#b#s
Optical Data: A=1.7604, B=1.7686, Sign=-

Structure
Publication: Acta Crystallogr., Sec. B: Structural Science
Detail: volume 49, page 973 (1993)
Primary Reference
Publication: Adv. X-Ray Anal.
Detail: volume 33, page 295 (1990)
Authors: Huang, T., Parrish, W., Masciocchi, N., Wang, P.

Radiation:	CuKa1	Filter:	Not specified
Wavelength:	1.54060	d-spacing:	
SS/FOM:	F(25)= 357.4 (0.0028, 25)		

d	2θ	I fix	h	k	l
3.47975	25.579	45	0	1	2
2.55085	35.153	100	1	0	4
2.37947	37.777	21	1	1	0
2.16542	41.676	2	0	0	6
2.08532	43.356	66	1	1	3
1.96432	46.176	1	2	0	2
1.74007	52.550	34	0	2	4
1.60156	57.497	89	1	1	6
1.54667	59.741	1	2	1	1
1.51506	61.118	2	1	2	2
1.51101	61.300	14	0	1	8
1.40452	66.521	23	2	1	4
1.37372	68.214	27	3	0	0
1.33599	70.420	1	1	2	5
1.27555	74.299	2	2	0	8
1.23915	76.871	29	1	0	10
1.23434	77.226	12	1	1	9
1.19315	80.422	1	2	1	7
1.18973	80.700	2	2	2	0
1.16002	83.217	1	3	0	6
1.14721	84.359	3	2	2	3
1.13864	85.143	1	1	3	1
1.12566	86.363	2	3	1	2
1.12419	86.503	3	1	2	8
1.09903	88.997	9	0	2	10

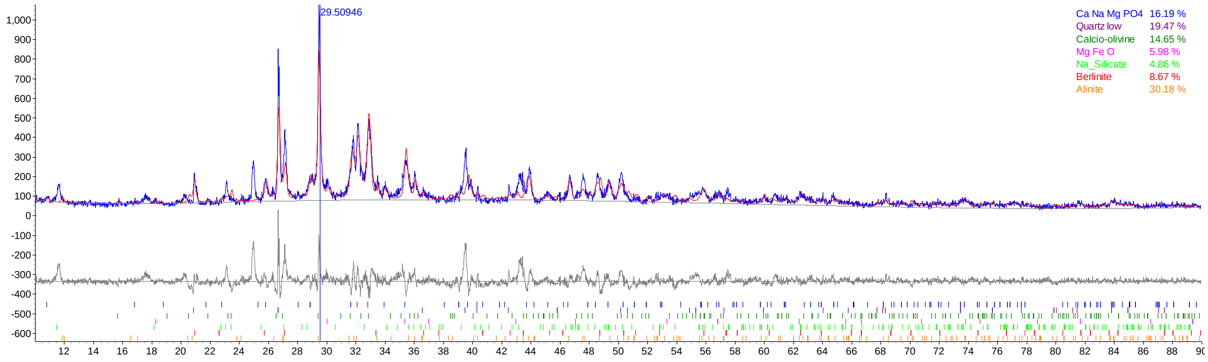

Fig. S34 Raw diffractogram of the SSA obtained from BNP

Table S24 Analysis from the SSA obtained from BNP

```
R-Values

Rexp:10.18      Rwp : 19.47     Rp  : 14.92   GOF : 1.91
Rexp`:10.23     Rwp`: 19.58     Rp` : 16.31   DW  : 0.59

Quantitative Analysis - Rietveld
   Phase 1   : "Quartz low"                        19.475 %
   Phase 2   : Calcio-olivine                      14.647 %
   Phase 3   : "Mg Fe O"                           5.981 %
   Phase 4   : Na_Silicate                         4.859 %
   Phase 5   : Berlinite                           8.669 %
   Phase 6   : Alinite                             30.178 %
   Phase 7   : "Ca Na Mg PO4"                      16.191 %

Degree of crystallinity (%)                        95.52
   Crystalline area                                2343.511
   Amorphous area                                  109.857

Background
   One on X                                        1938.949
   Chebychev polynomial, Coefficient  0            -5.203
                                      1            45.86669
                                      2            -40.04325
                                      3            22.01416

Instrument
   Primary radius (mm)                             250
   Secondary radius (mm)                           250
   Receiving slit width (mm)                       0.3
   FDS angle (°)                                   0.5563
      Beam spill, Sample length (mm)               25
         Intensity corrected
   Full Axial Convolution
      Filament length (mm)                         16
      Sample length (mm)                           25
      Receiving Slit length (mm)                   16
      Primary Sollers (°)                          2.5
      Secondary Sollers (°)                        2.5

Corrections
   Specimen displacement                           -0.1532128
   LP Factor                                       26.4
```

Table S25 Analysis from the SSA obtained from BNP - Structures 1-6

Miscellaneous

Convolution Steps	4

Structure 1

Phase name	Quartz low
R-Bragg	8.161
Spacegroup	P3221
Scale	3.24934e-004
Cell Mass	180.253
Cell Volume (Å^3)	113.13818
Wt% - Rietveld	19.475
Double-Voigt|Approach	
Cry size Lorentzian	65.4
Cry size Gaussian	999.2
k: 1 LVol-IB (nm)	41.638
k: 0.89 LVol-FWHM (nm)	58.049
Crystal Linear Absorption Coeff. (1/cm)	94.746
Crystal Density (g/cm^3)	2.646
Lattice parameters	
a (Å)	4.9156800
c (Å)	5.4064394

Site	Np	x	y	z	Atom	Occ	Beq
Si1	3	0.47230	0.00000	0.66667	Si+4	1	0.793
O1	6	0.41600	0.26580	0.78810	O-2	1	1.479

Structure 2

Phase name	Calcio-olivine
R-Bragg	7.323
Spacegroup	Pbnm
Scale	1.86040e-005
Cell Mass	688.956
Cell Volume (Å^3)	388.83063
Wt% - Rietveld	14.647
Double-Voigt|Approach	
Cry size Lorentzian	80.0
Cry size Gaussian	80.0
k: 1 LVol-IB (nm)	35.916
k: 0.89 LVol-FWHM (nm)	43.399
Crystal Linear Absorption Coeff. (1/cm)	275.219
Crystal Density (g/cm^3)	2.942
Lattice parameters	
a (Å)	5.1139381
b (Å)	11.3244180
c (Å)	6.7141202

Site	Np	x	y	z	Atom	Occ	Beq
Ca1	4	0.00000	0.00000	0.00000	Ca	1	1
Ca2	4	-0.00990	0.28090	0.25000	Ca	1	1
Si	4	0.42750	0.09660	0.25000	Si	1	1
O1	4	-0.25430	0.09370	0.25000	O	1	1
O2	4	0.29740	-0.03840	0.25000	O	1	1
O3	8	0.29850	0.16240	0.05750	O	1	1

Structure 3

Phase name	Mg Fe O
R-Bragg	8.022
Spacegroup	Fd-3mS

Scale	2.23580e-006
Cell Mass	1528.179
Cell Volume (Å^3)	595.67210
Wt% - Rietveld	5.981
Double-Voigt|Approach	
Cry size Lorentzian	537807173100000000.0
Cry size Gaussian	50.0
k: 1 LVol-IB (nm)	46.972
k: 0.89 LVol-FWHM (nm)	44.500
Crystal Linear Absorption Coeff. (1/cm)	650.512
Crystal Density (g/cm^3)	4.260
Lattice parameters	
a (Å)	8.4139983

Site	Np	x	y	z	Atom	Occ	Beq
Mg1	8	0.00000	0.00000	0.00000	Mg+2	0.29	1.07
Fe1	8	0.00000	0.00000	0.00000	Fe+3	0.71	1.07
Mg2	16	0.62500	0.62500	0.62500	Mg+2	0.555	1.6
Fe2	16	0.62500	0.62500	0.62500	Fe+3	0.445	1.6
Mg3	16	0.12500	0.12500	0.12500	Mg+2	0.075	0.9
O1	32	0.37910	0.37910	0.37910	O-2	1	1.29

Structure 4

Phase name	Na_Silicate
R-Bragg	15.214
Spacegroup	Pcnb
Scale	4.66115e-006
Cell Mass	728.590
Cell Volume (Å^3)	486.78989
Wt% - Rietveld	4.859
Double-Voigt|Approach	
Cry size Lorentzian	9401007174000000.0
Cry size Gaussian	70.0
k: 1 LVol-IB (nm)	65.761
k: 0.89 LVol-FWHM (nm)	62.300
Crystal Linear Absorption Coeff. (1/cm)	79.811
Crystal Density (g/cm^3)	2.485
Preferred Orientation Spherical Harmonics	
Order	2
y00	1
y20	0
y22p	0
Lattice parameters	
a (Å)	6.3107844
b (Å)	15.4467458
c (Å)	4.9936857

Site	Np	x	y	z	Atom	Occ	Beq
Na1	8	0.09888	0.56036	0.23704	Na+1	1	0
Si1	8	0.10516	0.34298	0.29624	Si+4	1	0
O1	4	0.00000	0.25000	0.23929	O-2	1	0
O2	8	0.17933	0.34786	0.61707	O-2	1	0
O3	8	-0.04692	0.42141	0.23945	O-2	1	0

Structure 5

Phase name	Berlinite
R-Bragg	14.498
Spacegroup	P3121
Scale	2.52512e-005

Cell Mass	539.725
Cell Volume (Å^3)	216.44433
Wt% - Rietveld	8.669
Double-Voigt|Approach	
Cry size Lorentzian	148.3
Cry size Gaussian	80.0
k: 1 LVol-IB (nm)	48.100
k: 0.89 LVol-FWHM (nm)	53.841
Crystal Linear Absorption Coeff. (1/cm)	185.747
Crystal Density (g/cm^3)	4.141
Preferred Orientation Spherical Harmonics	
Order	4
y00	1
y20	1.596729
y40	0
y43m	1.734723e-018
Lattice parameters	
a (Å)	4.9024520
c (Å)	10.3989357

Site	Np	x	y	z	Atom	Occ	Beq
Al	6	0.45850	0.00000	0.00000	Al	1	1
P	6	0.45840	0.00000	0.50000	P	1	1
O1	6	0.40840	0.30600	0.05810	O	1	1
O2	6	0.41010	0.27420	0.54350	O	1	1

Structure 6

Phase name	Alinite
R-Bragg	9.119
Spacegroup	I4/mmm
Scale	5.14818e-006
Cell Mass	1710.698
Cell Volume (Å^3)	1165.94048
Wt% - Rietveld	30.178
Double-Voigt|Approach	
Cry size Lorentzian	65.3
Cry size Gaussian	11636271810000000.0
k: 1 LVol-IB (nm)	41.624
k: 0.89 LVol-FWHM (nm)	58.184
Crystal Linear Absorption Coeff. (1/cm)	230.842
Crystal Density (g/cm^3)	2.436
Preferred Orientation Spherical Harmonics	
Order	4
y00	1
y20	0.9960318
y40	-1.152651
y44p	-0.2094222
Lattice parameters	
a (Å)	10.4269987
c (Å)	10.7240232

Site	Np	x	y	z	Atom	Occ	Beq
Ca1	16	0.00000	0.26150	0.29100	Ca+2	1	1.49
Ca2	2	0.00000	0.00000	0.00000	Ca+2	1	0.71
Ca3	4	0.00000	0.50000	0.00000	Ca+2	0.36	0.71
Mg1	4	0.00000	0.50000	0.00000	Mg+2	0.64	0.71
Si1	8	0.21070	0.21070	0.00000	Si+4	0.75	0.57
Al1	8	0.21070	0.21070	0.00000	Al+3	0.25	0.57
O1	16	0.17960	0.36470	0.00000	O-2	1	1.47
O2	16	0.14430	0.14430	0.15370	O-2	1	0.92

O3	4	0.00000	0.50000	0.25000	O-2	1	1.47
Cl1	2	0.00000	0.00000	0.50000	Cl-1	1	2.33

Table S26 Analysis from the SSA obtained from BNP - Peaks

Peaks Phase 1

Phase name: Peaks Phase:0

Type	Position	I		
FP	29.50946	5	Cry size Lor	2.0
			Cry size Gauss	2.0
			k: 1, LVol-IB(nm)	0.898
			k: 0.89, LVol-FWHM(nm)	1.085

Peaks Phase 2

Phase name: Peaks Phase:1

Type	Position	I

hkl Phase - 7 Lebail method

Phase name	Ca Na Mg PO4
R-Bragg	1.551
Spacegroup	176
Scale	8.58233e-012
Cell Mass	1199999999.000
Cell Volume (Å^3)	534.92948
Wt% - Rietveld	16.191
Double-Voigt|Approach	
Cry size Lorentzian	70.0
Cry size Gaussian	55.0
k: 1 LVol-IB (nm)	28.063
k: 0.89 LVol-FWHM (nm)	32.878
Lattice parameters	
a (Å)	9.4561338
c (Å)	6.9077830

h	k	l	m	d	Th2	I
0	1	0	6	8.18925	10.79468	4.28e+009
0	1	1	12	5.28016	16.77709	4.87e+009
1	1	0	6	4.72807	18.75288	1.01e-039
0	2	0	6	4.09463	21.68666	3.58e-040
1	1	1	12	3.90166	22.77320	1.6e+010
0	2	1	12	3.52232	25.26426	1.12e+010
0	0	2	2	3.45389	25.77333	1.08e+011
0	1	2	12	3.18242	28.01484	1.97e+010
2	1	0	6	3.09525	28.82070	8.32e+010
1	2	0	6	3.09525	28.82070	8.32e+010
1	2	1	12	2.82465	31.65070	1.99e+011
2	1	1	12	2.82465	31.65070	1.99e+011
1	1	2	12	2.78899	32.06620	5.61e+011
0	3	0	6	2.72975	32.78146	7.4e+011
0	2	2	12	2.64008	33.92797	1.21e+011
0	3	1	12	2.53872	35.32631	3.41e+011
2	2	0	6	2.36403	38.03302	1.62e-040
2	1	2	12	2.30507	39.04478	7.35e+010
1	2	2	12	2.30507	39.04478	7.35e+010
1	3	0	6	2.27129	39.64966	1.96e+011
3	1	0	6	2.27129	39.64966	1.96e+011
2	2	1	12	2.23668	40.28956	2.05e-010
0	1	3	12	2.21664	40.66983	7.31e-041
3	1	1	12	2.15765	41.83310	1.47e-040

1	3	1	12	2.15765	41.83310	1.47e-040
0	3	2	12	2.14163	42.16087	1.3e-040
1	1	3	12	2.07015	43.69007	4.09e+011
0	4	0	6	2.04731	44.20297	1.36e-040
0	2	3	12	2.00702	45.13882	1.35e+011
0	4	1	12	1.96292	46.21099	2.88e-040
2	2	2	12	1.95083	46.51402	3.49e+011
1	3	2	12	1.89773	47.89574	4.5e+010
3	1	2	12	1.89773	47.89574	4.5e+010
3	2	0	6	1.87874	48.41056	2.15e+011
2	3	0	6	1.87874	48.41056	2.15e+011
2	1	3	12	1.84746	49.28435	1.97e+011
1	2	3	12	1.84746	49.28435	1.97e+011
3	2	1	12	1.81289	50.28876	6.43e+010
2	3	1	12	1.81289	50.28876	6.43e+010
1	4	0	6	1.78704	51.06799	2.96e-040
4	1	0	6	1.78704	51.06799	2.96e-040
0	4	2	12	1.76116	51.87379	2.83e-040
0	3	3	12	1.76005	51.90882	2.07e-040
4	1	1	12	1.73009	52.87701	7.37e+010
1	4	1	12	1.73009	52.87701	1.96e+010
0	0	4	2	1.72695	52.98062	2.79e-024
0	1	4	12	1.68978	54.24011	6.31e-040
3	2	2	12	1.65038	55.64563	1.05e+011
2	3	2	12	1.65038	55.64563	3.71e+011
2	2	3	12	1.64947	55.67900	0.00374
0	5	0	6	1.63785	56.10889	2.03e-035
1	1	4	12	1.62213	56.70163	2.2e+011
3	1	3	12	1.61700	56.89785	3.64e+010
1	3	3	12	1.61700	56.89785	3.64e+010
0	5	1	12	1.59367	57.80878	9.32e+010
0	2	4	12	1.59121	57.90641	9.96e+010
4	1	2	12	1.58718	58.06755	0.313
1	4	2	12	1.58718	58.06755	0.313
3	3	0	6	1.57602	58.51834	1.89e-040
4	2	0	6	1.54762	59.69982	4.86e+004
2	4	0	6	1.54762	59.69982	4.86e+004
3	3	1	12	1.53654	60.17477	1.67e-040
0	4	3	12	1.53000	60.45893	2.62e+011
2	4	1	12	1.51019	61.33676	1.13e+011
4	2	1	12	1.51019	61.33676	1.13e+011
2	1	4	12	1.50810	61.43093	4.28e+006
1	2	4	12	1.50810	61.43093	4.28e+006
0	5	2	12	1.47989	62.73301	3.59e+011
5	1	0	6	1.47083	63.16362	4.76e+004
1	5	0	6	1.47083	63.16362	5.83e+004
0	3	4	12	1.45941	63.71560	1.68e+011
3	2	3	12	1.45568	63.89859	5.3e+010
2	3	3	12	1.45568	63.89859	5.3e+010
1	5	1	12	1.43858	64.74963	1.69e+011
5	1	1	12	1.43858	64.74963	1.69e+011
3	3	2	12	1.43381	64.99187	1.22e+011
2	4	2	12	1.41232	66.10564	1.78e+010
4	2	2	12	1.41232	66.10564	1.78e+010
4	1	3	12	1.41175	66.13578	1.57e+010
1	4	3	12	1.41175	66.13578	1.57e+010
2	2	4	12	1.39449	67.06161	2.7e-014
3	1	4	12	1.37471	68.15816	1.81e-022
1	3	4	12	1.37471	68.15816	1.81e-022
0	6	0	6	1.36488	68.71745	4.61e+008
0	1	5	12	1.36231	68.86524	1.39e+011

1	5	2	12	1.35324	69.39242	1.4e+011
5	1	2	12	1.35324	69.39242	1.4e+011
4	3	0	6	1.34630	69.80159	9.07e-019
3	4	0	6	1.34630	69.80159	5.3e-018
0	6	1	12	1.33899	70.23898	0.000304
0	5	3	12	1.33465	70.50108	1.27e+011
1	1	5	12	1.32610	71.02400	1.22e+011
3	4	1	12	1.32144	71.31273	2.48e-007
4	3	1	12	1.32144	71.31273	2.81e-007
0	4	4	12	1.32004	71.39999	1.9e+011
5	2	0	6	1.31133	71.94791	1.89e+011
2	5	0	6	1.31133	71.94791	1.57e+011
0	2	5	12	1.30905	72.09277	0.087
3	3	3	12	1.30055	72.63856	2.96e+011
5	2	1	12	1.28832	73.44044	2.43e+011
2	5	1	12	1.28832	73.44044	2.43e+011
4	2	3	12	1.28446	73.69782	1.49e+011
2	4	3	12	1.28446	73.69782	1.49e+011
3	2	4	12	1.27142	74.58113	3.82e+009
2	3	4	12	1.27142	74.58113	3.82e+009
0	6	2	12	1.26936	74.72282	2.19e+011
1	2	5	12	1.26159	75.26252	1.85e+010
2	1	5	12	1.26159	75.26252	7.45e+010
3	4	2	12	1.25438	75.77127	4.8e+010
4	3	2	12	1.25438	75.77127	4.8e+010
1	6	0	6	1.24885	76.16656	3.82e+010
6	1	0	6	1.24885	76.16656	1.24e+011
4	1	4	12	1.24184	76.67450	1.84
1	4	4	12	1.24184	76.67450	1.84
5	1	3	12	1.23953	76.84318	1.41e+011
1	5	3	12	1.23953	76.84318	1.85e+011
0	3	5	12	1.23267	77.34970	1.38e+011
1	6	1	12	1.22893	77.62962	3.76e+003
6	1	1	12	1.22893	77.62962	3.76e+003
5	2	2	12	1.22594	77.85404	7.39e+010
2	5	2	12	1.22594	77.85404	7.39e+010
2	2	5	12	1.19280	80.44968	6.74e+010
0	5	4	12	1.18838	80.81043	3.47e-040
4	4	0	6	1.18202	81.33694	4.94e-030
1	3	5	12	1.18035	81.47630	6.27e-009
3	1	5	12	1.18035	81.47630	3.01e+011
6	1	2	12	1.17444	81.97406	1.71e+006
1	6	2	12	1.17444	81.97406	2.62e+006
0	6	3	12	1.17411	82.00189	2.87e+006
0	7	0	6	1.16989	82.36118	2.29e+010
3	5	0	6	1.16989	82.36118	3.63e+010
5	3	0	6	1.16989	82.36118	5.06e+010
4	4	1	12	1.16508	82.77572	2.5e-008
3	3	4	12	1.16412	82.85905	1.12e-007
4	3	3	12	1.16222	83.02449	1.51e+011
3	4	3	12	1.16222	83.02449	1.51e+011
0	7	1	12	1.15347	83.79660	2.69e+011
5	3	1	12	1.15347	83.79660	2.32e+011
3	5	1	12	1.15347	83.79660	2.64e+011
2	4	4	12	1.15254	83.87975	1.7e+007
4	2	4	12	1.15254	83.87975	294
0	0	6	2	1.15130	83.99061	1.99e+008
0	4	5	12	1.14520	84.54105	3.83e+011
0	1	6	12	1.14009	85.00918	2.23e+011
5	2	3	12	1.13950	85.06334	0.0145
2	5	3	12	1.13950	85.06334	0.000761

6	2	0	6	1.13564	85.42052	2.33e-030
2	6	0	6	1.13564	85.42052	4.78e-030
2	6	1	12	1.12060	86.84873	1.86e+011
6	2	1	12	1.12060	86.84873	1.77e+011
5	1	4	12	1.11975	86.93153	0.0039
1	5	4	12	1.11975	86.93153	1.08e+004
1	1	6	12	1.11861	87.04191	6.08e-012
4	4	2	12	1.11834	87.06835	0.00203
3	2	5	12	1.11302	87.59021	5.7e+010
2	3	5	12	1.11302	87.59021	1.17e+011
0	2	6	12	1.10832	88.05672	3.74e+009
0	7	2	12	1.10806	88.08314	5.49e+010
5	3	2	12	1.10806	88.08314	9.53e+010
3	5	2	12	1.10806	88.08314	5.49e+010
6	1	3	12	1.09778	89.12489	3.06e-040
1	6	3	12	1.09778	89.12489	3.06e-040
4	1	5	12	1.09301	89.61865	2.56e-040
1	4	5	12	1.09301	89.61865	2.56e-040

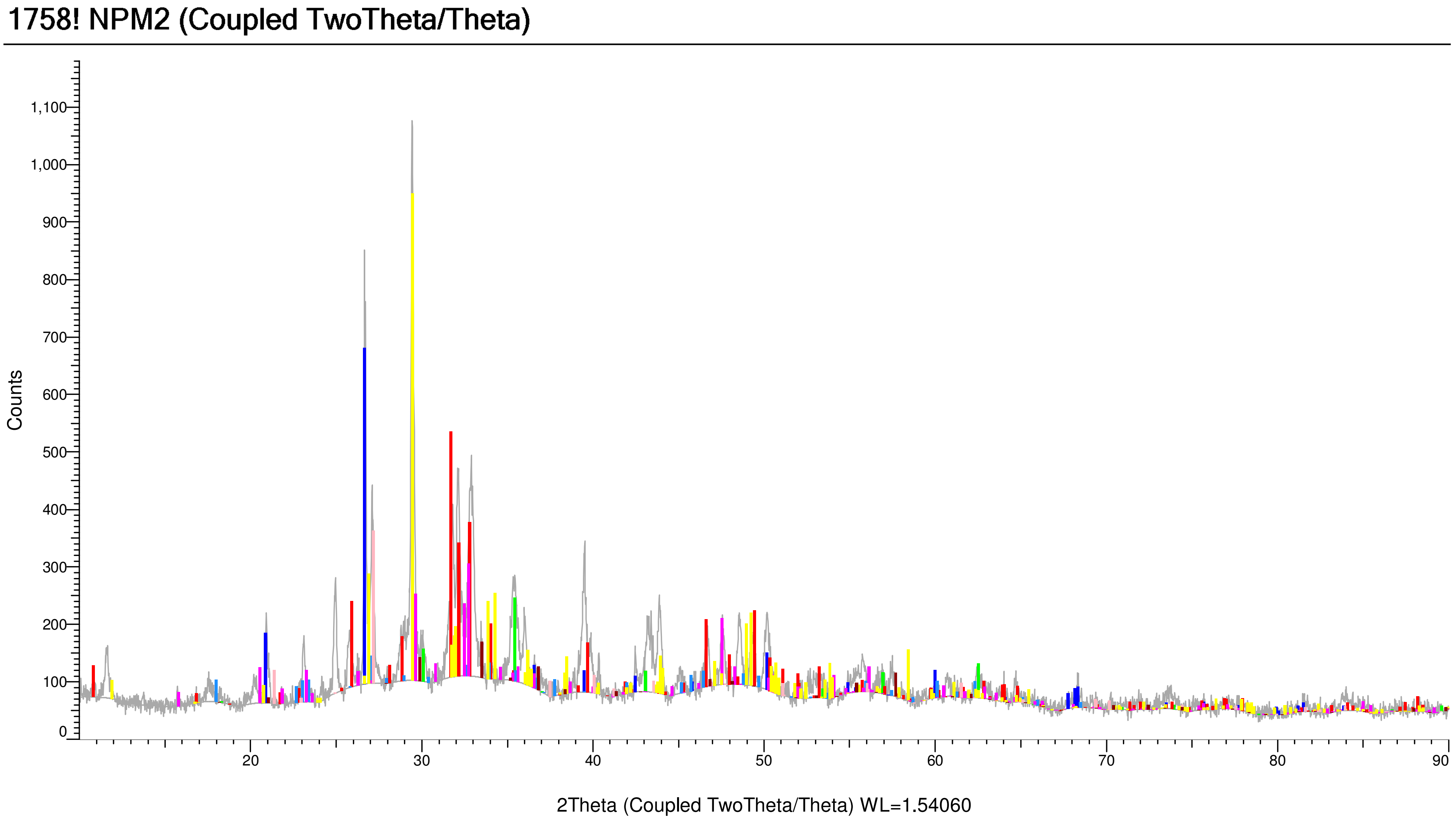

Fig. S35 High definition diffractogram of the SSA obtained from BNP

Table S27 Patterns from the SSA obtained from BNP. Pattern: PDF 78-2315, Radiation: 1.54060

**Formula**	SiO2
**Name**	Silicon Oxide
**Name (mineral)**	Quartz
**Name (common)**	
**Status**	Primary
**Ambient**	Yes

**Lattice:**	Hexagonal	**Mol. weight =**	60.08
**S.G.:**	P3221 (154)	**Volume [CD] =**	112.93
		**Dx =**	
		**Dm =**	
		**I/Icor =**	3.100

a =	4.91239	Z =	3
c =	5.40385		
a/b =	1.00000		
c/b =	1.10004		

ICSD Collection Code: 063532
Additional Pattern: See PDF 87-2096, PDF 85-335, PDF 85-457, PDF 85-504, PDF 85-695, PDF 85-794, PDF 85-795, PDF 85-796, PDF 85-797, PDF 85-798, PDF 85-930, PDF 85-1053, PDF 85-1054, PDF 85-1780, PDF 86-1560 and PDF 86-1561
Article Title: Crystal structures of quartz and magnesium germanate by profile analysis of synchrotron-radiation high-resolution powder data

Structure
Publication: J. Appl. Crystallogr.
Detail: volume 21, page 182 (1988)
Authors: Will, G., Bellotto, M., Parrish, W., Hart, M.
Primary Reference
Publication: Calculated from ICSD using POWD-12++

**Radiation:**	CuKa1	**Filter:**	Not specified
**Wavelength:**	1.54060	**d-spacing:**	
**SS/FOM:**	F(29)= 999.9 (0.0001, 29)		

d	2θ	I fix	h	k	l
4.25425	20.864	209	1	0	0
3.34268	26.646	1000	0	1	1
2.45620	36.554	69	1	1	0
2.28080	39.478	66	1	0	2
2.23605	40.302	33	1	1	1
2.12713	42.462	50	2	0	0
1.97930	45.807	29	2	0	1
1.81747	50.153	111	1	1	2
1.80128	50.636	5	0	0	3
1.67134	54.889	35	0	2	2
1.65873	55.342	16	0	1	3
1.60796	57.247	3	2	1	0
1.54118	59.975	84	1	2	1
1.45254	64.053	15	1	1	3
1.41808	65.803	5	3	0	0
1.38178	67.762	47	1	2	2
1.37463	68.163	59	2	0	3
1.37164	68.332	63	0	3	1
1.28760	73.489	19	1	0	4
1.25565	75.681	26	3	0	2
1.22810	77.692	15	2	2	0
1.19955	79.906	25	2	1	3
1.19756	80.065	15	2	2	1
1.18372	81.196	20	1	1	4
1.17992	81.512	27	3	1	0
1.15276	83.860	16	1	3	1
1.14040	84.980	3	2	0	4
1.11803	87.099	2	2	2	2
1.11423	87.471	3	3	0	3

Table S28 Patterns from the SSA obtained from BNP. Pattern: PDF 89-6442, Radiation: 1.54060

**Formula**	(Ca3.892Na0.087Mg0.021)(Ca5.589Na0.125Mg0.028)(PO4)5.5
**Name**	Calcium Sodium Magnesium Phosphate
**Name (mineral)**	
**Name (common)**	
**Status**	Primary
**Ambient**	Yes

Lattice:	Hexagonal	Mol. weight =	908.41
S.G.:	P63/m (176)	Volume [CD] =	532.25
		Dx =	
		Dm =	
		I/Icor =	1.070

a =	9.45120	
c =	6.88030	
a/b =	1.00000	Z = 1
c/b =	0.72798	

Sample Source Or Locality: Specimen from human teeth
Test From ICSD: At least one SOF implausible
Test From ICSD: At least one TF missing
Test From ICSD: Charge sum slightly deviates from zero
Test From ICSD: Calcul. formula slightly deviates from input
Remark From ICSD/CSD: REM RVP
Remark From ICSD/CSD: ATOM O 5 -2. 1.72 Atoms not located in unit cell
ICSD Collection Code: 087673
Article Title: Rietveld refinement of the crystallographic structure of human dental enamel apatites

Structure
Publication: Am. Mineral.
Detail: volume 84, page 1406 (1999)
Authors: Wilson, R.M., Elliott, J.C., Dowker, S.E.P.
Primary Reference
Publication: Calculated from ICSD using POWD-12++

**Radiation:**	CuKa1	**Filter:**	Not specified
**Wavelength:**	1.54060	**d-spacing:**	
**SS/FOM:**	F(30)= 999.9 (0.0001, 31)		

d	2θ	I fix	h	k	l	d	2θ	I fix	h	k	l
8.18498	10.800	129	1	0	0	1.78611	51.097	112	1	4	0
5.26672	16.820	41	1	0	1	1.75865	51.954	102	4	0	2
4.72560	18.763	5	1	1	0	1.75557	52.051	62	3	0	3
4.09249	21.698	49	2	0	0	1.72881	52.919	10	4	1	1
3.89531	22.811	60	1	1	1	1.72008	53.209	129	0	0	4
3.51730	25.301	15	2	0	1	1.68331	54.466	13	1	0	4
3.44015	25.878	352	0	0	2	1.64821	55.726	51	3	2	2
3.17142	28.114	75	1	0	2	1.63700	56.141	3	5	0	0
3.09363	28.836	183	2	1	0	1.61339	57.037	35	1	3	3
2.82153	31.687	1000	2	1	1	1.59254	57.854	16	5	0	1
2.78124	32.158	546	1	1	2	1.58519	58.148	16	4	1	2
2.72833	32.799	627	3	0	0	1.57520	58.552	13	3	3	0
2.63336	34.017	227	2	0	2	1.54682	59.734	45	2	4	0
2.53620	35.363	45	3	0	1	1.53547	60.221	30	3	3	1
2.36280	38.054	9	2	2	0	1.52685	60.597	3	4	0	3
2.30031	39.129	29	2	1	2	1.50915	61.384	25	2	4	1
2.27010	39.671	203	1	3	0	1.50333	61.647	45	1	2	4
2.23470	40.327	13	2	2	1	1.47817	62.814	71	5	0	2
2.20838	40.829	2	1	0	3	1.47007	63.200	14	5	1	0
2.15579	41.871	51	1	3	1	1.45505	63.930	70	3	0	4
2.13766	42.243	15	3	0	2	1.45290	64.035	73	3	2	3
2.06328	43.843	37	1	1	3	1.43762	64.799	67	5	1	1
2.04624	44.227	14	4	0	0	1.41077	66.188	12	4	2	2
2.00069	45.290	41	2	0	3	1.40918	66.272	15	4	1	3
1.94765	46.595	275	2	2	2	1.39062	67.273	2	2	2	4
1.89475	47.976	123	1	3	2	1.37097	68.370	5	3	1	4
1.87776	48.438	34	2	3	0	1.36416	68.759	4	6	0	0
1.84238	49.429	307	2	1	3	1.35702	69.172	2	1	0	5
1.81151	50.330	136	3	2	1	1.35181	69.477	18	1	5	2

d	2θ	I fix	h	k	l
1.34560	69.844	3	4	3	0
1.33812	70.292	2	6	0	1
1.33240	70.638	3	5	0	3
1.32058	71.367	36	4	3	1
1.31668	71.611	21	4	0	4
1.31065	71.991	33	5	2	0
1.30430	72.397	4	2	0	5
1.29844	72.776	3	3	3	3
1.28749	73.496	19	5	2	1
1.28240	73.836	30	2	4	3
1.26810	74.810	22	6	0	2
1.26810	74.810	22	2	3	4
1.25729	75.565	39	2	1	5
1.25315	75.859	24	3	4	2
1.24820	76.214	16	6	1	0
1.23896	76.885	45	1	4	4
1.23764	76.982	41	5	1	3
1.22815	77.688	8	1	6	1
1.22477	77.943	51	2	5	2
1.18910	80.752	3	2	2	5
1.18581	81.023	5	5	0	4
1.18140	81.389	17	4	4	0
1.17675	81.779	5	1	3	5
1.17335	82.066	6	6	0	3
1.17335	82.066	6	1	6	2
1.16928	82.414	8	7	0	0
1.16436	82.839	8	4	4	1
1.16059	83.167	31	4	3	3
1.15016	84.093	33	2	4	4
1.14672	84.403	22	0	0	6
1.13794	85.208	13	5	2	3
1.13563	85.422	9	2	6	0
1.13563	85.422	9	1	0	6
1.11753	87.148	20	4	4	2
1.11753	87.148	20	5	1	4
1.11438	87.456	39	1	1	6
1.10993	87.896	20	2	3	5
1.10708	88.181	63	5	3	2
1.10419	88.472	30	2	0	6
1.09634	89.274	3	1	6	3
1.09007	89.926	2	4	1	5

Table S29 Patterns from the SSA obtained from BNP. Pattern: COD 9014595, Radiation: 1.54060

Formula	Ca2O4Si
Name	
Name (mineral)	Calcio-olivine
Name (common)	
Status	Status Unknown
Ambient	Yes

Lattice:	Orthorhombic	Mol. weight =	
S.G.:	P b n m (62)	Volume [CD] =	386.54
		Dx =	
		Dm =	
		I/Icor =	1.440

a =	5.08100
b =	11.22400
c =	6.77800
a/b =	0.45269
c/b =	0.60388

- Synthetic:

Primary Reference
Udagawa S., Urabe K., Natsume M., Yano T., "Refinement of the crystal structure of gamma-Ca2SiO4", Cement and Concrete Research 10 (1980) 139-144.

Wavelength:	1.54060	Filter:	Not specified
SS/FOM:		d-spacing:	

d	2θ	I fix	h	k	l	d	2θ	I fix	h	k	l
5.61200	15.779	128	0	2	0	1.96510	46.157	51	1	5	1
4.32260	20.530	318	0	2	1	1.93750	46.853	41	1	2	3
4.06550	21.844	131	1	0	1	1.91120	47.537	588	2	2	2
3.82250	23.251	284	1	1	1	1.88330	48.286	163	2	4	0
3.76660	23.601	84	1	2	0	1.87070	48.632	4	0	6	0
3.38900	26.276	124	0	0	2	1.81450	50.241	112	2	4	1
3.29240	27.061	30	1	2	1	1.80750	50.449	160	1	3	3
3.01270	29.628	772	1	3	0	1.80320	50.578	134	0	6	1
2.90110	30.796	163	0	2	2	1.78610	51.097	6	2	3	2
2.80600	31.867	41	0	4	0	1.75980	51.917	101	0	4	3
2.75300	32.497	642	1	3	1	1.75610	52.035	104	1	5	2
2.73440	32.724	999	1	1	2	1.75550	52.054	7	1	6	0
2.59260	34.569	111	0	4	1	1.69940	53.908	8	1	6	1
2.54050	35.301	74	2	0	0	1.69450	54.077	202	0	0	4
2.51930	35.608	141	1	2	2	1.68220	54.505	7	2	5	0
2.47780	36.225	70	2	1	0	1.67470	54.769	51	3	1	0
2.45630	36.553	129	1	4	0	1.66950	54.954	12	2	1	3
2.32720	38.659	98	2	1	1	1.66290	55.191	15	1	4	3
2.25160	40.011	57	1	3	2	1.64620	55.800	37	2	4	2
2.19020	41.183	25	2	2	1	1.64310	55.914	8	3	0	1
2.16130	41.759	22	0	4	2	1.63770	56.115	229	0	6	2
2.10170	43.001	7	2	3	0	1.63270	56.302	16	2	5	1
2.09590	43.126	2	0	2	3	1.62580	56.562	57	3	1	1
2.06440	43.818	12	1	0	3	1.62220	56.699	15	0	2	4
2.05330	44.067	30	1	5	0	1.62140	56.730	16	3	2	0
2.03280	44.536	4	2	0	2	1.61670	56.909	6	2	2	3
2.03040	44.591	86	1	1	3	1.59120	57.907	1	1	1	4
2.00020	45.301	1	2	1	2	1.57690	58.483	10	3	2	1

d	2θ	I fix	h	k	l	d	2θ	I fix	h	k	l	d	2θ	I fix	h	k	l	d	2θ	I fix	h	k	l
1.55880	59.229	2	1	6	2	1.34540	69.856	7	3	1	3	1.19110	80.588	15	3	1	4	1.10020	88.877	25	2	4	5
1.54290	59.901	35	3	3	0	1.33310	70.596	7	3	4	2	1.18930	80.736	12	2	1	5	1.09770	89.134	21	0	6	5
1.53890	60.073	8	2	3	3	1.31920	71.453	4	2	3	4	1.18690	80.933	1	1	4	5	1.09750	89.154	4	3	6	3
1.52910	60.498	104	1	7	0	1.31770	71.547	13	0	2	5	1.18280	81.272	21	4	1	2	1.09750	89.154	47	1	1	6
1.51950	60.921	8	1	5	3	1.31730	71.572	1	3	2	3	1.17150	82.224	20	3	2	4	1.09600	89.309	1	1	10	0
1.50680	61.490	2	2	5	2	1.30980	72.045	8	1	0	5	1.16970	82.378	4	2	2	5	1.09110	89.818	16	4	5	1
1.50440	61.598	51	3	3	1	1.30690	72.230	15	1	5	4	1.16440	82.835	41	3	7	0	1.08630	90.324	4	4	2	3
1.50140	61.735	64	3	1	2	1.30100	72.610	6	1	1	5	1.16360	82.905	43	4	2	2	1.08190	90.794	1	1	10	1
1.49160	62.186	8	1	7	1	1.29630	72.915	8	0	8	2	1.16260	82.992	2	2	7	3	1.08070	90.923	7	0	8	4
1.47690	62.875	108	1	3	4	1.27420	74.391	29	3	3	3	1.16020	83.202	8	3	5	3	1.08040	90.955	13	3	8	0
1.47050	63.180	20	2	6	1	1.27020	74.665	31	4	0	0	1.15720	83.466	19	4	4	0	1.07900	91.107	25	2	8	3
1.46270	63.556	74	3	2	2	1.26630	74.934	8	1	7	3	1.15470	83.687	14	2	8	2	1.07290	91.773	4	1	6	5
1.45050	64.154	21	0	4	4	1.25970	75.395	43	2	4	4	1.14080	84.944	41	3	3	4	1.06750	92.372	2	1	9	3
1.45000	64.179	21	3	4	0	1.25890	75.452	18	2	7	2	1.14070	84.953	6	4	4	1	1.06550	92.597	1	0	10	2
1.44660	64.348	14	2	4	3	1.25610	75.649	13	1	8	2	1.14050	84.971	93	1	9	2	1.06300	92.879	6	2	9	2
1.44090	64.633	47	0	6	3	1.25580	75.671	60	3	5	2	1.13920	85.091	1	2	3	5	1.06170	93.027	7	4	3	3
1.41790	65.813	29	3	4	1	1.25330	75.848	15	2	6	3	1.13520	85.462	74	1	7	4	1.05870	93.370	6	2	7	4
1.40970	66.245	51	2	0	4	1.23620	77.089	41	1	3	5	1.13350	85.621	8	4	3	2	1.05700	93.565	1	1	8	4
1.40300	66.602	6	0	8	0	1.23450	77.214	20	3	6	1	1.13130	85.828	8	1	5	5	1.05680	93.588	2	3	5	4
1.39870	66.834	7	2	1	4	1.22820	77.684	16	2	8	0	1.12970	85.979	11	0	0	6	1.05550	93.739	4	2	5	5
1.39480	67.045	4	1	4	4	1.22060	78.260	4	0	4	5	1.12580	86.349	2	2	6	4	1.05370	93.948	18	3	1	5
1.39380	67.100	6	1	7	2	1.22030	78.283	28	3	4	3	1.12240	86.675	11	0	10	0	1.05090	94.275	4	4	6	0
1.38620	67.517	7	1	6	3	1.21920	78.367	2	1	6	4	1.11950	86.956	2	2	9	0	1.04790	94.630	8	0	4	6
1.37650	68.057	39	2	6	2	1.21870	78.406	1	4	2	1	1.10750	88.139	14	0	2	6	1.04000	95.578	4	3	2	5
1.35590	69.237	4	2	7	0	1.20850	79.197	15	2	8	1	1.10730	88.159	16	0	10	1	1.03850	95.760	12	4	6	1
1.35520	69.278	15	3	0	3	1.20280	79.647	4	4	3	0	1.10550	88.340	7	4	5	0	1.03500	96.190	4	3	7	3
1.35200	69.465	6	3	5	0	1.19230	80.491	4	1	9	1	1.10190	88.704	4	4	1	3	1.03000	96.811	8	4	4	3
1.34930	69.624	15	2	5	3	1.19190	80.523	7	0	8	3	1.10170	88.724	8	3	4	4	1.02940	96.887	8	3	8	2

d	2θ	I fix	h	k	l
1.02790	97.075	4	2	1	6
1.02670	97.227	24	2	10	0
1.02630	97.278	2	1	4	6
1.01840	98.293	13	3	3	5
1.01640	98.554	26	4	0	4
1.01520	98.711	41	2	2	6
1.01510	98.724	6	2	10	1
1.01440	98.817	2	1	7	5
1.01220	99.108	6	4	1	4
1.01210	99.121	2	5	1	0
1.00760	99.723	7	2	6	5

Table S30 Patterns from the SSA obtained from BNP. Pattern: PDF 19-1237, Radiation: 1.54060

**Formula**	Na2Si2O5
**Name**	Sodium Silicate
**Name (mineral)**	
**Name (common)**	
**Status**	Primary
**Ambient**	Yes

**Lattice:**	Orthorhombic
**Mol. weight =**	182.15
**Volume [CD] =**	480.41
**Dx =**	
**Dm =**	
**I/Icor =**	-1.000
a =	6.44000
b =	15.04000
c =	4.96000
a/b =	0.42819
c/b =	0.32979
Z =	4

General Comments: The position of the (440) reflection, measured at elevated temperatures, was as follows: 185 C, 1.493, 360 C, 1.495; 435 C, 1.496; 552 C, 1.502; 616 C, 1.500; 637 C, 1.503
For the α#1 phase the position of the corresponding (420) reflections was as follows: 765 C and 807 C, 1.509; 830 C, 1.510
Sample Preparation: Synthetic, by crystallization from glass at 800-850 C, 12-24 hours
Temperature Of Data Collection: Pattern at 25 C
General Comments: Transforms to α II form at 678 C and to α I form at 707 C
General Comments: Unit cell parameters calculated from powder indexing
General Comments: This is form α III

Primary Reference
Publication: Phys. Chem. Glasses
Detail: volume 7, page 127 (1966)
Authors: Williamson, Glasser.

**Radiation:**	CuKa1
**Wavelength:**	1.54060
**SS/FOM:**	F(30)= 1.9(0.1450, 108)
**Filter:**	Not specified
**d-spacing:**	

d	2θ	I fix	h	k	l
4.93000	17.978	81	0	0	1
3.92000	22.665	60	1	0	1
3.86000	23.022	81	1	1	1
3.80000	23.391	81			
3.45000	25.803	6	1	2	1
3.29000	27.081	101	1	4	0
3.19000	27.947	21	2	0	0
3.08000	28.967	21	1	3	1
2.94000	30.378	21	2	2	0
2.74000	32.655	41	1	5	0
2.64000	33.929	60	2	1	1
2.53000	35.452	41	2	2	1
2.45000	36.650	41	0	1	2
2.42000	37.121	6	2	4	0
2.38000	37.768	60	2	3	1
2.28000	39.492	6	1	1	2
2.18600	41.266	21	2	4	1
2.15100	41.969	41	0	7	0
2.05800	43.962	21	3	2	0
2.00600	45.163	41	2	5	1
1.98200	45.741	60	2	6	0
1.95300	46.459	60	3	1	1
1.86600	48.763	41	3	4	0
1.83400	49.671	41	1	5	2
1.77000	51.596	6	0	6	2
1.73500	52.716	6	2	4	2
1.70700	53.649	21	1	6	2
1.64300	55.918	41	0	1	3
1.60300	57.441	21	1	0	3
1.57200	58.683	21	0	3	3
1.53800	60.112	60	1	9	1
1.51200	61.255	21	0	4	3
1.48900	62.306	60	3	4	2
1.43200	65.084	21	3	5	2
1.41500	65.965	21	3	8	0
1.36900	68.482	21	2	4	3

Table S31 Patterns from the SSA obtained from BNP. Pattern: PDF 80-0073, Radiation: 1.54060

Formula	Mg1.55Fe1.6O4
Name	Magnesium Iron Oxide
Name (mineral)	
Name (common)	
Status	Primary
Ambient	Yes

Lattice:	Cubic	Mol. weight =	191.03
S.G.:	Fd-3m (227)	Volume [CD] =	592.24
		Dx =	
		Dm =	
		I/Icor =	3.170

a =	8.39780	Z =	8
a/b =	1.00000		
c/b =	1.00000		

ICSD Collection Code: 067847
Remark From ICSD/CSD: REM RVP
Temperature Factor: ITF
Article Title: Synthesis and characterization of nickel and magnesium ferrites obtained from alpha-NaFeO2

Structure
Publication: Solid State Ionics
Detail: volume 63, page 429 (1993)
Authors: Blesa, M.C., Amador, U., Moran, E., Menendez, N., Tornero, J.D., Rodriguez-Carvajal, J.
Primary Reference
Publication: Calculated from ICSD using POWD-12++

Radiation:	CuKa1	Filter:	Not specified
Wavelength:	1.54060	d-spacing:	
SS/FOM:	F(18)= 999.9 (0.0002, 18)		

d	2θ	I fix	h	k	l
4.84847	18.283	23	1	1	1
2.96907	30.074	393	2	2	0
2.53203	35.423	999	3	1	1
2.42424	37.054	20	2	2	2
2.09945	43.050	248	4	0	0
1.92659	47.135	1	3	3	1
1.71419	53.406	108	4	2	2
1.61616	56.930	258	5	1	1
1.48454	62.514	411	4	4	0
1.41949	65.730	1	5	3	1
1.39963	66.783	1	4	4	2
1.32781	70.919	33	6	2	0
1.28065	73.954	58	5	3	3
1.26602	74.954	11	6	2	2
1.21212	78.914	21	4	4	4
1.17593	81.848	1	5	5	1
1.12220	86.695	30	6	4	2
1.09330	89.589	74	7	3	1

Table S32 Patterns from the SSA obtained from BNP. Pattern: PDF 78-2201, Radiation: 1.54060

**Formula**	(Ca19.44Mg2.56)(Si.75Al.25)8O36Cl2
**Name**	Calcium Magnesium Aluminum Silicate Chloride
**Name (mineral)**	
**Name (common)**	
**Status**	Primary
**Ambient**	Yes

**Lattice:**	Tetragonal	**Mol. weight =**	1710.74
**S.G.:**	I4/mmm (139)	**Volume [CD] =**	1158.12
		**Dx =**	
		**Dm =**	
		**I/Icor =**	2.740

a =	10.46100	
c =	10.58300	
a/b =	1.00000	Z = 1
c/b =	1.01166	

ICSD Collection Code: 063411
Temperature Factor: ITF
General Comments: earing
Additional Pattern: See PDF 41-246
Article Title: Atomic structures of alinite and jasmundite

Structure
Publication: Kristallografiya
Detail: volume 34, page 71 (1989)
Authors: Il'inets, A.M., Bikbau, M.Y., Nudel'man, B.I., Bolotina, N.B.
Primary Reference
Publication: Calculated from ICSD using POWD-12++

**Radiation:**	CuKa1	**Filter:**	Not specified
**Wavelength:**	1.54060	**d-spacing:**	
**SS/FOM:**	F(30)= 194.1 (0.0043, 36)		

d	2θ	I fix	h	k	l	d	2θ	I fix	h	k	l
7.43980	11.886	39	1	0	1	2.05976	43.922	78	4	1	3
7.43980	11.886	39	1	1	0	2.05248	44.086	54	5	0	1
5.29150	16.741	3	0	0	2	2.05248	44.086	54	5	1	0
5.23050	16.938	1	2	0	0	1.92842	47.087	51	2	1	5
4.27887	20.742	37	2	1	1	1.91283	47.494	24	5	1	2
3.71990	23.902	12	2	0	2	1.91283	47.494	24	5	2	1
3.69852	24.042	11	2	2	0	1.85995	48.932	127	4	0	4
3.34272	26.646	18	1	0	3	1.84926	49.233	149	4	4	0
3.31186	26.899	226	3	0	1	1.80936	50.394	54	3	0	5
3.31186	26.899	226	3	1	0	1.80380	50.560	37	3	3	4
3.03143	29.441	999	2	2	2	1.79951	50.689	64	5	0	3
2.81665	31.743	67	2	1	3	1.79405	50.854	37	5	3	0
2.80502	31.878	85	3	1	2	1.76383	51.790	30	0	0	6
2.79811	31.959	104	3	2	1	1.75245	52.151	21	4	2	4
2.64575	33.853	161	0	0	4	1.74350	52.439	35	4	4	2
2.61525	34.260	177	4	0	0	1.74350	52.439	35	6	0	0
2.49119	36.023	25	1	1	4	1.71573	53.354	3	1	1	6
2.47993	36.192	73	3	0	3	1.70994	53.549	39	3	2	5
2.46725	36.385	37	4	1	1	1.70162	53.832	74	5	2	3
2.46725	36.385	37	3	3	0	1.69751	53.973	43	5	3	2
2.36090	38.086	6	2	0	4	1.69751	53.973	43	6	1	1
2.34453	38.362	45	4	0	2	1.67136	54.888	2	2	0	6
2.33915	38.454	77	4	2	0	1.65403	55.512	15	6	0	2
2.24082	40.212	13	3	2	3	1.65403	55.512	15	6	2	0
2.23496	40.322	24	3	3	2	1.62529	56.582	14	4	1	5
2.15185	41.951	13	2	2	4	1.62126	56.735	10	5	1	4
2.13943	42.206	21	4	2	2	1.59206	57.873	22	2	2	6
2.07456	43.593	16	1	0	5	1.57870	58.410	105	6	2	2
2.06620	43.778	24	3	1	4	1.55641	59.329	1	3	1	6

d	2θ	I fix	h	k	l
1.54586	59.775	20	6	1	3
1.54277	59.907	11	6	3	1
1.51572	61.089	31	4	4	4
1.49631	61.968	18	1	0	7
1.48796	62.355	19	4	3	5
1.48486	62.499	16	5	3	4
1.47975	62.740	12	7	0	1
1.47975	62.740	12	7	1	0
1.46233	63.574	1	4	0	6
1.45582	63.892	8	6	0	4
1.45068	64.145	4	6	4	0
1.43860	64.749	15	2	1	7
1.43457	64.953	11	3	3	6
1.43118	65.126	6	5	2	5
1.42477	65.456	29	5	5	2
1.42477	65.456	29	7	2	1
1.40833	66.317	6	4	2	6
1.39906	66.814	3	6	4	2
1.38709	67.468	1	3	0	7
1.37605	68.083	2	7	0	3
1.37360	68.221	4	7	3	0
1.34075	70.133	1	3	2	7
1.33748	70.330	1	5	1	6
1.33473	70.497	8	6	1	5
1.33076	70.738	10	7	2	3
1.32953	70.814	6	7	3	2
1.32953	70.814	6	6	5	1
1.32288	71.224	3	0	0	8
1.30763	72.184	16	8	0	0
1.29876	72.755	4	4	1	7
1.29329	73.113	1	5	4	5
1.29125	73.247	3	7	1	4
1.28788	73.470	10	8	1	1
1.27635	74.245	8	4	4	6
1.27202	74.540	10	6	4	4
1.26944	74.717	11	8	0	2
1.26944	74.717	11	8	2	0
1.25548	75.693	6	6	3	5
1.25218	75.928	6	6	5	3
1.24560	76.401	23	2	2	8
1.23997	76.811	7	6	0	6
1.23363	77.279	7	8	2	2
1.23363	77.279	7	6	6	0
1.22540	77.895	24	4	3	7
1.22080	78.245	20	7	0	5
1.21909	78.376	19	7	3	4
1.21777	78.477	23	8	1	3
1.21607	78.608	15	8	3	1
1.21607	78.608	15	7	5	0
1.20653	79.352	3	6	2	6
1.20068	79.815	15	6	6	2
1.19310	80.426	21	5	2	7
1.18885	80.772	13	7	2	5
1.18517	81.076	8	7	5	2
1.17227	82.158	6	8	0	4
1.16958	82.388	18	8	4	0
1.16958	82.388	18	1	0	9
1.16570	82.723	8	3	3	8
1.15668	83.512	4	8	3	3
1.15522	83.641	2	9	0	1
1.15522	83.641	2	9	1	0
1.15149	83.974	3	4	2	8
1.14389	84.661	6	8	2	4
1.14042	84.979	6	2	1	9
1.13548	85.436	3	6	1	7
1.13349	85.622	4	7	1	6
1.13182	85.779	10	6	5	5
1.12864	86.079	8	9	1	2
1.12864	86.079	8	7	6	1
1.12041	86.868	1	6	4	6
1.11748	87.152	1	6	6	4
1.11424	87.470	2	3	0	9
1.11179	87.712	1	5	1	8
1.10963	87.926	4	5	4	7
1.10621	88.268	12	8	1	5
1.10494	88.396	6	7	5	4
1.10395	88.496	8	9	0	3
1.10395	88.496	8	9	3	0
1.08979	89.956	3	3	2	9

Table S33 Patterns from the SSA obtained from BNP. Pattern: COD 9006551, Radiation: 1.54060

Formula	AlO4P
Name	
Name (mineral)	Berlinite
Name (common)	
Status	Status Unknown
Ambient	Yes

Lattice:	Hexagonal	Mol. weight =	
S.G.:	P 31 2 1 S (152)	Volume [CD] =	213.26
		Dx =	
		Dm =	
		I/Icor =	3.130

a =	4.79620
c =	10.70500
a/b =	1.00000
c/b =	2.23198

Primary Reference
Christie D. M., Chelikowsky J. R., "Structural properties of alpha-berlinite (AlPO4)Sample: V = 71.09, theoretical", Physics and Chemistry of Minerals 25 (1998) 222-226.

Wavelength:	1.54060	Filter:	Not specified
SS/FOM:		d-spacing:	

d	2θ	I fix	h	k	l
4.15360	21.375	219	1	0	0
3.87240	22.948	31	1	0	1
3.56830	24.934	11	0	0	3
3.28150	27.153	1000	1	0	2
2.39810	37.473	109	1	1	0
2.24970	40.046	121	1	0	4
2.18850	41.216	54	1	1	2
2.07680	43.543	78	2	0	0
1.93620	46.887	23	2	0	2
1.90310	47.752	2	1	0	5
1.78600	51.100	85	1	1	4
1.78420	51.155	12	0	0	6
1.64070	56.003	58	2	0	4
1.63930	56.055	21	1	0	6
1.59710	57.673	2	1	1	5
1.56990	58.769	2	2	1	0
1.55330	59.460	4	2	1	1
1.50650	61.503	97	2	1	2
1.49070	62.227	2	2	0	5
1.43700	64.830	1	2	1	3
1.43510	64.926	1	1	0	7
1.43150	65.110	23	1	1	6
1.35410	69.342	62	2	1	4
1.35330	69.389	53	2	0	6
1.34040	70.154	60	3	0	2
1.27370	74.425	35	1	0	8
1.22970	77.572	29	3	0	4
1.19900	79.950	8	2	2	0
1.17860	81.623	30	2	1	6
1.17010	82.344	12	2	2	2
1.16850	82.481	16	1	1	8
1.15200	83.928	31	3	1	0
1.12620	86.311	7	3	1	2
1.12490	86.435	2	2	0	8
1.09380	89.537	2	3	0	6
1.05810	93.438	28	3	1	4
1.03840	95.772	2	4	0	0
1.03660	95.993	12	1	0	10
1.01940	98.163	13	4	0	2
1.01840	98.293	13	2	1	8

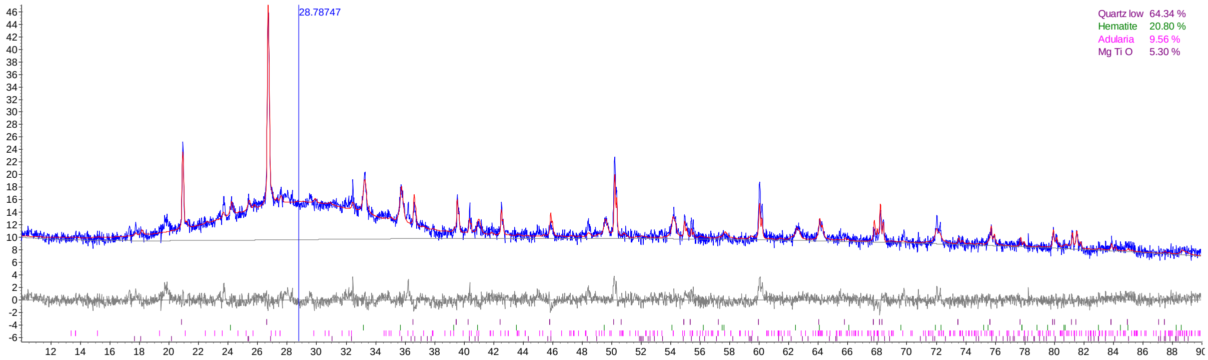

Fig. S36 Raw diffractogram of the SSA obtained from BP

Table S34 Analysis from the SSA obtained from BP

R-Values

Rexp : 8.84	Rwp : 11.65	Rp : 9.07	GOF : 1.32
Rexp`: 17.28	Rwp`: 22.79	Rp` : 20.07	DW : 1.22

Quantitative Analysis - Rietveld

Phase 1	: "Quartz low"	64.338 %
Phase 2	: Hematite	20.800 %
Phase 3	: Adularia	9.558 %
Phase 4	: "Mg Ti O"	5.304 %

**Degree of crystallinity (%)**	37.05
Crystalline area	1149.155
Amorphous area	1952.806

Background

One on X		600.0165
Chebychev polynomial, Coefficient	0	62.5841
	1	0.9413346
	2	-21.66796

Instrument

Primary radius (mm)	250
Secondary radius (mm)	250
Receiving slit width (mm)	0.3
FDS angle (°)	0.5563
Beam spill, Sample length (mm)	25
Intensity corrected	
Full Axial Convolution	
Filament length (mm)	16
Sample length (mm)	25
Receiving Slit length (mm)	16
Primary Sollers (°)	2.5
Secondary Sollers (°)	2.5

Corrections

Specimen displacement	-0.1794499
LP Factor	26.4

Miscellaneous

Convolution Steps	4

Table S35 Analysis from the SSA obtained from BP - Structures 1-4

Structure 1

Phase name	Quartz low
R-Bragg	7.079
Spacegroup	P3221
Scale	7.80092e-004
Cell Mass	180.253
Cell Volume (Å^3)	113.04587
Wt% - Rietveld	64.338
Double-Voigt|Approach	
Cry size Lorentzian	319.6
Cry size Gaussian	272.8
k: 1 LVol-IB (nm)	133.500
k: 0.89 LVol-FWHM (nm)	158.070
Crystal Linear Absorption Coeff. (1/cm)	94.823
Crystal Density (g/cm^3)	2.648
Preferred Orientation Spherical Harmonics	
Order	6
y00	1
y20	0.003805505
y40	-0.4813003
y43m	0.8158364
y60	-0.650594
y63m	0.7199932
y66p	0.01986087
Lattice parameters	
a (Å)	4.9143394
c (Å)	5.4049760

Site	Np	x	y	z	Atom	Occ	Beq
Si1	3	0.47230	0.00000	0.66667	Si+4	1	0.793
O1	6	0.41600	0.26580	0.78810	O-2	1	1.479

Structure 2

Phase name	Hematite
R-Bragg	2.433
Spacegroup	R-3cH
Scale	1.77890e-005
Cell Mass	958.129
Cell Volume (Å^3)	301.51033
Wt% - Rietveld	20.800
Double-Voigt|Approach	
Cry size Lorentzian	54.9
Cry size Gaussian	9999.0
k: 1 LVol-IB (nm)	35.003
k: 0.89 LVol-FWHM (nm)	48.889
Crystal Linear Absorption Coeff. (1/cm)	1130.623
Crystal Density (g/cm^3)	5.277
Preferred Orientation Spherical Harmonics	
Order	8
y00	1
y20	0.8008116
y40	1.262668
y43m	-0.001760463
y60	0.4647607
y63m	0.4241197
y66p	0.4284938
y80	0.3568643
y83m	-1.018079
y86p	0.2985378
Lattice parameters	
a (Å)	5.0320059

c (Å) 13.7495754

Site	Np	x	y	z	Atom	Occ	Beq
Fe1	12	0.00000	0.00000	0.35528	Fe+3	1	1
O1	18	0.69397	0.00000	0.25000	O-2	1	1

Structure 3

Phase name	Adularia
R-Bragg	4.195
Spacegroup	C12/m1
Scale	2.95553e-006
Cell Mass	1113.238
Cell Volume (Å^3)	717.75169
Wt% - Rietveld	9.558
Double-Voigt|Approach	
Cry size Lorentzian	85.1
Cry size Gaussian	9999.0
k: 1 LVol-IB (nm)	54.296
k: 0.89 LVol-FWHM (nm)	75.807
Crystal Linear Absorption Coeff. (1/cm)	127.759
Crystal Density (g/cm^3)	2.576
Lattice parameters	
a (Å)	8.5425314
b (Å)	12.9647064
c (Å)	7.1885392
beta (°)	115.639

Site	Np	x	y	z	Atom	Occ	Beq
O1	4	0.00000	0.14470	0.00000	O-2	1	1
O2	4	0.63390	0.00000	0.28480	O-2	1	1
O3	8	0.82620	0.14490	0.22800	O-2	1	1
O4	8	0.03390	0.31170	0.25850	O-2	1	1
O5	8	0.18210	0.12570	0.40700	O-2	1	1
Al1	8	0.00930	0.18420	0.22430	Al+3	0.4	1
Si1	8	0.00930	0.18420	0.22430	Si+4	0.6	1
Al2	8	0.70780	0.11760	0.34450	Al+3	0.11	1
Si2	8	0.70780	0.11760	0.34450	Si+4	0.89	1
K1	4	0.28340	0.00000	0.13720	K+1	1	1

Structure 4

Phase name	Mg Ti O
R-Bragg	6.252
Spacegroup	Ccmm
Scale	4.29783e-006
Cell Mass	823.800
Cell Volume (Å^3)	370.09191
Wt% - Rietveld	5.304
Double-Voigt|Approach	
Cry size Lorentzian	70.1
Cry size Gaussian	9990.7
k: 1 LVol-IB (nm)	44.711
k: 0.89 LVol-FWHM (nm)	62.435
Crystal Linear Absorption Coeff. (1/cm)	415.540
Crystal Density (g/cm^3)	3.696
Lattice parameters	
a (Å)	9.8003326
b (Å)	3.7619627
c (Å)	10.0381642

Site	Np	x	y	z	Atom	Occ	Beq
Mg1	4	0.80640	0.00000	0.25000	Mg+2	0.487	0.28
Ti1	4	0.80640	0.00000	0.25000	Ti+3	0.513	0.28
Mg2	8	0.13510	0.00000	0.06370	Mg+2	0.131	0.33
Ti2	8	0.13510	0.00000	0.06370	Ti+3	0.869	0.33
O1	4	0.22890	0.00000	0.25000	O-2	1	0.61
O2	8	0.04660	0.00000	0.88390	O-2	1	0.68
O3	8	0.31220	0.00000	0.93210	O-2	1	0.6

Table S36 Analysis from the SSA obtained from BP - Peaks

Peaks Phase 1

Phase name Peaks Phase:0

Type	Position	I		
FP	28.78747	76.71429	Cry size Lor	2.0
			Cry size Gauss	1.1
			k: 1, LVol-IB(nm)	0.674
			k: 0.89, LVol-FWHM(nm)	0.759

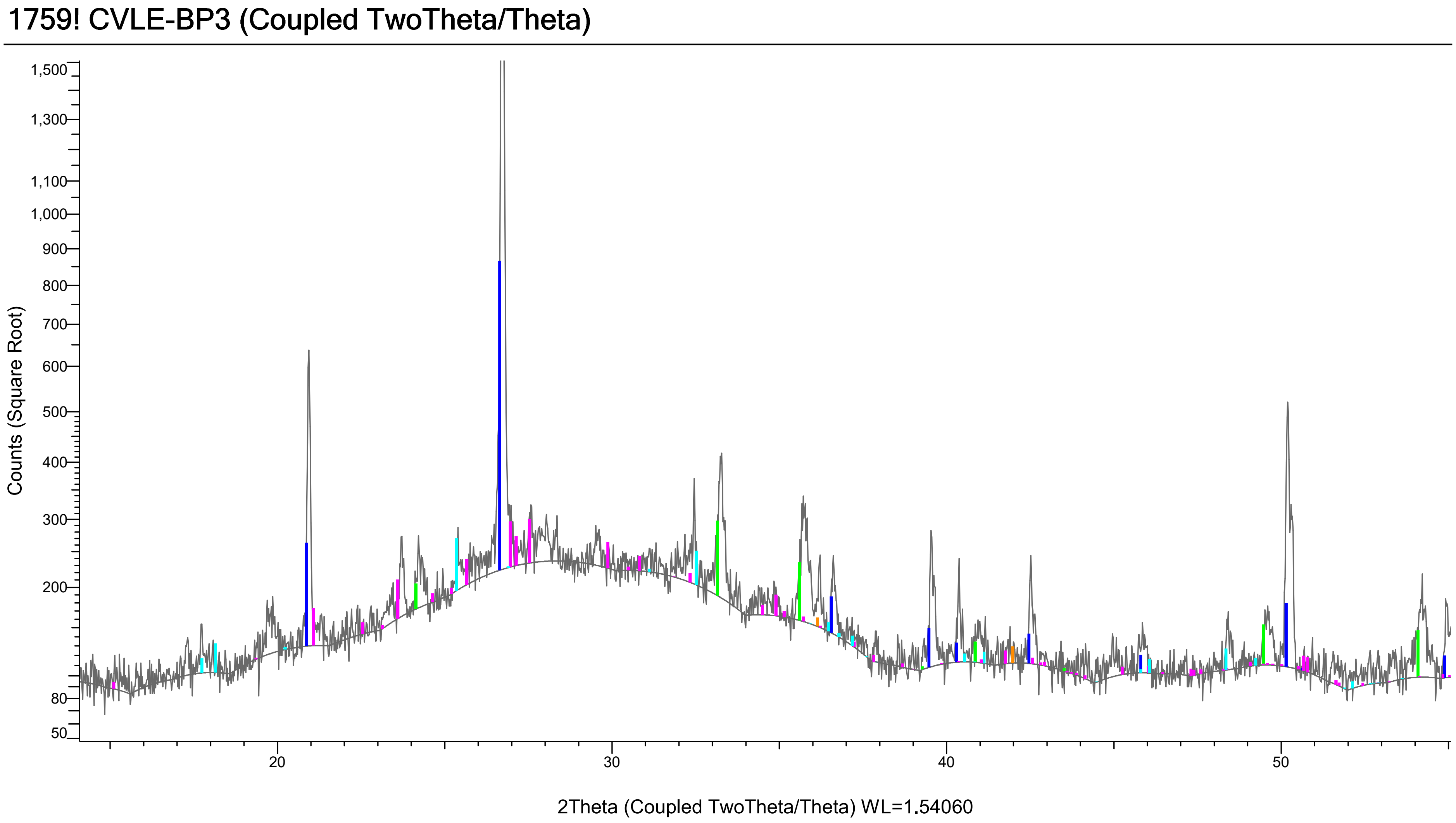

Fig. S37 High definition diffractogram of the SSA obtained from BP

Table S37 Patterns from the SSA obtained from BP. Pattern: PDF 78-2315, Radiation: 1.54060

Formula	SiO2
Name	Silicon Oxide
Name (mineral)	Quartz
Name (common)	
Status	Primary
Ambient	Yes

Lattice:	Hexagonal	Mol. weight =	60.08
S.G.:	P3221 (154)	Volume [CD] =	112.93
		Dx =	
		Dm =	
		I/Icor =	3.100

a =	4.91239	Z =	3
c =	5.40385		
a/b =	1.00000		
c/b =	1.10004		

ICSD Collection Code: 063532
Additional Pattern: See PDF 87-2096, PDF 85-335, PDF 85-457, PDF 85-504, PDF 85-695, PDF 85-794, PDF 85-795, PDF 85-796, PDF 85-797, PDF 85-798, PDF 85-930, PDF 85-1053, PDF 85-1054, PDF 85-1780, PDF 86-1560 and PDF 86-1561
Article Title: Crystal structures of quartz and magnesium germanate by profile analysis of synchrotron-radiation high-resolution powder data

Structure
Publication: J. Appl. Crystallogr.
Detail: volume 21, page 182 (1988)
Authors: Will, G., Bellotto, M., Parrish, W., Hart, M.
Primary Reference
Publication: Calculated from ICSD using POWD-12++

Radiation:	CuKa1	Filter:	Not specified
Wavelength:	1.54060	d-spacing:	
SS/FOM:	F(29)= 999.9 (0.0001, 29)		

d	2θ	I fix	h	k	l
4.25425	20.864	208	1	0	0
3.34268	26.646	999	0	1	1
2.45620	36.554	68	1	1	0
2.28080	39.478	65	1	0	2
2.23605	40.302	32	1	1	1
2.12713	42.462	49	2	0	0
1.97930	45.807	28	2	0	1
1.81747	50.153	110	1	1	2
1.80128	50.636	4	0	0	3
1.67134	54.889	34	0	2	2
1.65873	55.342	15	0	1	3
1.60796	57.247	2	2	1	0
1.54118	59.975	84	1	2	1
1.45254	64.053	14	1	1	3
1.41808	65.803	4	3	0	0
1.38178	67.762	46	1	2	2
1.37463	68.163	59	2	0	3
1.37164	68.332	62	0	3	1
1.28760	73.489	18	1	0	4
1.25565	75.681	26	3	0	2
1.22810	77.692	14	2	2	0
1.19955	79.906	25	2	1	3
1.19756	80.065	14	2	2	1
1.18372	81.196	20	1	1	4
1.17992	81.512	26	3	1	0
1.15276	83.860	15	1	3	1
1.14040	84.980	2	2	0	4
1.11803	87.099	1	2	2	2
1.11423	87.471	2	3	0	3

Table S38 Patterns from the SSA obtained from BP. Pattern: PDF 33-0664, Radiation: 1.54060

Formula	Fe_2O_3
Name	Iron Oxide
Name (mineral)	Hematite, syn
Name (common)	burnt ochre, colcothar, rouge
Status	Primary
Ambient	Yes

Lattice:	Rhombo.H.axes	Mol. weight =	159.69
S.G.:	R-3c (167)	Volume [CD] =	301.93
		Dx =	
		Dm =	5.26
		I/Icor =	2.400

a =	5.03560	Z = 6
c =	13.74890	
a/b =	1.00000	
c/b =	2.73034	

Optical Data: A=2.94, B=3.22, Sign=-
Melting Point: 1350-1360°
Color: Dark reddish brown
Sample Source Or Locality: Sample from Pfizer, Inc., New York, USA, heated at 800 C for 3 days
Additional Pattern: To replace 13-534 and validated by calculated pattern 24-72
General Comments: Opaque mineral optical data on specimen from Elba, R#1R#0=30.2, RR#2R#e=26.1, Disp.=16, VHN=1038 (mean at 100, 200, 300), Color values=1 .299, .309, 29.8, 2 .299, .309, 25.7
Additional Pattern: See ICSD 64599 (PDF 79-7)
General Comments: Pattern reviewed by Syvinski, W., McCarthy, G., North Dakota State Univ., Fargo, North Dakota, USA, ICDD Grant-in-Aid (1990). Agress well with experimental and calculated patterns
General Comments: Additional weak reflection [indicated by brackets] was observed

Primary Reference
Publication: Natl. Bur. Stand. (U.S.) Monogr. 25
Detail: volume 18, page 37 (1981)

Radiation:	CuKa1	Filter:	M
Wavelength:	1.54060	d-spacing:	
SS/FOM:	F(30)= 69.3 (0.0111, 39)		

d	2θ	I fix	h	k	l
3.68400	24.138	31	0	1	2
2.70000	33.153	101	1	0	4
2.51900	35.612	70	1	1	0
2.29200	39.277	4	0	0	6
2.20700	40.855	21	1	1	3
2.07790	43.519	4	2	0	2
1.84060	49.480	41	0	2	4
1.69410	54.091	46	1	1	6
1.63670	56.152	2	2	1	1
1.60330	57.429	6	1	2	2
1.59920	57.590	11	0	1	8
1.48590	62.451	31	2	1	4
1.45380	63.991	31	3	0	0
1.41380	66.028	2	1	2	5
1.34970	69.601	4	2	0	8
1.31150	71.937	11	1	0	10
1.30640	72.262	7	1	1	9
1.25920	75.430	9	2	2	0
1.22760	77.730	5	3	0	6
1.21410	78.760	3	2	2	3
1.18960	80.711	6	1	2	8
1.16320	82.940	6	0	2	10
1.14110	84.916	8	1	3	4
1.10350	88.542	8	2	2	6
1.07680	91.345	3	0	4	2
1.05570	93.715	8	2	1	10
1.04280	95.239	2	1	1	12
1.03930	95.663	4	4	0	4
0.98920	102.285	5	3	1	8
0.97150	104.914	2	2	2	9
0.96060	106.623	6	3	2	4
0.95810	107.025	5	0	1	14
0.95160	108.090	6	4	1	0
0.93180	111.518	3	4	1	3
0.92060	113.594	3	0	4	8
0.90810	116.045	6	1	3	10
0.89980	117.758	2	3	0	12
0.89540	118.697	4	2	0	14
0.87890	122.431	7	4	1	6
0.86480	125.929	2	2	3	8
0.85430	128.758	4	4	0	10
0.84360	131.878	6	1	2	14
0.83920	133.241	4	3	3	0
0.80890	144.457	5	3	2	10
0.80140	147.971	5	2	4	4

Table S39 Patterns from the SSA obtained from BP. Pattern: PDF 71-1543, Radiation: 1.54060

Formula	K(AlSi3O8)
Name	Potassium Aluminum Silicate
Name (mineral)	Adularia
Name (common)	
Status	Primary
Ambient	Yes

Lattice:	Monoclinic	Mol. weight =	278.33
S.G.:	C2/m (12)	Volume [CD] =	717.14
		Dx =	
		Dm =	
		I/Icor =	0.750

a =	8.54500		
b =	12.96700	beta =	116.000
c =	7.20100		
a/b =	0.65898	Z =	4
c/b =	0.55533		

ICSD Collection Code: 010273
Temperature Factor: ATF
Remark From ICSD/CSD: REM M PDF 31-966
Sample Source Or Locality: Specimen from St. Gotthard, Switzerland
Test From ICSD: Charge sum slightly deviates from zero
Article Title: The structures of monoclinic potassium rich feldspars

Structure
Publication: Am. Mineral.
Detail: volume 58, page 263 (1973)
Authors: Phillips, M.W., Ribbe, P.H.
Primary Reference
Publication: Calculated from ICSD using POWD-12++

Radiation:	CuKa1	Filter:	Not specified
Wavelength:	1.54060	d-spacing:	
SS/FOM:	F(30)= 210.4 (0.0039, 37)		

d	2θ	I fix	h	k	l	d	2θ	I fix	h	k	l
6.60809	13.388	61	1	1	0	2.37648	37.826	103	-3	3	1
6.48350	13.647	90	0	2	0	2.32597	38.680	53	-1	1	3
6.48350	13.647	90	0	0	1	2.30689	39.013	3	-2	4	2
5.85858	15.111	92	-1	1	1	2.29027	39.308	3	0	4	2
4.58053	19.363	23	0	2	1	2.26011	39.854	20	-3	3	2
4.21039	21.084	626	-2	0	1	2.23258	40.367	13	-2	2	3
3.94022	22.547	198	1	1	1	2.23258	40.367	13	1	3	2
3.84010	23.143	59	2	0	0	2.19716	41.047	53	1	5	1
3.76677	23.600	726	1	3	0	2.16117	41.762	195	0	6	0
3.61047	24.638	172	-1	3	1	2.16117	41.762	195	-3	1	3
3.53114	25.200	121	-2	2	1	2.12182	42.574	89	2	4	1
3.46867	25.662	517	-1	1	2	2.11018	42.820	39	-4	0	1
3.30405	26.964	1000	2	2	0	2.10520	42.926	61	-4	0	2
3.28353	27.136	641	-2	0	2	2.10520	42.926	61	-1	5	2
3.23611	27.541	980	0	4	0	2.07428	43.599	9	-1	3	3
3.23611	27.541	980	0	0	2	2.06789	43.740	23	2	0	2
2.98821	29.877	561	1	3	1	2.06147	43.884	32	3	1	1
2.92929	30.492	81	-2	2	2	2.04990	44.144	46	0	6	1
2.89850	30.824	310	0	4	1	2.00229	45.251	115	-4	2	2
2.89850	30.824	310	0	2	2	1.97011	46.033	103	2	2	2
2.80649	31.861	5	2	0	1	1.95286	46.463	29	-3	3	3
2.76614	32.338	189	-1	3	2	1.92005	47.305	105	4	0	0
2.59772	34.499	179	-3	1	2	1.92005	47.305	105	-2	4	3
2.56861	34.902	384	-2	4	1	1.91663	47.394	74	-3	5	1
2.55101	35.151	107	1	1	2	1.90884	47.600	61	-4	0	3
2.51158	35.721	91	3	1	0	1.88339	48.284	27	2	6	0
2.47711	36.235	36	2	4	0	1.88014	48.372	27	3	3	1
2.41212	37.247	91	-1	5	1	1.85483	49.076	63	1	1	3
2.37648	37.826	103	-2	0	3	1.85483	49.076	63	-3	5	2

d	2θ	I fix	h	k	l	d	2θ	I fix	h	k	l	d	2θ	I fix	h	k	l	d	2θ	I fix	h	k	l
1.84102	49.468	22	4	2	0	1.60439	57.386	9	2	0	3	1.43539	64.912	46	4	6	0	1.30673	72.241	18	-4	6	4
1.83679	49.590	27	1	5	2	1.60238	57.465	8	4	2	1	1.43069	65.151	27	-2	0	5	1.30673	72.241	18	-5	1	5
1.83113	49.754	21	-4	2	3	1.59936	57.584	8	-2	6	3	1.43069	65.151	27	-4	6	3	1.30253	72.511	27	-6	4	2
1.82191	50.023	27	3	5	0	1.59153	57.894	48	-4	2	4	1.42621	65.381	27	-3	1	5	1.30253	72.511	27	5	3	1
1.80524	50.517	49	-2	6	2	1.58816	58.028	27	-5	3	2	1.42621	65.381	27	-5	5	2	1.30093	72.614	26	2	0	4
1.79993	50.676	219	1	7	0	1.56990	58.769	54	0	2	4	1.42255	65.571	65	-6	0	2	1.29886	72.749	18	-6	2	4
1.79993	50.676	219	-2	0	4	1.56667	58.902	29	3	3	2	1.41608	65.908	14	1	9	0	1.29589	72.942	26	0	8	3
1.79603	50.794	216	0	6	2	1.55741	59.287	15	2	2	3	1.41058	66.198	8	3	5	2	1.29589	72.942	26	0	6	4
1.79603	50.794	216	0	4	3	1.55741	59.287	15	-5	3	1	1.40731	66.372	8	-1	9	1	1.28778	73.477	21	-6	4	3
1.76851	51.642	59	-4	4	1	1.55238	59.499	9	-3	7	1	1.40325	66.589	58	-6	0	3	1.28778	73.477	21	4	4	2
1.76557	51.735	46	-4	4	2	1.52918	60.495	66	-5	3	3	1.40325	66.589	58	4	0	2	1.28430	73.709	82	-4	8	2
1.74339	52.443	35	-3	1	4	1.52684	60.597	43	0	6	3	1.39306	67.140	23	-4	0	5	1.27989	74.005	46	6	0	0
1.74339	52.443	35	2	4	2	1.52684	60.597	43	5	1	0	1.38950	67.335	14	-6	2	2	1.27989	74.005	46	-4	4	5
1.72953	52.895	9	-1	1	4	1.51899	60.943	12	1	5	3	1.38307	67.690	54	-2	6	4	1.27569	74.289	84	2	8	2
1.71943	53.231	14	1	3	3	1.51899	60.943	12	-3	7	2	1.38307	67.690	54	-5	5	3	1.27569	74.289	84	2	2	4
1.69299	54.129	17	-5	1	2	1.50983	61.353	65	-4	6	1	1.37573	68.101	9	-6	0	1	1.27191	74.548	43	1	5	4
1.67278	54.838	54	-3	5	3	1.50983	61.353	65	1	7	2	1.37164	68.332	23	-1	1	5	1.26939	74.721	13	-1	7	4
1.66703	55.043	31	3	1	2	1.50075	61.765	6	3	7	0	1.37164	68.332	23	3	1	3	1.26939	74.721	13	0	2	5
1.65416	55.508	7	-5	1	1	1.49330	62.107	170	2	6	2	1.36197	68.885	18	-3	3	5	1.26641	74.927	38	-6	4	1
1.65416	55.508	7	4	0	1	1.49330	62.107	170	2	8	0	1.36197	68.885	18	-4	2	5	1.26641	74.927	38	-3	9	2
1.65202	55.586	7	4	4	0	1.47606	62.914	27	-5	1	4	1.35851	69.085	11	5	1	1	1.26043	75.344	7	1	9	2
1.64714	55.765	26	-1	7	2	1.47308	63.056	20	4	4	1	1.34577	69.834	20	-6	2	1	1.25639	75.629	54	-3	5	5
1.64176	55.964	15	-4	0	4	1.45817	63.777	22	-1	7	3	1.33762	70.322	39	-1	9	2	1.25639	75.629	54	-5	7	2
1.62994	56.406	13	-3	3	4	1.45343	64.009	18	-2	8	2	1.32573	71.047	21	-6	0	4	1.24484	76.456	8	3	7	2
1.62639	56.540	62	3	5	1	1.45033	64.162	44	1	1	4	1.32162	71.302	15	5	5	0	1.23856	76.915	5	-2	10	1
1.62087	56.750	46	-5	1	3	1.45033	64.162	44	0	8	2	1.31746	71.562	8	1	7	3	1.23856	76.915	5	4	8	0
1.62087	56.750	46	0	8	0	1.44736	64.310	46	0	4	4	1.31409	71.774	45	-1	3	5	1.23553	77.138	6	-4	8	3
1.61821	56.852	21	-1	3	4	1.44736	64.310	46	5	3	0	1.31409	71.774	45	4	6	1	1.23033	77.525	2	-1	9	3
1.61821	56.852	21	0	0	4	1.43792	64.783	69	2	4	3	1.30923	72.082	20	-2	4	5	1.22709	77.768	5	-6	4	4

d	2θ	I fix	h	k	l
1.21729	78.514	14	-1	5	5
1.21729	78.514	14	3	5	3
1.21447	78.731	14	-6	0	5
1.21168	78.948	6	-7	1	3
1.20862	79.187	12	5	5	1
1.20862	79.187	12	-7	1	2
1.20606	79.389	17	-2	10	2
1.20367	79.578	16	0	10	2
1.20367	79.578	16	-3	9	3
1.19715	80.098	6	4	0	3
1.19485	80.284	23	-3	1	6
1.19323	80.415	17	-6	2	5
1.19323	80.415	17	-2	6	5
1.19058	80.631	16	6	4	0
1.18824	80.823	14	1	1	5
1.18824	80.823	14	-6	6	2
1.18586	81.018	7	3	9	1
1.18216	81.325	7	5	7	0
1.18216	81.325	7	-2	0	6
1.17692	81.764	17	-6	6	3
1.17692	81.764	17	4	6	2
1.17464	81.957	14	-7	1	4
1.17146	82.227	18	-7	3	3
1.17146	82.227	18	-4	6	5

Table S40 Patterns from the SSA obtained from BP. Pattern: PDF 74-1885, Radiation: 1.54060

Formula	Fe.9712O
Name	Iron Oxide
Name (mineral)	Wstite, syn
Name (common)	
Status	Primary
Ambient	Yes

Lattice:	Cubic	Mol. weight =	70.24
S.G.:	Fm-3m (225)	Volume [CD] =	79.56
		Dx =	
		Dm =	
		I/Icor =	4.770

a =	4.30100	Z =	4
a/b =	1.00000		
c/b =	1.00000		

ICSD Collection Code: 027855
Test From ICSD: Calc. density unusual but tolerable
Test From ICSD: No R value given
Test From ICSD: At least one TF missing
Additional Pattern: See PDF 74-1880, PDF 74-1881, PDF 74-1882, PDF 74-1883, PDF 74-1884 and PDF 46-1312
Sample Preparation: Prepared at 1323 K for 1 day, rapidly cooled
Analysis: Upper limit of Fe-contents (76.72 wt.%)
Polymorphism/Phase Transition: Only metastable below 843 K
Article Title: An X-Ray Study of the Wuestite (Fe O) Solid Solutions

Structure
Publication: J. Chem. Phys.
Detail: volume 1, page 29 (1933)
Authors: Jette, E.R., Foote, F.
Primary Reference
Publication: Calculated from ICSD using POWD-12++

Radiation:	CuKa1	Filter:	Not specified
Wavelength:	1.54060	d-spacing:	
SS/FOM:	F(5)= 999.9 (0.0000, 5)		

d	2θ	I fix	h	k	l
2.48318	36.143	621	1	1	1
2.15050	41.979	1000	2	0	0
1.52063	60.871	459	2	2	0
1.29680	72.883	154	3	1	1
1.24159	76.693	113	2	2	2

Table S41 Patterns from the SSA obtained from BP. Pattern: PDF 82-1127, Radiation: 1.54060

Formula	(Mg0.75Ti2.25)O5
Name	Magnesium Titanium Oxide
Name (mineral)	
Name (common)	
Status	Primary
Ambient	Yes

Lattice:	Orthorhombic	Mol. weight =	206
S.G.:	Ccmm (63)	Volume [CD] =	366.91
		Dx =	
		Dm =	
		I/Icor =	1.990

a =	9.76470	
b =	3.76178	
c =	9.98855	
a/b =	2.59577	Z = 4
c/b =	2.65527	

ICSD Collection Code: 075183
Remark From ICSD/CSD: REM RVP
Temperature Factor: ITF
Article Title: Phase equilibria and structural studies on the solid solution MgTi2O5-Ti3O5

Structure
Publication: J. Solid State Chem.
Detail: volume 113, page 62 (1994)
Authors: Grey, I.E., Li, C., Madsen, I.C.
Primary Reference
Publication: Calculated from ICSD using POWD-12++

Radiation:	CuKa1	Filter:	Not specified
Wavelength:	1.54060	d-spacing:	
SS/FOM:	F(30)= 999.9 (0.0000, 33)		

d	2θ	I fix	h	k	l	d	2θ	I fix	h	k	l
4.99427	17.745	191	0	0	2	1.63747	56.123	138	5	1	2
4.88235	18.155	398	2	0	0	1.62745	56.500	17	6	0	0
4.38639	20.228	34	2	0	1	1.60627	57.313	33	6	0	1
3.51030	25.352	999	1	1	0	1.57568	58.532	26	2	0	6
3.31175	26.900	25	1	1	1	1.55263	59.488	185	3	1	5
2.87188	31.117	57	1	1	2	1.55263	59.488	185	2	2	3
2.75077	32.524	648	2	0	3	1.54737	59.711	107	6	0	2
2.49714	35.934	5	0	0	4	1.53743	60.136	144	5	1	3
2.46141	36.474	147	3	1	0	1.50418	61.608	41	1	1	6
2.44117	36.787	60	4	0	0	1.48993	62.263	20	4	2	0
2.41571	37.189	150	1	1	3	1.47363	63.030	2	4	2	1
2.38992	37.606	11	3	1	1	1.46213	63.584	18	6	0	3
2.37138	37.911	8	4	0	1	1.43594	64.884	35	2	2	4
2.22322	40.544	116	2	0	4	1.42775	65.302	57	4	2	2
2.20783	40.839	10	3	1	2	1.42389	65.501	49	5	1	4
2.19319	41.124	155	4	0	2	1.37897	67.919	37	3	1	6
2.03480	44.489	9	1	1	4	1.37538	68.120	27	4	0	6
1.97928	45.807	47	3	1	3	1.36964	68.445	20	2	0	7
1.96871	46.067	182	4	0	3	1.35998	69.000	70	4	2	3
1.88089	48.352	294	0	2	0	1.32189	71.285	17	1	1	7
1.84892	49.243	101	2	0	5	1.31854	71.494	52	2	2	5
1.76020	51.904	17	0	2	2	1.30919	72.084	11	5	1	5
1.75298	52.134	84	3	1	4	1.30793	72.164	10	7	1	0
1.73624	52.675	11	1	1	5	1.29686	72.879	34	7	1	1
1.73328	52.772	9	5	1	0	1.27949	74.032	2	4	2	4
1.72867	52.924	7	2	2	1	1.26526	75.007	54	7	1	2
1.70776	53.623	15	5	1	1	1.26175	75.252	40	6	0	5
1.66476	55.124	103	0	0	6	1.24660	76.329	57	0	2	6
1.65587	55.445	15	2	2	2	1.24371	76.538	49	1	3	0

d	2θ	I fix	h	k	l
1.23449	77.215	5	1	3	1
1.23449	77.215	5	3	1	7
1.23071	77.496	10	6	2	0
1.22147	78.194	20	6	2	1
1.20785	79.248	17	2	2	6
1.19496	80.275	3	4	2	5
1.19496	80.275	3	6	2	2
1.18569	81.033	10	8	0	2
1.17637	81.811	2	1	1	8
1.17010	82.344	11	3	3	0
1.16508	82.776	15	1	3	3
1.16375	82.892	8	6	0	6
1.16215	83.031	10	3	3	1
1.15862	83.341	4	7	1	4
1.15437	83.717	13	6	2	3
1.14601	84.468	2	8	0	3
1.13925	85.086	2	3	3	2
1.11350	87.543	5	1	3	4
1.11350	87.543	5	3	1	8
1.11161	87.730	21	4	0	8
1.11022	87.868	11	4	2	6
1.10719	88.170	18	2	2	7
1.10392	88.499	10	3	3	3
1.10392	88.499	10	6	2	4
1.10164	88.731	4	5	1	7
1.09426	89.489	17	7	1	5
